# Plasma 1,3-β-d-glucan levels predict adverse clinical outcomes in critical illness

**DOI:** 10.1172/jci.insight.141277

**Published:** 2021-07-22

**Authors:** Georgios D. Kitsios, Daniel Kotok, Haopu Yang, Malcolm A. Finkelman, Yonglong Zhang, Noel Britton, Xiaoyun Li, Marina S. Levochkina, Amy K. Wagner, Caitlin Schaefer, John J. Villandre, Rui Guo, John W. Evankovich, William Bain, Faraaz Shah, Yingze Zhang, Barbara A. Methé, Panayiotis V. Benos, Bryan J. McVerry, Alison Morris

**Affiliations:** 1 Division of Pulmonary, Allergy and Critical Care Medicine, Department of Medicine, University of Pittsburgh School of Medicine and University of Pittsburgh Medical Center, Pittsburgh, Pennsylvania, USA.; 2 Center for Medicine and the Microbiome and; 3 Acute Lung Injury Center of Excellence, Department of Medicine, University of Pittsburgh, Pittsburgh, Pennsylvania, USA.; 4 Department of Pulmonary and Critical Care Medicine, Cleveland Clinic Florida, Weston Hospital, Weston, Florida, USA.; 5 School of Medicine, Tsinghua University, Beijing, China.; 6 Department of Computational and Systems Biology, University of Pittsburgh, Pittsburgh, Pennsylvania, USA.; 7 Associates of Cape Cod Inc., East Falmouth, Massachusetts, USA.; 8 Department of Infectious Diseases and Microbiology and; 9 Departments of Physical Medicine and Rehabilitation, Neuroscience, and Clinical and Translational Science, Center for Neuroscience, Safar Center for Resuscitation Research, University of Pittsburgh, Pittsburgh, Pennsylvania, USA.; 10 Department of Emergency and Critical Care Medicine, First Affiliated Hospital of Chongqing Medical University, Chongqing, China.; 11 Veterans Affairs Pittsburgh Healthcare System, Pittsburgh, Pennsylvania, USA.; 12 Department of Immunology, University of Pittsburgh School of Medicine, Pittsburgh, Pennsylvania, USA.

**Keywords:** Infectious disease, Microbiology, Cytokines, Respiration

## Abstract

**BACKGROUND:**

The fungal cell wall constituent 1,3-β-d-glucan (BDG) is a pathogen-associated molecular pattern that can stimulate innate immunity. We hypothesized that BDG from colonizing fungi in critically ill patients may translocate into the systemic circulation and be associated with host inflammation and outcomes.

**METHODS:**

We enrolled 453 mechanically ventilated patients with acute respiratory failure (ARF) without invasive fungal infection and measured BDG, innate immunity, and epithelial permeability biomarkers in serially collected plasma samples.

**RESULTS:**

Compared with healthy controls, patients with ARF had significantly higher BDG levels (median [IQR], 26 pg/mL [15–49 pg/mL], *P <* 0.001), whereas patients with ARF with high BDG levels (≥40 pg/mL, 31%) had higher odds for assignment to the prognostically adverse hyperinflammatory subphenotype (OR [CI], 2.88 [1.83–4.54], *P <* 0.001). Baseline BDG levels were predictive of fewer ventilator-free days and worse 30-day survival (adjusted *P <* 0.05). Integrative analyses of fungal colonization and epithelial barrier disruption suggested that BDG may translocate from either the lung or gut compartment. We validated the associations between plasma BDG and host inflammatory responses in 97 hospitalized patients with COVID-19.

**CONCLUSION:**

BDG measurements offered prognostic information in critically ill patients without fungal infections. Further research in the mechanisms of translocation and innate immunity recognition and stimulation may offer new therapeutic opportunities in critical illness.

**FUNDING:**

University of Pittsburgh Clinical and Translational Science Institute, COVID-19 Pilot Award and NIH grants (K23 HL139987, U01 HL098962, P01 HL114453, R01 HL097376, K24 HL123342, U01 HL137159, R01 LM012087, K08HK144820, F32 HL142172, K23 GM122069).

## Graphical abstract

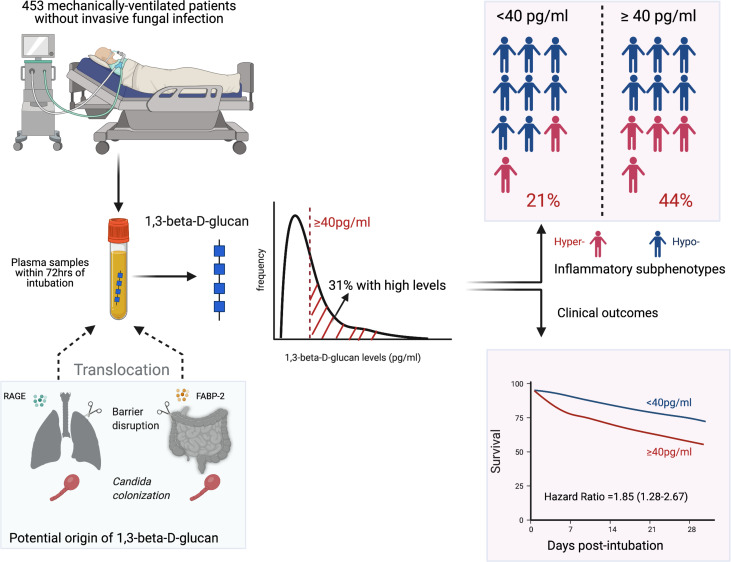

## Introduction

Dysregulation of innate immunity is a central pathogenetic feature of the heterogeneous syndromes of sepsis and acute respiratory distress syndrome (ARDS; refs. 1, 2). In several independent populations, patients with elevated systemic levels of inflammatory cytokines and biomarkers of tissue injury (classified as a hyperinflammatory subphenotype) exhibit worse organ dysfunction and clinical outcomes compared with their hypoinflammatory counterparts (3–5). Such biological subphenotyping of critically ill patients offers improved risk prediction over stratification with clinical variables alone and presents new opportunities for targeted pharmacological interventions (6). However, the molecular mechanisms of aberrant innate immunity stimulation and resultant tissue injury remain largely undefined.

Interpatient variability of endogenous microbiota may contribute to the observed differences in host responses. Many of the predictive biomarkers for the detrimental hyperinflammatory subphenotype (e.g., IL-6 and IL-8, soluble TNFR1, procalcitonin, and receptor for advanced glycation end products [RAGE]; refs. 3, 4) reflect canonical pathways of immune cell stimulation from pathogen- and damage-associated molecular patterns (PAMPs and DAMPs, respectively; refs. 7, 8). PAMPs include microbial nucleic acids as well as cell wall components, such as LPS of gram-negative bacteria and 1-3-β-d-glucan (BDG) from fungi (8, 9). Recent culture-independent studies with bacterial DNA sequencing have uncovered important roles of intestinal and respiratory bacterial communities in the evolution and outcome of critical illness (10–12). However, very few studies have performed in-depth examinations of fungal communities (mycobiome) in critical illness (13) and little is known about how fungal PAMPs may interact with innate immunity in the critically ill host.

Epidemiological evidence suggests that host-fungal interactions at different mucosal surfaces are clinically important (14). In mechanically ventilated immunocompromised patients, airway colonization by *Candida* species portends worse outcome (15). In patients with suspected ventilator-associated pneumonia, *Candida* species in respiratory tract secretions have been associated with increased levels of IL-6 and procalcitonin, as well as higher mortality (16, 17). *C*. *albicans* overgrowth in the human gut occurs in the setting of antibiotic-treated sepsis, raising the risk for secondary candidemia (18, 19). The respiratory and intestinal tracts in critical illness may thus contain high levels of the fungal PAMP BDG, which can be recognized by mucosal innate immune cells through the dectin-1 receptor and stimulate local inflammatory responses (20). BDG can also exert systemic effects via translocation into the bloodstream in the setting of disruption of mucosal epithelial integrity from various insults, such as hypoxemia, ischemia, or direct pathogen invasion (21). Preliminary observations have suggested that higher levels of circulating BDG are associated with worse clinical outcomes in patients in the intensive care unit (ICU; ref. 22), but evidence has been limited. BDG levels are routinely checked in the diagnostic workup of critically ill patients with clinical suspicion of invasive fungal infection (IFI; ref. 23), but the contribution of BDG to the development and perpetuation of hyperinflammatory host responses in critical illness and whether BDG could be used as a clinical biomarker of fungal PAMP translocation are unknown.

In this study, we demonstrated that critically ill patients without IFI had elevated plasma BDG levels compared with healthy controls. Elevated BDG levels correlated with plasma levels of circulating biomarkers of innate immunity and tissue injury and predicted worse clinical outcomes independent of other prognostic factors. Exploratory analyses for the putative source of BDG, i.e., the intestinal or respiratory tract, suggested the possibility of fungal PAMP translocation from either compartment in critically ill patients.

## Results

### Study population.

**Figure 1:**
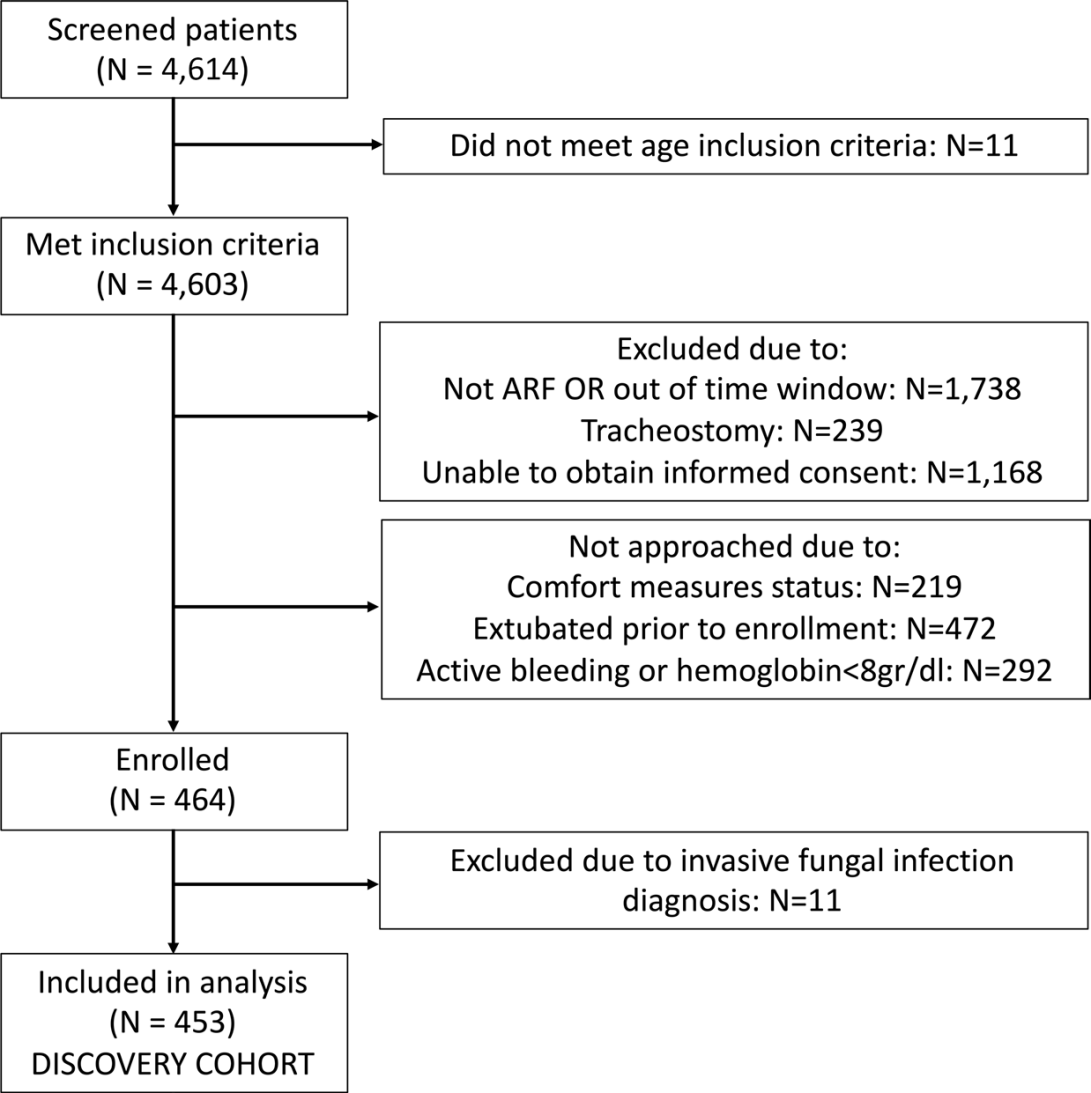
Flowchart of patient enrollment in the discovery cohort of patients with acute respiratory failure.

We analyzed data from 453 mechanically ventilated patients with acute respiratory failure (ARF; discovery cohort), who were consecutively enrolled (Figure 1) from ICUs at the University of Pittsburgh Medical Center (UPMC; refs. 3, 10, 24). We excluded 11 patients with a clinical diagnosis of IFI a priori because we wanted to examine BDG as a fungal PAMP in a broad ICU population and not in the context of IFI as a diagnostic test. Characteristics of patients with ARF in the discovery cohort are shown in Table 1. We also included data from 2 independent validation cohorts: (a) 97 patients with ARF from COVID-19 (25) and (b) 137 mechanically ventilated patients after severe traumatic brain injury (TBI; ref. 26). Characteristics of patients in the validation cohorts are shown in Supplemental Tables 2 and 3 (supplemental material available online with this article; https://doi.org/10.1172/jci.insight.141277DS1).

### Distribution of plasma BDG levels in critically ill patients.

**Figure 2:**
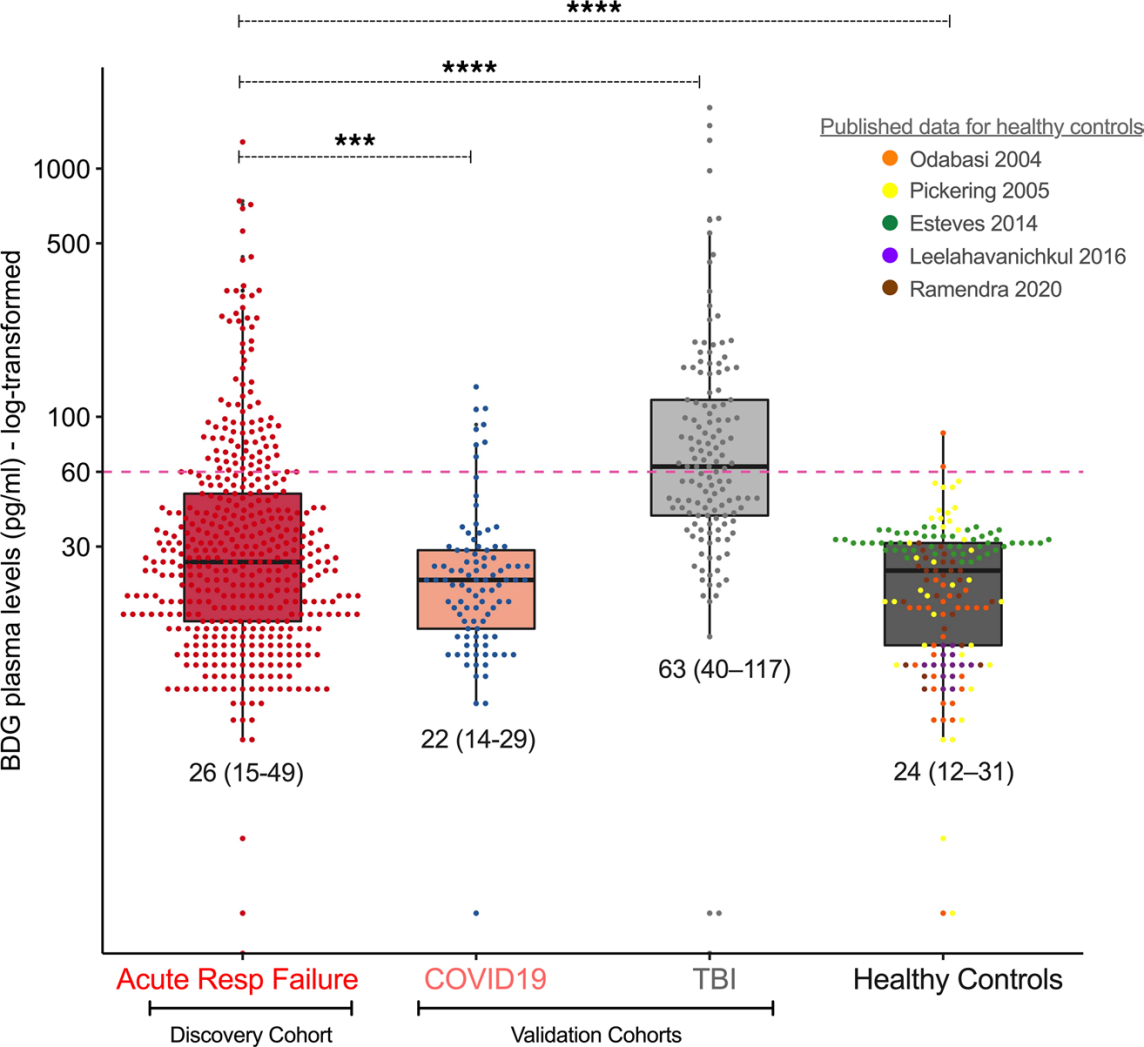
Mechanically ventilated patients with acute respiratory failure had higher BDG levels compared with inpatients with COVID-19 and healthy controls and lower levels compared with patients with traumatic brain injury. The conventional threshold of 60 pg/mL in the diagnostic workup of invasive fungal infection is shown with a red dashed line, distinguishing 81% of patients with acute respiratory failure with negative BDG levels versus 19% with intermediate or positive levels. We used published raw data for BDG values in healthy control groups. Displayed *P* values for comparisons of BDG levels between the discovery and validation cohorts were derived from nonparametric Wilcoxon’s tests, whereas for the comparison with healthy controls, we constructed mixed linear regression models with random study intercepts to account for potentially different BDG levels by study. Median and IQR BDG values by group are shown. ***P < 0.001; ****P < 0.0001.

We compared baseline BDG levels among patients with ARF (discovery cohort) and COVID-19 and TBI (validation cohorts) and publicly available data from 5 cohorts of healthy control patients (Figure 2 and Supplemental Figure 1 and refs. 27–31). Patients with ARF had higher BDG levels (median 26, IQR 15–49) compared with patients with COVID-19 (Wilcoxon’s test *P =* 0.005) and healthy controls (adjusted *P* value for mixed linear model with random study intercepts = 0.005), but lower levels compared with patients with TBI (*P <* 0.001). By applying standard cutoffs of BDG test positivity for the diagnosis of IFI in the ARF cohort (i.e., <60 pg/mL negative, 61–79 pg/mL indeterminate, ≥80 pg/mL positive), 81.2% of baseline samples were negative, 5.7% indeterminate, and 13.0% positive, with no significant differences in clinical characteristics between groups apart from higher sequential organ failure assessment (SOFA) scores in the indeterminate and positive group (*P =* 0.02; Supplemental Table 3). Although β-lactam antibiotics have been previously implicated in false-positive BDG detection (32), we did not find any differences in BDG levels between patients receiving β-lactam antibiotics or not receiving them at the time of sampling. A small proportion of patients (2.9%) receiving topical antifungals for either oral thrush or skin candidiasis (clotrimazole troche or nystatin) had significantly higher BDG levels compared with those not receiving these medications (*P =* 0.03). Only 4 patients were on systemic antifungals at the time of sampling (Table 1). We did not identify any significant effect of year of sample acquisition, experimental batch, or time of hospital or ICU admission to sample acquisition on measured BDG levels (Supplemental Figures 2 and 3).

### Plasma BDG correlates with host-response biomarkers and the hyperinflammatory subphenotype.

**Figure 3:**
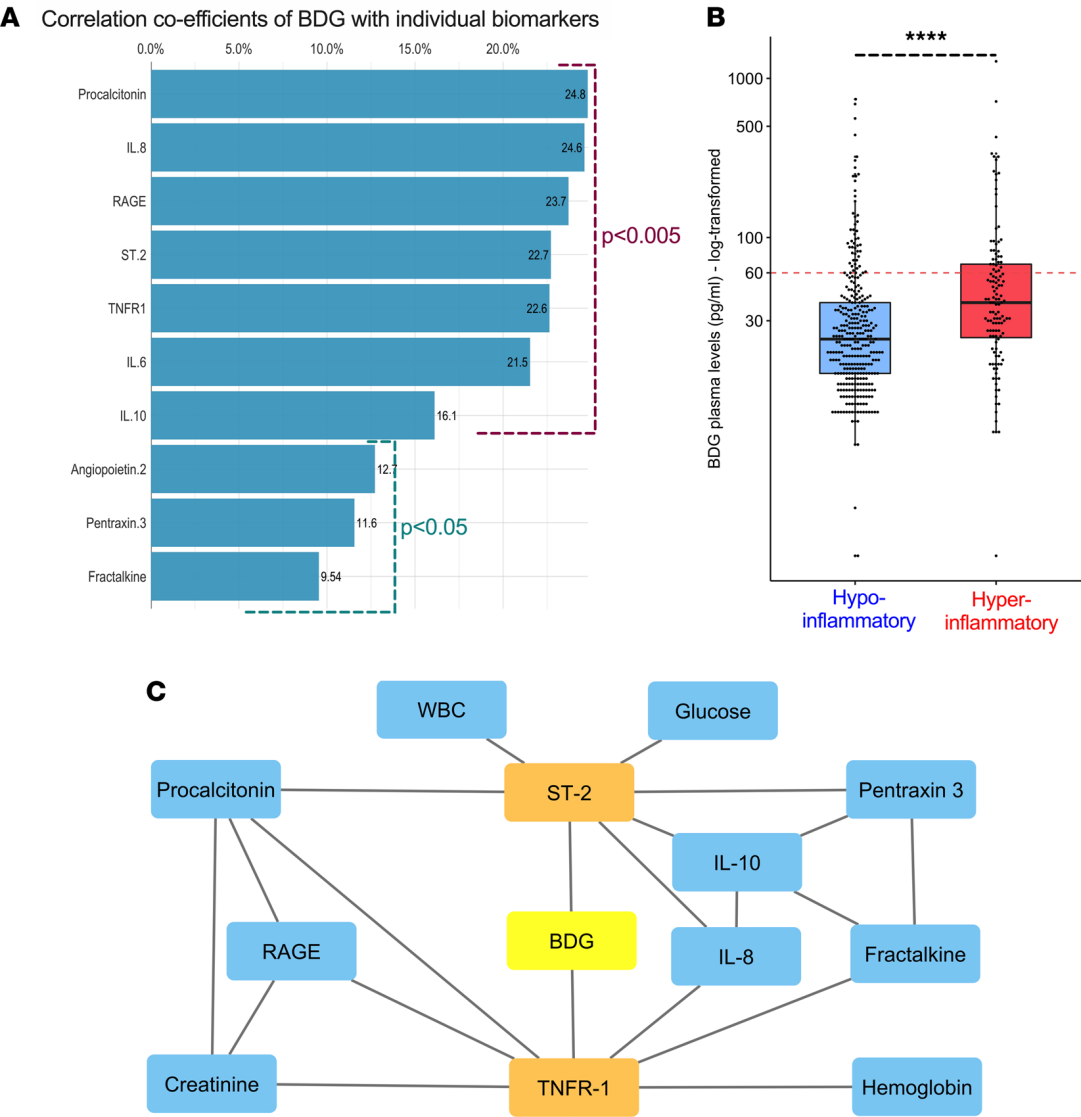
BDG levels are associated with inflammatory host responses. (**A**) Significant correlations of BDG with 10 host-response biomarkers, with the size of each bar corresponding to the correlation coefficient for each biomarker. Seven out of 10 correlations remained significant after adjustment for multiple testing with the Benjamini-Hochberg method (*P <* 0.005 shown in purple). (**B**) Patients assigned to the hyperinflammatory subphenotype had significantly higher BDG levels (****P < 0.0001). (**C**) Probabilistic graphical model analysis demonstrating first and second neighbors of the BDG variable, when considered in conjunction with 32 clinical and biomarker variables. Two first neighbors were identified: TNFR1 and ST-2. Ang-2, angiopoietin-2; ST-2, suppression of tumorigenicity-2; RAGE, receptor for advanced glycation end products.

We systematically profiled host responses by measuring plasma biomarkers in pathways of innate immunity (IL-6, IL-8, IL-10, TNFR1, fractalkine, suppression of tumorigenicity-2 [ST-2]), alveolar epithelial injury (RAGE), endothelial injury (angiopoeitin-2), and response to bacterial infection (procalcitonin and pentraxin-3; ref. 3). BDG levels at baseline positively correlated with levels of all measured biomarkers (*P <* 0.05), with 7 of these correlations remaining significant after adjustments for multiple testing (Figures 3A and Supplemental Figure 4). We synthesized host-response profiles by classifying patients into a hyperinflammatory (28%) versus a hypoinflammatory subphenotype (72%) based on latent class analysis as previously described (3). Hyperinflammatory patients had significantly higher BDG levels compared with hypoinflammatory patients (39 pg/mL [24–68 pg/mL] vs. 23 pg/mL [1–39 pg/mL], *P <* 10^–6^; Figure 3B). BDG levels offered modest discrimination as a single biomarker predictor for subphenotype classification by receiver operating characteristic (ROC) curve analysis (AUC 0.658, Supplemental Figure 5), with an optimal cutoff of 40 pg/mL, which was applied for subsequent analyses. Patients with high BDG levels (≥40 pg/mL, 31%) had significantly higher odds of being classified in the hyperinflammatory subphenotype (OR [95% CI], 2.88 [1.83–4.54], *P <* 0.0001).

### Network analyses reveal association of BDG with key elements of the hyperinflammatory response.

To comprehensively examine causal associations between baseline clinical variables, biomarkers, and BDG, we utilized the probabilistic graphical model (PGM) framework (33, 34). From a total of 32 variables, only 2 were directly linked with BDG (i.e., first neighbors in PGM): TNFR1, a predictive biomarker for the hyperinflammatory subphenotype, and ST-2, the soluble receptor of IL-33 that functions as an alarmin released by respiratory epithelia during acute lung injury (Figure 3C and ref. 35). From these results, we considered TNFR1 and ST-2 as the most representative biomarkers of the hyperinflammatory response associated with BDG.

### Host inflammatory responses are associated with dectin-1 receptor genotypes.

**Figure 4:**
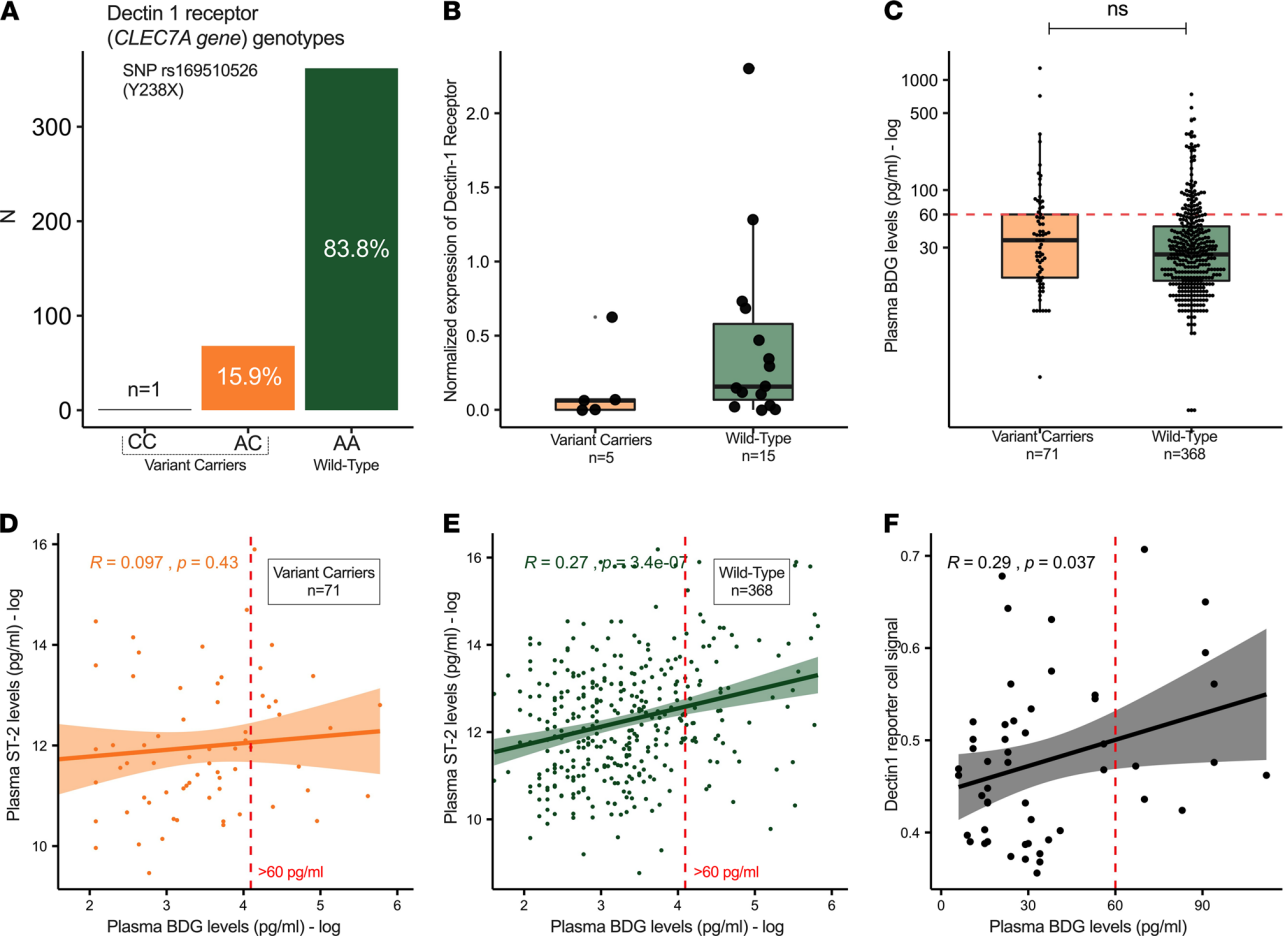
Dectin 1 receptor genotypes and inflammatory response in patients with acute respiratory failure. (**A**) Distribution of genotypes of the SNP rs169510526 (Y238X) of the *CLEC7A* gene in 453 patients with acute respiratory failure. (**B**) Variant carriers had numerically lower (but not statistically significant different) levels of normalized protein levels of the dectin-1 receptor by Western blot on isolated WBCs compared with WT participants (*n =* 20 total WBC samples). (**C**) No significant differences of BDG levels by Y238X genotypes. (**D** and **E**) No significant correlation between BDG levels and ST-2 in variant carriers (**D**) but highly significant correlation in WT participants (**E**). (**F**) Plasma BDG levels significantly correlated with reporter cell signal at 10 hours after stimulation of dectin-1 reporter cells (human embryonic kidney cells that stably overexpress the dectin-1 receptor and contain an NF-κB reporter) with 36 plasma samples containing known concentrations of BDG.

We then interrogated genetic variation of the *CLEC7A* gene that encodes the dectin-1 receptor, which is responsible for BDG recognition on the cell surface of macrophages, DCs, and epithelial cells (20). In 434 patients of the ARF cohort, we genotyped the rs169510526 (Y238X) polymorphism of the *CLEC7A* gene, which results in a premature stop codon and decreased expression of the dectin-1 receptor (20). Sixteen percent of patients were Y238X variant carriers (15.9% heterozygotes and a single homozygote of the minor allele C; Figure 4A). In a subset of available samples (*n =* 20) with WBC pellets, variant carriers (*n =* 5) had numerically (but not statistically significant) lower detectable levels of dectin-1 receptor by Western blot compared with WT patients (*P =* 0.16; Figure 4B and Supplemental Figure 6). There were no differences in circulating BDG levels by genotype (Figure 4C). We then examined the previously detected associations of BDG with ST-2 and TNFR-1 in the context of *CLEC7A* genotypes. TNFR-1 was significantly correlated with BDG in both variant carriers (*n =* 71, *R* = 0.33, *P =* 0.007) and WT patients (*n =* 368, *R* = 0.21; *P <* 0.0001), whereas ST-2 was significantly correlated with BDG levels (*R* = 0.27, *P <* 0.0001) only in WT patients (Figure 4, D and E). This pattern of results suggests that higher levels of ST-2 may be produced in the presence of the PAMP ligand (BDG) and normal expression of the responsible pattern recognition receptor (dectin-1). To further examine for specificity of dectin-1 receptor pathway activation, we stimulated dectin-1 reporter cells (human embryonic kidney cells overexpressing dectin-1 and containing an NF-κB reporter) with 36 plasma samples from patients with ARF. The NF-κB reporter signal correlated with the measured BDG concentrations at both 6 and 10 hours after incubation (Figure 4F), but at the same time with none of the other 10 measured inflammatory biomarkers. Such specificity in the association between BDG levels and NF-κB reporter signal may suggest that it was the BDG molecules present in the plasma that stimulated the dectin-1 receptor in the reporter cells in vitro.

### Potential origin of the circulating BDG in the plasma.

Given the significant associations of BDG with host inflammation, we sought to understand whether plasma BDG originates from either the respiratory and/or the intestinal tract as the 2 main mucosal surfaces with possible fungal colonization during critical illness that were accessible for study by our available biospecimens.

**Figure 5:**
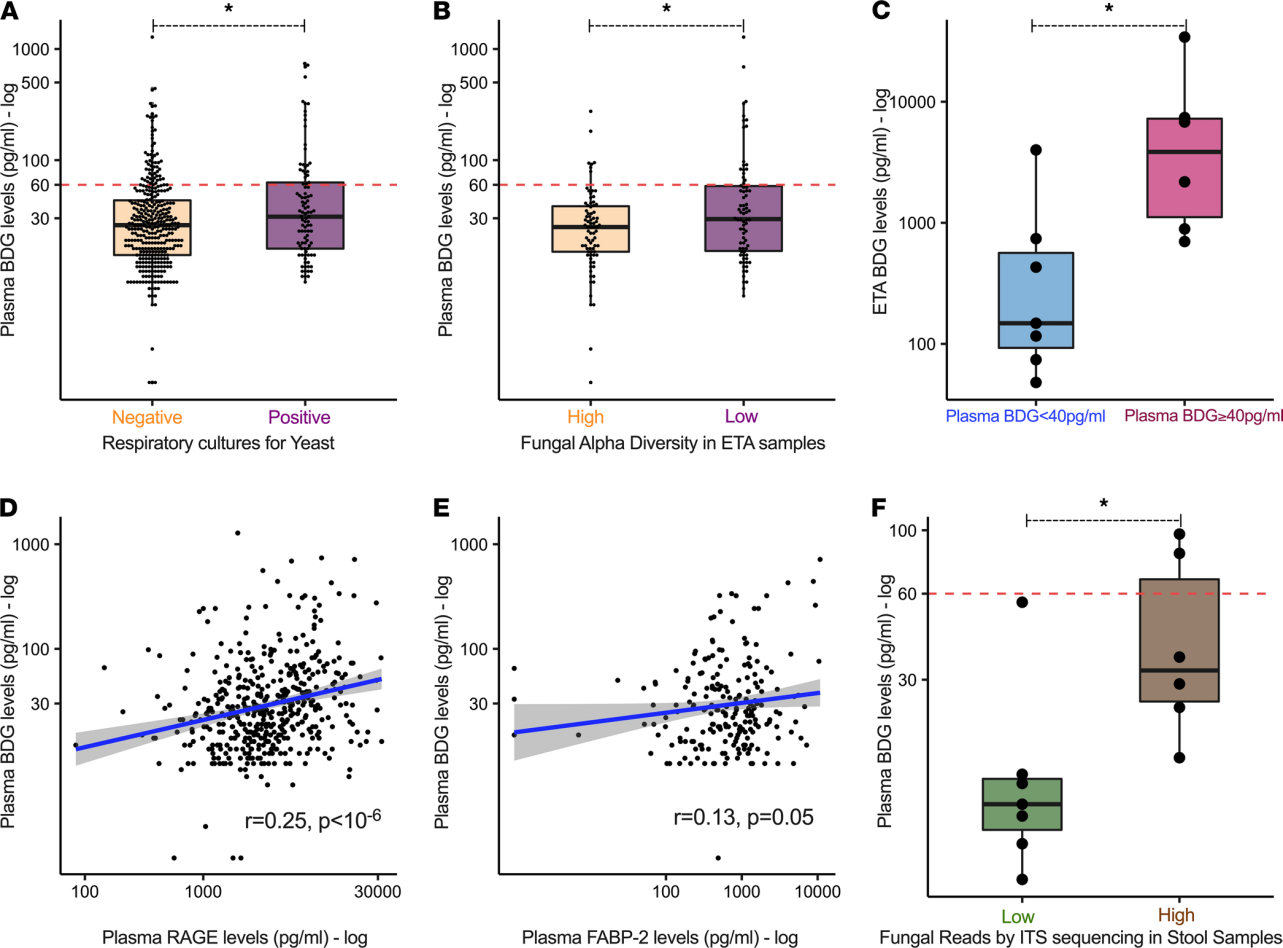
Integrative analyses of culture-based and culture-independent methods for fungal detection and plasma biomarkers of epithelial permeability for potential sources of BDG translocation. (**A**) Patients with respiratory specimen cultures positive for yeast growth (clinically considered as colonization) had higher plasma BDG levels than patients without yeast in respiratory cultures or unavailable culture results. (**B**) Patients with endotracheal sample (ETA) fungal communities with low alpha diversity by sequencing (Shannon index of zero, effectively communities of single fungal species) had higher levels of plasma BDG levels compared with patients with higher alpha diversity. (**C**) Patients with high plasma BDG levels (≥40 pg/mL) had higher levels of BDG measured in matched ETA sample supernatants compared with patients with low plasma BDG (<40 pg/mL). (**D**) Plasma BDG levels were significantly correlated with levels of receptor of advanced glycation end products (RAGE), a marker of alveolar epithelial injury and permeability. (**E**) Plasma BDG levels were significantly correlated with levels of fatty acid binding protein-2 (FABP-2), a marker of intestinal barrier permeability. (**F**) Patients with stool samples with reliably detectable fungal sequences (*n =* 6, denoted as those with high number of fungal reads by internal transcriber spacer [ITS] sequencing) had higher corresponding plasma BDG levels than patients with stool samples with an undetectable or a very low number of fungal reads. *P < 0.05.

For the respiratory tract, we first reviewed clinical microbiologic reports of available culture results from respiratory specimens (endotracheal aspirates [ETA] or bronchoalveolar lavage fluid) for detection of colonizing yeast around the time of research blood sampling. Yeast growth in respiratory specimen cultures (clinically considered as nonpathogenic) was reported for 97 (21%) of patients with ARF, who had higher plasma BDG levels compared with patients with negative or no available cultures (*P =* 0.02; Figure 5A). For culture-independent study of respiratory tract fungi, we extracted DNA from research ETA samples (*n =* 189) and performed internal transcribed spacer (ITS) region sequencing of the fungal genome. Derived sequences (median, 5847, IQR [572–26,818] high-quality fungal reads) were predominantly assigned to *Candida* species (98.3%), including *C*. *albicans* (74.3%), *C*. *dubliniensis* (11.8%), *C*. *tropicalis* (10.1%), and *C*. *parapsilosis* (2.1%) (Supplemental Figure 7A). ETA fungal communities with low alpha diversity (Shannon index of zero, reflecting communities of single *Candida* species — Supplemental Figure 7B) had higher plasma BDG levels than ETA communities with high alpha diversity (Shannon index = 0.58, *P =* 0.05; Figure 5B). By directly measuring BDG in supernatants of a subset of research ETA samples (*n =* 13), we found that patients with high plasma BDG (≥40 pg/mL) had higher levels of BDG in matched ETA samples compared with patients with low plasma BDG (*P =* 0.018; Figure 5C). Beyond measures of airway colonization by fungi, we examined the contribution of lung epithelial injury through measurements of plasma RAGE, which was significantly correlated with all measured inflammatory biomarkers (Supplemental Figure 4A). BDG was significantly associated with RAGE in unadjusted (*P =* 0.005; Figure 5D) and adjusted linear regression models (*P =* 0.014, adjusted for yeast growth in respiratory cultures and alpha diversity by sequencing), suggesting that injured alveoli may contribute to circulating BDG.

To examine the potential origin of BDG from the intestinal tract, we measured plasma levels of fatty acid binding protein-2 (FABP-2), a validated biomarker of intestinal barrier integrity (36) in a random subset of 220 patients. BDG levels were significantly associated with FABP-2 (*P =* 0.05; Figure 5E), an association that remained significant (*P =* 0.03) after adjustment for variables potentially related to intestinal tissue ischemia and fungal intestinal overgrowth (administration of vasopressors and systemic antibiotics at time of sampling, respectively). Unlike the lung permeability biomarker RAGE, FABP-2 levels were not significantly positively correlated with inflammatory biomarkers (Supplemental Figure 4B). From a small subset of available stool samples with ITS sequencing (*n =* 13), we identified that 7 samples had a very low number of fungal reads, whereas the remaining 6 samples with reliably detectable fungal sequences were dominated by *Candida* species (95% of reads) (Supplemental Figure 8). These stool samples with a high number of fungal reads were associated with higher BDG levels in matched plasma samples than stool samples with undetectable or low fungal reads (*P =* 0.003; Figure 5F). Thus, integrative analyses of biomarkers of epithelial disruption and culture-based and culture-independent methods for detection of fungi in the respiratory and intestinal tract suggested that BDG may have translocated to the systemic circulation from either or both compartments.

### Patients with higher BDG levels had worse clinical outcomes.

**Figure 6:**
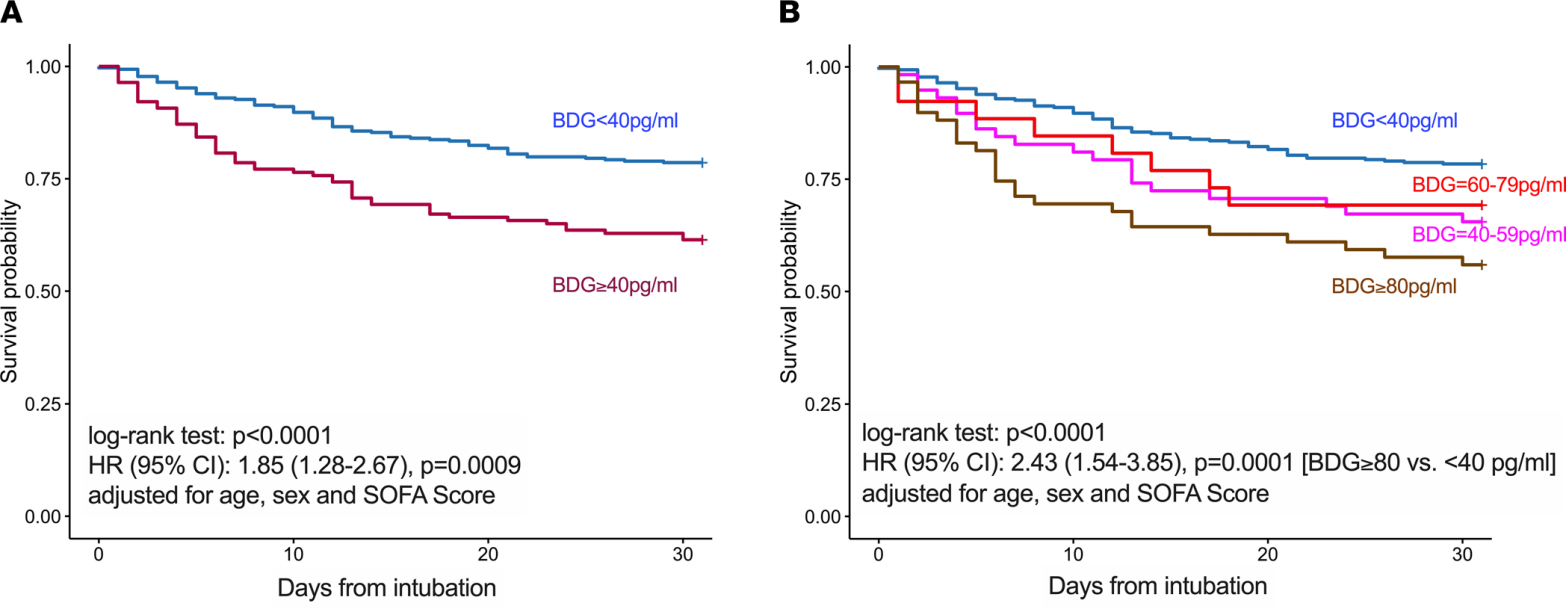
Patients with a high plasma BDG levels (≥40 pg/mL) at baseline had worse 30-day survival after intubation compared with patients with low (<40 pg/mL) BDG levels. (**A**) Kaplan-Meier curve for 30-day survival showing worse outcome after intubation for patients with high BDG levels (≥40 pg/mL) at baseline. HR and 95% CI from a Cox proportional hazards model adjusted for age, sex, and SOFA score at baseline. Further adjustments of the Cox model by markers of epithelial permeability (RAGE and FABP-2) did not affect the significance or strength of the HR for BDG on 30-day survival. (**B**) When patients with BDG of 40 pg/mL or higher were further stratified by standard cutoff points for clinical diagnosis of IFI, we found that BDG of 80 pg/mL or higher (positive test) had significantly worse survival than patients with BDG less than 40 pg/mL (adjusted HR 2.43 [1.54–3.85, *P <* 0.0001), whereas patients with intermediate range BDG levels (40–59 or 60–79 pg/mL) had similar survival between the other 2 groups.

Patients with high BDG levels (≥40 pg/mL) had higher baseline SOFA scores compared with patients with low BDG (<40 pg/mL; *P <* 0.01). Analyzed as a continuous variable, BDG levels were associated with higher incidence of acute kidney injury (AKI), fewer ventilator-free days, and worse 30-day mortality in multivariate regression models, adjusted for other important predictors (i.e., age and SOFA scores) as well as potential confounders of BDG measurement (β-lactam antibiotics and batch of BDG measurement; Table 2). By Cox proportional hazards modeling, BDG levels were significantly associated with worse 30-day survival (adjusted HR: 1.85 [1.28–2.67], *P <* 0.001; Figure 6A). When patients with BDG of 40 pg/mL or higher were further stratified by standard cutoff points for clinical diagnosis of IFI, we found that patients with BDG of 80 pg/mL or higher (positive test) had significantly worse survival than patients with BDG less than 40 pg/mL (adjusted HR, 2.43 [1.54–3.85, *P <* 0.0001; Figure 6B), whereas patients with intermediate range BDG levels (40–59 or 60–79 pg/mL) had similar survival between the other 2 groups. Therefore, these survival analyses revealed a dose-response predictive effect of baseline BDG on 30-day survival, with a predictive threshold above 40 pg/mL.

### Longitudinal evolution of BDG levels and association with outcomes.

**Figure 7:**
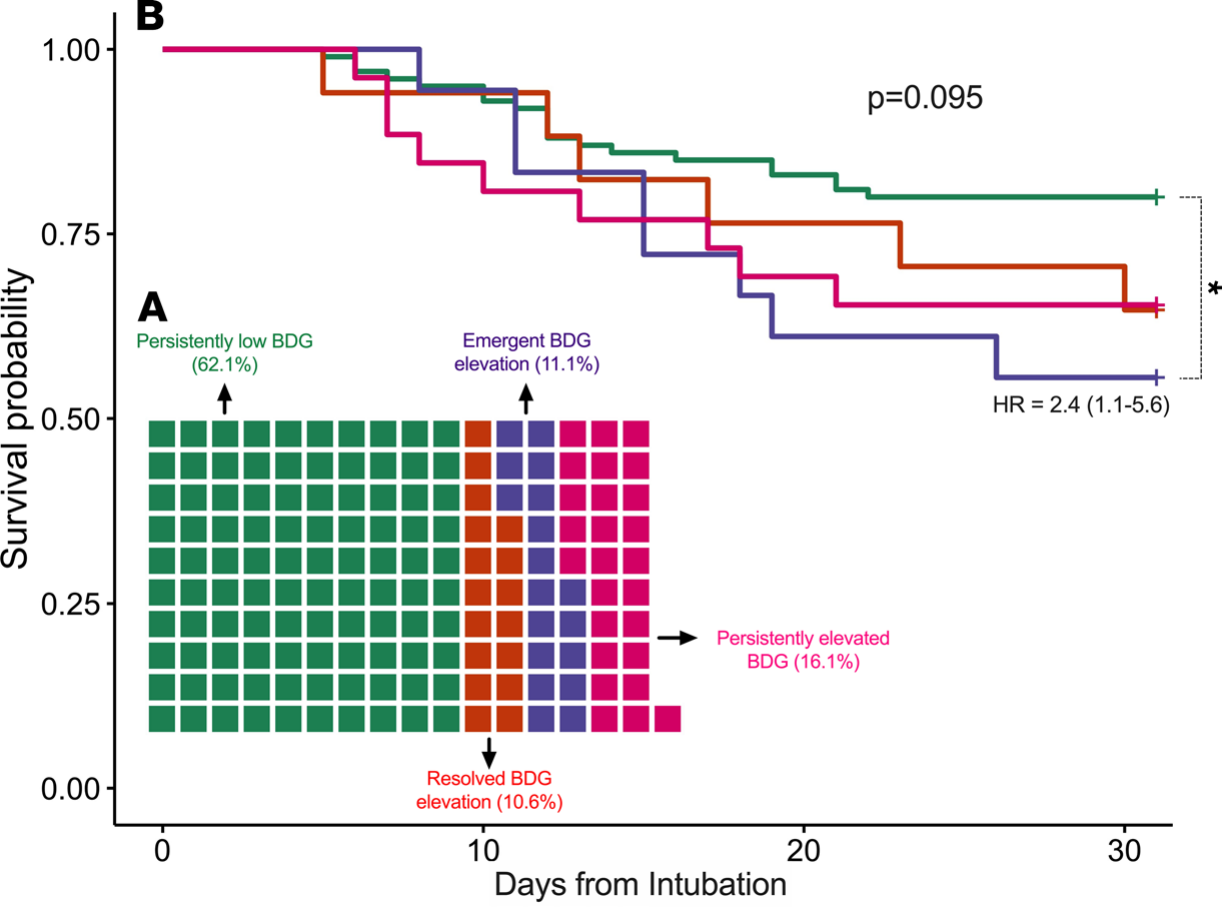
Longitudinal evolution of BDG result from baseline (0–2 days) to middle interval (3–6 days) follow-up samples showed transition from low to high (≥40 pg/mL) levels in 11.1% of patients, who had worse 30-day survival compared with patients with persistently low BDG levels. (**A**) Waffle graph demonstrating the distribution of the 4 groups that emerged by stratifying 156 patients with both baseline and middle interval follow-up samples based on BDG levels (high vs. low) in the 2 intervals: a) persistently elevated (16.1%), b) persistently low BDG levels at both intervals (62.1%), c) high BDG in the baseline interval only (resolved elevation, 10.6%), and d) high BDG in the middle interval only (emergent elevation, 11.1%). (**B**) Kaplan-Meier curves for 30-day survival after intubation in the 4 groups (global *P* value from log-rank test). Patients with emergent BDG elevation had significantly worse 30-day survival compared with patients with persistently low BDG (HR [95% CI] = 2.4 [1.1–5.6]. Because of the small sample size in each group, we did not perform multivariate adjustments in the Cox proportional hazards model.

Among all patients in the ARF cohort with baseline samples (*n =* 453 obtained from days 0–2 after intubation), 156 patients (34%) had follow-up samples in a middle time interval (days 3–6). From baseline and middle interval BDG levels stratified by the cutoff of 40 pg/mL, we classified patients in 4 groups: (a) persistently elevated (16.1%), (b) persistently low BDG levels at both intervals (62.1%), (c) high BDG in the baseline interval only (resolved elevation, 10.6%), and (d) high BDG in the middle interval only (emergent elevation, 11.1%; Figure 7A). Patients with emergent BDG elevation had significantly worse 30-day survival compared with patients with persistently low BDG (HR [95% CI] = 2.4 [1.1–5.6], Figure 7B).

For a smaller subset of patients with ARF who had prolonged ICU stay (>14 days), we measured BDG levels in research samples obtained from 4 time intervals after intubation: baseline (0–2 days, *n =* 113), middle (3–6 days, *n =* 68), late (7–10 days, *n =* 63), and very late (11–14 days, *n =* 58). We observed no significant change of median BDG levels over time, but hyperinflammatory patients had overall higher BDG levels compared with hypoinflammatory patients at follow-up time intervals (Supplemental Figure 9). Four patients developed an incident IFI during their ICU stay (during days 7–13 as defined by positive clinical fungal culture), which was preceded by elevation in BDG compared with the rest of the cohort starting from the middle follow-up interval (*P <* 0.05; Supplemental Figure 10).

### BDG and host inflammation in the validation cohorts.

In the COVID-19 validation cohort (*n =* 97), univariate analyses did not reveal significant correlations between BDG and 10 host-response biomarkers (Supplemental Figure 11A). However, when host biomarkers were synthesized in subphenotypes, hyperinflammatory patients (13%) had higher plasma BDG levels than hypoinflammatory patients (Supplemental Figure 11B; *P =* 0.004). Moreover, integrative analyses of clinical and biomarker variables with PGMs revealed 4 first neighbors of BDG: 2 clinical variables (diabetes and platelets) and the same 2 biomarkers (TNFR1 and ST-2) as in the ARF cohort (Supplemental Figure 12C), offering independent validation for the involvement of BDG in host inflammation.

We observed a markedly different distribution of BDG levels in the TBI cohort (*n =* 137) compared with the nontrauma populations (ARF and COVID-19 cohorts, Supplemental Figure 1). Despite generally higher levels of circulating BDG in the patients with TBI, we found weak correlations between plasma BDG levels and 2 biomarkers of host inflammation: IL-8 measured in research samples and neutrophil/lymphocyte ratio from clinical laboratory results (Supplemental Figure 12A). By PGM analysis, we found that the single variable directly linked with BDG was the injury severity score (Supplemental Figure 12B and refs. 37, 38), a validated prognostic score of injury severity based on the combined effects of injuries in 6 body regions. Therefore, the mechanism mediating high BDG levels appeared to be different in the TBI cohort compared with the nontrauma populations. We then sought to further understand the relationship between severity/location of injury and BDG. We classified patients with TBI based on available component scores of the injury severity score by body region into those with isolated TBI (18%) versus those with concurrent severe extracranial injuries (TBI with polytrauma, 82%). Patients with TBI with polytrauma showed a trend for higher BDG levels (Supplemental Figure 13A) and displayed differential correlations between BDG with neutrophil/lymphocyte ratio and IL-8 compared with patients with isolated TBI (Supplemental Figure 13, B and C). Therefore, BDG levels in a critically ill trauma population may be related to the mechanism of organ/tissue injury. These findings may explain why the robust associations between BDG and host inflammation observed in the medical patient populations (ARF and COVID-19) were not replicated in the TBI cohort.

## Discussion

We demonstrated that mechanically ventilated, medical ICU patients with ARF had higher circulating BDG compared with healthy controls in the absence of IFI at the time of sampling. BDG levels were significantly associated with the adverse hyperinflammatory subphenotype, and this association was validated in an independent cohort of patients with COVID-19. By integrating analyses of culture-dependent and independent methods of fungal colonization as well as biomarkers of epithelial permeability, we did not identify a single primary putative source of translocating BDG but detected significant associations for both the respiratory and intestinal compartments. Baseline BDG levels independently predicted adverse clinical outcomes, whereas serial BDG levels revealed that patients with incident BDG elevation had worse survival than patients with persistently negative BDG testing.

BDG represents an immunostimulatory molecule that is likely to mediate the effects of colonizing fungi in critically ill patients. As a constituent of the cell wall of many potentially pathogenic fungi, BDG is recognized by pattern recognition receptors, such as complement receptor 3 and dectin-1, present on the surface of macrophages, DCs, neutrophils, and pulmonary epithelial cells (20, 39–41). Costimulation of dectin-1 with Toll-like receptors augments NF-κΒ–dependent production of cytokines, such as TNF-α and IL-6 (39, 41). Examination of a common genetic polymorphism (Y238X) that controls dectin-1 receptor expression revealed that WT patients (with predicted normal dectin-1 expression) had higher levels of ST-2 in the presence of high plasma BDG. Importantly, ST-2 was 1 of the 2 biomarkers directly associated with BDG in an agnostic interrogation of both the ARF and COVID-19 cohort datasets using PGM (together with TNFR1). Soluble ST-2 (IL-33 receptor) is involved in dectin-1–dependent stimulation of IL-33 responses by BDG in animal models of fungal colonization (42) and has been strongly associated with worse ventilatory outcomes in patients with ARF (43). Intensified inflammatory responses may be necessary for the recognition and clearance of invading fungi, yet may also lead to exacerbated mucosal inflammation and tissue damage upon extended stimulation.

The role of BDG in the pathogenesis of mucosal injury and inflammation, however, may not be limited to that of a direct stimulant of the innate immune system. Fungal PAMPs in the form of heat-inactivated *Candida* were shown to reduce the killing activity of bone marrow–derived macrophages while enhancing cytokine production (44); intact *Candida* has been shown to reduce the phagocytic abilities of macrophages, potentially by a soluble molecule (45), among other poorly understood mechanisms. As such, fungal PAMPs (including perhaps BDG) may lead to impairment of mucosal immunity and propagation of host injury and inflammation.

Dysregulation of host responses with high levels of circulating inflammatory cytokines and immune cell activation is a key pathogenetic feature in the evolution of critical illness (46). Unsupervised classification analyses in patients with sepsis, severe pneumonia, or ARDS have revealed the presence of distinct subphenotypes, characterized by a hyperinflammatory versus a hypoinflammatory cluster of host responses (3, 4). The markedly worse outcomes and differential treatment responses associated with the hyperinflammatory subphenotype offer a new framework for predictive enrichment and the delivery of targeted efficacious therapies (e.g., antiinflammatory drugs to be given only to patients with the hyperinflammatory subphenotype who are likely to benefit; ref. 6). However, the proximal molecular determinants of ARDS and sepsis subphenotypes have not been defined, whereas clinical stratifications by types of insult (e.g., pneumonia or extrapulmonary infection) have not been informative for distinguishing host responses (3, 47). Thus, we posit that at least some of the observed host-response heterogeneity may be accounted for by host-microbial interactions (10, 48). Beyond the well-recognized importance of bacterial pathogens, translocating fungal PAMPS and their interactions with innate immune cells may also significantly contribute to the evolution and outcome of critical illness.

Epidemiological observations support the role of fungi in critical illness, even outside the context of conventionally defined IFI. Evidence of *Candida* colonization in the airways or fungal overgrowth in the gastrointestinal tract has been associated with worse clinical outcomes, including but not limited to the development of serious bloodstream infections due to systemic invasion of fungi from different mucosal surfaces (15, 17–19, 49). Factors that have been associated with fungal colonization in critically ill patients include widespread antibiotic use, which allow fungi to grow and overtake environmental niches (19), as well as primary or iatrogenic immunosuppression, such as with inhaled corticosteroid therapies leading to upper respiratory tract fungal colonization (50). Our study utilized culture-independent methods (BDG measurement and next-generation sequencing approaches) to examine translocation of fungal elements into the bloodstream, their interactions with innate immunity, and prognostic importance in a broad medical ICU population with no clinical evidence of IFI.

We observed associations of circulating BDG with innate immune responses as well as biomarkers of epithelial permeability. BDG may be stimulating innate immunity not only through mucosal interactions but also in the bloodstream and within the reticuloendothelial system (44). We further demonstrated that levels of systemic inflammatory response (represented by ST-2) varied by dectin-1 receptor genotypes, and that BDG present in these patient samples was also able to stimulate the dectin-1 receptor in vitro in a reporter cell system. Therefore, circulating BDG molecules may not simply represent a marker of mucosal barrier injury that has found its way into the bloodstream, but also an active mediator of inflammatory organ dysfunction and illness severity (9, 44).

Our integrative analyses of permeability biomarkers and culture-dependent and culture-independent assays of fungal colonization did not reveal a specific primary source of circulating BDG from either the lungs or the gut. With regards to the lungs, soluble RAGE, an extensively validated biomarker of ARDS radiographic and clinical severity (3, 24, 51), was significantly associated with plasma BDG levels, potentially reflecting the degree of pulmonary epithelial/endothelial disruption and ability of BDG from airway colonizing fungi to enter the systemic circulation. For the gut, BDG levels were weakly correlated with FABP-2, a biomarker of intestinal barrier disruption (36). The weak correlation of BDG with gut permeability, contrasted with the highly significant association with lung epithelial injury, represented an unexpected pattern of findings. Given the observational nature of our translational study and inherent limitations in reliably sampling gut microbiota, we could not determine the relative contributions of the gut (presumably rich in fungal biomass) versus the lungs (lower biomass). FABP-2 alone may not fully capture the level of intestinal epithelial disruption, and our cohort of patients with ARF on positive pressure mechanical ventilation may be enriched for lung-origin BDG. In animal models of intestinal leakage via direct physical or chemical injury, serum BDG testing showed low sensitivity but high specificity for a permeable gut (30). Direct measurement and study of the phenomenon of microbial translocation in human translational studies is challenging, and no gold-standard method exists for in vivo study (52). Thus, further study is needed to understand the potential sources and mechanisms of translocating BDG, which may then offer a noninvasive and clinically available surrogate measure of gut/lung permeability in critically ill patients, with important prognostic implications.

High BDG levels examined at different thresholds (either ≥40 pg/mL or by the standard diagnostic cutoffs of ≥60 or ≥80 pg/mL) predicted worse outcomes in our inclusive cohort of patients with ARF. Baseline BDG levels were associated with higher severity of illness by SOFA score and predicted incident organ dysfunction (AKI and fewer ventilator-free days) as well as significantly worse 30-day survival. These adverse outcome predictions remained significant after multivariate model adjustments for age and baseline SOFA scores, suggesting that BDG may capture prognostic information not offered by clinically available stratification tools. Similar observations of poor prognosis associated with BDG have been reported in smaller cohorts of patients with suspected VAP as well as in patients with sepsis (22, 30). Our findings in a much larger and inclusive cohort of patients with ARF, as well as the independent validation in patients with COVID-19, suggest that BDG may offer important prognostic information in the course of critical illness. On the other hand, our analyses of patients with TBI revealed a markedly different distribution of BDG values, as well as different probable mechanisms for the elevated BDG levels, especially among patients with extracranial polytrauma. Surgical material packing (e.g., gauze or sponge) may allow for large amounts of BDG to be leached and generate high levels of circulating BDG, which would then be unrelated to IFI or fungal translocation as observed in nontrauma patients (32). We did not have information available on the extent/type of surgical packing in the TBI cohort to directly measure such confounding effects. Nonetheless, our comprehensive analyses in 3 patient cohorts highlighted the importance of careful selection of patient population for BDG study in critical illness. BDG emerged as a promising predictor of host inflammation and outcome in medical critical illness, but such effects were not replicable in traumatic critical illness, necessitating context-specific study and interpretation of BDG levels.

Our study has several limitations. As a single-center study, generalizability of our findings in critically ill populations beyond our tertiary care institution requires external validation in other centers. Our study is also limited by the available sample size. Despite being the largest study of BDG measurement in ARF, results from analyses for subgroups (e.g., longitudinal sample analyses) and specific biomarkers (e.g., FABP-2) require cautious interpretation because the effective sample size for certain analyses was smaller. Although we aimed to control for potential confounders of BDG measurement from available variables (batch of measurement, β-lactam antibiotics, or bacteremia; ref. 32), there may be other sources of BDG false positivity, such as hemodialysis or blood product preparation filter processing, that were not possible to account for in our analyses (53, 54). Finally, the extent of microbiological workup for diagnosis of bacterial infection or IFI was directed by the treating physicians and, as such, microbiological testing was not uniform. Thus, it is possible that some of the cases with high BDG levels could represent clinically undiagnosed or preclinical IFI, as revealed in a small number of patients with incident IFI diagnosis in our longitudinal analyses.

In summary, we demonstrated that circulating BDG was an independent predictor of a hyperinflammatory host-response profile and adverse clinical outcomes in mechanically ventilated medical patients with ARF. We validated that BDG was associated with host inflammation in patients with COVID-19. These findings highlight a potentially underappreciated mechanism of biological heterogeneity in critical illness that involves interactions between translocating fungal PAMPs and innate immunity and point toward complex interactions among the host, fungi, and other microbes at different mucosal interfaces. Our findings call for further mechanistic interrogation in animal studies and independent prospective clinical study to validate the biological relevance and prognostic information of circulating BDG.

## Methods

### Clinical cohorts and sample collection

#### Discovery cohort.

From October 2011 to June 2019, we prospectively enrolled a convenience sample of consecutive adult patients with ARF who were intubated and mechanically ventilated in the medical or cardiac ICUs at UPMC. Exclusion criteria included inability to obtain informed consent, presence of tracheostomy, or mechanical ventilation for more than 72 hours prior to enrollment. From 453 enrolled patients, we collected blood samples for centrifugation and separation of plasma and WBC pellets. We also collected noninvasive biospecimens for study of lower respiratory tract microbiota with ETA, and we analyzed stool samples for study of the intestinal microbiota as previously described (10, 55, 56). From all participants, we collected biospecimens at a baseline interval (0–2 days after intubation), and when possible, we collected follow-up blood samples at a middle interval (3–6 days after intubation, *n =* 156). For the subset of patients with prolonged ICU stay (>14 days, *n =* 113), we also analyzed available blood biospecimens at 4 consecutive time intervals after intubation: baseline (0–2 days, *n =* 113), middle (3–6 days, *n =* 68), late (7–10 days, *n =* 63), and very late (11–14 days, *n =* 58).

#### Validation cohorts.

(a) For the COVID-19 cohort, from April through November 2020, we prospectively enrolled 97 hospitalized patients with confirmed or suspected COVID-19 at the time of enrollment (25, 57). SARS-COV-2 infection was confirmed via nasopharyngeal or lower respiratory tract specimen quantitative PCR. We enrolled 41 mechanically ventilated patients and 56 nonintubated patients with hypoxemia receiving supplemental oxygen or noninvasive mechanical ventilation support (15 from the ICU and 41 from high-acuity inpatient wards). We collected baseline plasma samples after enrollment. (b) For the analysis of the TBI cohort, we utilized stored serum samples from 137 mechanically ventilated patients with TBI (26). These patients had been enrolled in a prospective cohort study of TBI between 2004 and 2011 and were between 16 and 75 years of age with severe TBI (26). Serum samples were collected at a median of 21.6 (12.0–32.4) hours after time of intubation.

#### Healthy controls.

We obtained publicly available data from 5 published studies reporting BDG values in healthy controls (healthy volunteers or blood donors; refs. 27–31). Raw data were obtained either as provided individual data points in each study or after data extraction from published distribution graphs following image digitization. We confirmed that obtained distributions matched exactly the summary statistics (median and IQR) reported in each study.

### Laboratory analyses

Detailed laboratory methods for the molecular assays used are provided in the Supplemental Methods. For all available biospecimens, we measured BDG levels using the commercially available Fungitell limulus amebocyte lysate assay (Associates of Cape Cod Inc.) at the manufacturer’s facility. As a biomarker of intestinal permeability, we measured levels of FABP2 with the Quantikine Human FABP2/I-FABP Immunoassay (R&D Systems). For profiling host responses in the ARF and COVID-19 cohorts, we constructed a custom Luminex multi-analyte panel (R&D Systems) targeting biomarkers associated with ARDS outcomes (RAGE, IL-6, IL-8, IL-10, TNFR1, ST-2, fractalkine, angiopoietin-2, procalcitonin, and pentraxin-3) as previously described (3). For host inflammation in patients with TBI, we used previously generated data on neuroinflammatory markers that had been measured using Luminex bead array assays (MilliporeSigma). These markers included IL-1β, IL-6, IL-8, IL-10, TNF-α, soluble vascular adhesion molecule-1 (sVCAM1), and soluble intracellular adhesion molecule-1 (sICAM1; ref. 26). For patients with TBI, we also utilized clinically available neutrophil/lymphocyte ratio values. From 453 whole blood samples of the ARF cohort, we extracted human genomic DNA and performed genotyping of the Y238X polymorphism (rs16910526) of the *CLEC7A* gene with a TaqMan genotyping method. From 20 WBC pellet samples of the ARF cohort, we measured protein levels of the dectin-1 receptor with Western blot. We also used 20 μL of plasma sample from each of 36 patients with ARF to stimulate dectin-1 receptor reporter cells (InvivoGen). These cells stably overexpress the dectin-1 receptor and an NF-κB reporter linked to secreted alkaline phosphatase (SEAP), so that dectin-1 receptor stimulation by BDG can then be measured by SEAP activity quantification. From ETA and stool samples in the ARF cohort, we extracted microbial DNA directly from samples and sequenced the amplified regions 1 and 2 of the ITS rRNA gene on the Illumina MiSeq platform for fungal community profiling (Supplemental Methods). From a small subset of ETA samples (*n =* 13), we measured BDG in ETA sample supernatants.

### Clinical classifications

A consensus committee reviewed clinical and radiographic data and performed retrospective classifications of the etiology and severity of ARF. We performed classifications without knowledge of biomarker data. We retrospectively classified ARF as ARDS per established criteria (58): at risk for ARDS due to the presence of direct (pneumonia or aspiration) or indirect (e.g., extrapulmonary sepsis or acute pancreatitis) lung injury risk factors (59) but lacking ARDS diagnostic criteria, ARF without risk factors for ARDS, or acute-on-chronic respiratory failure. We recorded clinical microbiologic results of respiratory and blood culture specimens obtained within 48 hours of research sample acquisition. We excluded patients with diagnosis of IFI based on microbiologic evidence and consistent clinical picture (23). We recorded antibiotic prescriptions, including β-lactams associated with false-positive BDG tests, topical antifungals for skin candidiasis or oral thrush, and systemic antifungals as part of empiric antimicrobial regimens. We followed patients prospectively for incidence of IFI, AKI, and ventilator-free days at 30 days and 30-day mortality.

### Statistics

We classified baseline plasma samples based on the standard cutoffs of BDG test positivity for the diagnosis of IFI (i.e., <60 pg/mL negative, 60–79 pg/mL indeterminate, ≥80 pg/mL positive). We log-transformed BDG and biomarker values for use in regression models. From available biomarker and clinical variables, we classified patients into a hyperinflammatory versus hypoinflammatory subphenotype based on an internally derived and validated parsimonious logistic regression model following application of latent class analyses (3, 60). Baseline characteristics and outcomes between groups were compared using Wilcoxon’s tests for continuous variables and Fisher’s exact test for categorical variables. We used linear regression models to examine associations between BDG levels and biomarkers of host responses. Multiple testing adjustments were implemented with the Benjamini-Hochberg method. We examined differences in BDG levels between hyperinflammatory and hypoinflammatory subphenotypes using Wilcoxon’s tests. We examined associations between BDG levels and clinical outcomes with logistic regression models for AKI and 30-day mortality, zero-inflated negative binomial models for ventilator-free days, and Cox proportional hazard models for 30-day survival, adjusted for clinical predictors (e.g., age, sex, and SOFA score or subphenotype classification) as well as potential confounders of BDG measurement (β-lactam antibiotics and batch of BDG measurement). We examined the association between BDG levels and RAGE (marker of alveolar epithelial damage) using a linear regression adjusted for yeast growth in respiratory cultures and alpha diversity by sequencing. For the association between BDG and FABP2 (marker of intestinal permeability), we built a linear regression model adjusted for variables that may affect the integrity of the intestinal epithelium (receipt of vasopressors) and fungal overgrowth in the gut (systemic antibiotics). For fungal microbiota analyses in 189 ETA samples, we measured alpha diversity by Shannon index and summary relative abundance for the top 10 abundant fungal species. For 13 stool samples with sequencing results, we described summary relative abundance for samples with detectable fungal sequences. All statistical analyses were performed with R statistical software version 3.6.0 (61). Statistical significance was defined as *P* less than 0.05.

### Study approval

The University of Pittsburgh IRB approved the studies (STUDY19050099, STUDY20040036, and STUDY20120028), and written informed consent was provided by all participants or their surrogates in accordance with the Declaration of Helsinki.

## Author contributions

GK and DK contributed to conceptualization, methodology, investigation, software, formal analysis, writing the original draft, review and editing, visualization, and supervision. HY contributed to methodology, investigation, review and editing, and visualization. Yonglong Zhang, NB, XL, CS, JV, RG, JE, WB, FS, and Yingze Zhang performed the investigation and review and editing. MF, ML, AW, and BA Methé contributed to methodology, investigation, and review and editing. PB was involved in methodology, investigation, software, review and editing, and visualization. BJ McVerry and AM were involved in conceptualization, methodology, investigation, writing the original draft, review and editing, and supervision.

## Supplementary Material

Supplemental data

Trial reporting checklists

ICMJE disclosure forms

## Figures and Tables

**Table 1:**
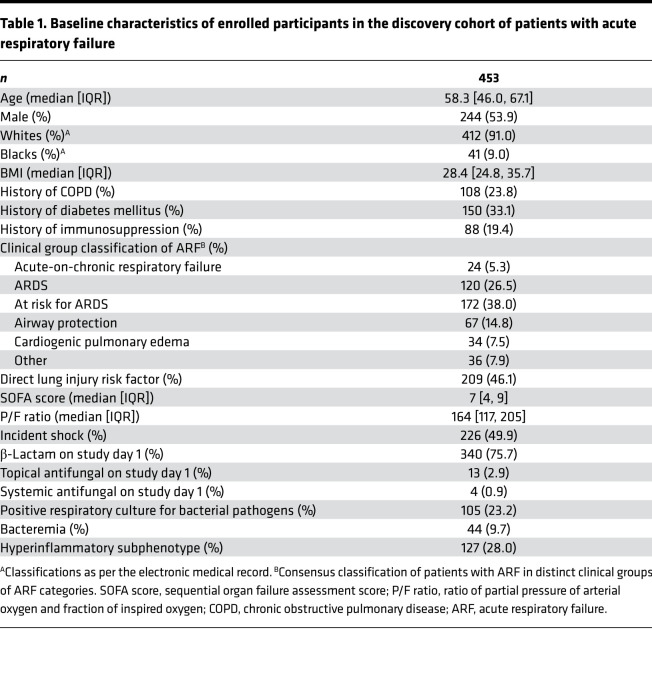
Baseline characteristics of enrolled participants in the discovery cohort of patients with acute respiratory failure

**Table 2:**
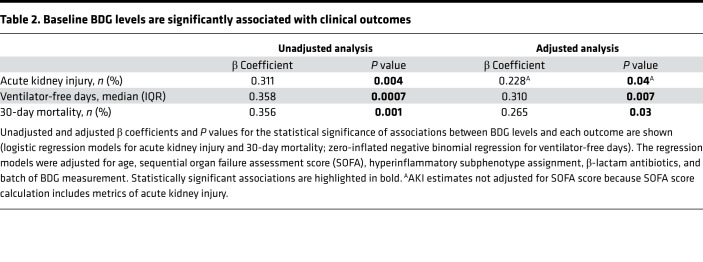
Baseline BDG levels are significantly associated with clinical outcomes

## Version 1 06/15/2021

In-Press Preview

## Version 2 07/22/2021

Electronic publication

## References

[1] Hotchkiss RS, Sherwood ER (2015). Immunology. Getting sepsis therapy right. Science.

[2] Englert JA (2019). Integrating molecular pathogenesis and clinical translation in sepsis-induced acute respiratory distress syndrome. JCI Insight.

[3] Kitsios GD (2019). Host-response subphenotypes offer prognostic enrichment in patients with or at risk for acute respiratory distress syndrome. Crit Care Med.

[4] Sinha P (2020). Development and validation of parsimonious algorithms to classify acute respiratory distress syndrome phenotypes: a secondary analysis of randomised controlled trials. Lancet Respir Med.

[5] Seymour CW (2019). Derivation, validation, and potential treatment implications of novel clinical phenotypes for sepsis. JAMA.

[6] Matthay MA (2017). Clinical trials in acute respiratory distress syndrome: challenges and opportunities. Lancet Respir Med.

[7] Vance RE (2009). Patterns of pathogenesis: discrimination of pathogenic and nonpathogenic microbes by the innate immune system. Cell Host Microbe.

[8] Newton K, Dixit VM (2012). Signaling in innate immunity and inflammation. Cold Spring Harb Perspect Biol.

[9] Amornphimoltham P (2019). Gut leakage of fungal-derived inflammatory mediators: part of a gut-liver-kidney axis in bacterial sepsis. Dig Dis Sci.

[10] Kitsios GD (2020). Respiratory tract dysbiosis is associated with worse outcomes in mechanically ventilated patients. Am J Respir Crit Care Med.

[11] Kitsios GD (2017). Dysbiosis in the intensive care unit: microbiome science coming to the bedside. J Crit Care.

[12] Dickson RP (2020). Lung microbiota predict clinical outcomes in critically ill patients. Am J Respir Crit Care Med.

[13] Watkins RR (2017). Admission to the intensive care unit is associated with changes in the oral mycobiome. J Intensive Care Med.

[14] Tipton L (2017). The lung mycobiome in the next-generation sequencing era. Virulence.

[15] Pendleton KM (2018). Respiratory tract colonization by candida species portends worse outcomes in immunocompromised patients. Clin Pulm Med.

[16] Delisle MS (2011). Impact of Candida species on clinical outcomes in patients with suspected ventilator-associated pneumonia. Can Respir J.

[17] Delisle MS (2008). The clinical significance of Candida colonization of respiratory tract secretions in critically ill patients. J Crit Care.

[18] Samonis G (2005). Levofloxacin and moxifloxacin increase human gut colonization by Candida species. Antimicrob Agents Chemother.

[19] Vardakas KZ (2009). Candidaemia: incidence, risk factors, characteristics and outcomes in immunocompetent critically ill patients. Clin Microbiol Infect.

[20] Calabrese DR (2019). Dectin-1 genetic deficiency predicts chronic lung allograft dysfunction and death. JCI Insight.

[21] Mittal R, Coopersmith CM (2014). Redefining the gut as the motor of critical illness. Trends Mol Med.

[22] Heyland D (2011). Serum β-d-glucan of critically ill patients with suspected ventilator-associated pneumonia: preliminary observations. J Crit Care.

[23] Hage CA (2019). Microbiological laboratory testing in the diagnosis of fungal infections in pulmonary and critical care practice. An official American Thoracic Society clinical practice guideline. Am J Respir Crit Care Med.

[24] Kotok D (2020). The evolution of radiographic edema in ARDS and its association with clinical outcomes: a prospective cohort study in adult patients. J Crit Care.

[25] Bain W COVID-19 versus non-COVID ARDS: comparison of demographics, physiologic parameters, inflammatory biomarkers and clinical outcomes. Ann Am Thorac Soc.

[26] Kumar RG (2015). Acute CSF interleukin-6 trajectories after TBI: associations with neuroinflammation, polytrauma, and outcome. Brain Behav Immun.

[27] Odabasi Z (2004). Beta-D-glucan as a diagnostic adjunct for invasive fungal infections: validation, cutoff development, and performance in patients with acute myelogenous leukemia and myelodysplastic syndrome. Clin Infect Dis.

[28] Pickering JW (2005). Evaluation of a (1→3)-beta-D-glucan assay for diagnosis of invasive fungal infections. J Clin Microbiol.

[29] Esteves F (2014). (1→3)-beta-D-glucan in association with lactate dehydrogenase as biomarkers of Pneumocystis pneumonia (PcP) in HIV-infected patients. Eur J Clin Microbiol Infect Dis.

[30] Leelahavanichkul A (2016). Gastrointestinal leakage detected by serum (1→3)-β-D-glucan in mouse models and a pilot study in patients with sepsis. Shock.

[31] Ramendra R (2020). Cytomegalovirus seropositivity is associated with increased microbial translocation in people living with human immunodeficiency virus and uninfected controls. Clin Infect Dis.

[32] Finkelman MA (2020). Specificity influences in (1→3)-β-d-glucan-supported diagnosis of invasive fungal disease. J Fungi (Basel).

[33] Sedgewick AJ (2019). Mixed graphical models for integrative causal analysis with application to chronic lung disease diagnosis and prognosis. Bioinformatics.

[34] Sedgewick AJ (2016). Learning mixed graphical models with separate sparsity parameters and stability-based model selection. BMC Bioinformatics.

[35] Liu Q (2019). IL-33-mediated IL-13 secretion by ST2+ Tregs controls inflammation after lung injury. JCI Insight.

[36] Shieh A (2020). Gut permeability, inflammation, and bone density across the menopause transition. JCI Insight.

[37] Baker SP (1974). The injury severity score: a method for describing patients with multiple injuries and evaluating emergency care. J Trauma.

[38] Kumar RG (2020). Temporal acute serum estradiol and tumor necrosis factor-α associations and risk of death after severe traumatic brain injury. J Neurotrauma.

[39] Schorey JS, Lawrence C (2008). The pattern recognition receptor dectin-1: from fungi to mycobacteria. Curr Drug Targets.

[40] Camilli G (2018). The complexity of fungal β-glucan in health and disease: effects on the mononuclear phagocyte system. Front immunol.

[41] Brown GD (2002). Dectin-1 is a major beta-glucan receptor on macrophages. J Exp Med.

[42] Lilly LM (2012). The β-glucan receptor dectin-1 promotes lung immunopathology during fungal allergy via IL-22. J Immunol.

[43] Alladina J (2021). Plasma soluble suppression of tumorigenicity-2 associates with ventilator liberation in acute hypoxemic respiratory failure. Am J Respir Crit Care Med.

[44] Panpetch W (2018). Gastrointestinal colonization of Candida albicans increases serum (1→3)-β-D-glucan, without Candidemia, and worsens cecal ligation and puncture sepsis in murine model. Shock.

[45] Collette JR (2014). Candida albicans suppresses nitric oxide generation from macrophages via a secreted molecule. PLoS One.

[46] Meyer NJ, Calfee CS (2017). Novel translational approaches to the search for precision therapies for acute respiratory distress syndrome. Lancet Respir Med.

[47] Calfee CS (2014). Subphenotypes in acute respiratory distress syndrome: latent class analysis of data from two randomised controlled trials. Lancet Respir Med.

[48] Ali F, Sweeney DA (2020). In pursuit of microbiome-based therapies for acute respiratory failure. Am J Respir Crit Care Med.

[49] Zhai B (2020). High-resolution mycobiota analysis reveals dynamic intestinal translocation preceding invasive candidiasis. Nat Med.

[50] Rachelefsky GS (2007). Impact of inhaled corticosteroid-induced oropharyngeal adverse events: results from a meta-analysis. Ann Allergy Asthma Immunol.

[51] Mrozek S (2016). Elevated plasma levels of sRAGE are associated with nonfocal CT-based lung imaging in patients with ARDS: a prospective multicenter study. Chest.

[52] Deitch EA (2012). Gut-origin sepsis: evolution of a concept. Surgeon.

[53] Nagasawa K (2003). Experimental proof of contamination of blood components by (1→3)-beta-D-glucan caused by filtration with cellulose filters in the manufacturing process. J Artif Organs.

[54] Kanda H (2001). Influence of various hemodialysis membranes on the plasma (1→3)-beta-D-glucan level. Kidney Int.

[55] Kitsios GD (2018). Respiratory microbiome profiling for etiologic diagnosis of pneumonia in mechanically ventilated patients. Front Microbiol.

[56] Fair K (2019). Rectal swabs from critically ill patients provide discordant representations of the gut microbiome compared to stool samples. mSphere.

[57] Kitsios GD (2021). Plasma microbial cell-free DNA load is associated with mortality in patients with COVID-19. Respir Res.

[58] ARDS Definition Task Force (2012). Acute respiratory distress syndrome: the Berlin Definition. JAMA.

[59] Neto AS (2016). Epidemiological characteristics, practice of ventilation, and clinical outcome in patients at risk of acute respiratory distress syndrome in intensive care units from 16 countries (PRoVENT): an international, multicentre, prospective study. Lancet Respir Med.

[60] Drohan CM, et al. Host-response subphenotypic classification with a parsimonious model offers prognostic information in patients with acute respiratory failure: a prospective cohort study [preprint]. 10.21203/rs.3.rs-57907/v1 Posted on Research Square August 17, 2020

[61] R Foundation for Statistical Computing RCT. *R: A Language and Environment for Statistical Computing*. CRAN; 2016.

