# Supplementary material for: Plasma 1,3-β-d-glucan levels predict adverse clinical outcomes in critical illness
Source: JCI Insight. 2021 Jul 22;6(14):e141277. doi: 10.1172/jci.insight.141277 (PMC8410081; doi:10.1172/jci.insight.141277)
Supplement: Supplemental data [file jciinsight-6-141277-s082.pdf]

Supplement

Plasma 1,3-beta-D-glucan levels are associated with host inflammatory responses and predict adverse clinical outcomes in critical illness.

1. Extended Methods
2. Supplemental Figures and Tables
3. Supplemental References

Extended Methods:

Western blot of rectin-1 receptor protein expression in white blood cell pellets: We collected blood specimens in Vacutainer collection tubes with sodium citrate as additive and processed samples within 60 min of blood draw. We centrifuged samples at 800xg for 15min and separated plasma from the pellet. We lysed red blood cells (RBC) using RBC lysis buffer according to the manufacture's instruction (Qiagen). We re-suspended white blood cell (WBC) pellets in cold PBS and centrifuged at 800xg for 10 min. We stored the WBC pellet at -80°C immediately for future use. We prepared whole-cell lysates by radioimmunoprecipitation assay buffer (cat# sc-24948 Santa Cruz Biotechnology) with protease inhibitors added freshly. We mixed cell lysates with 4x SDS loading buffer and boiled for 10 minutes. We loaded a total of 15ug of protein for each sample. We separated proteins by 10% sodium dodecyl sulfate polyacrylamide gel electrophoresis, transferred to polyvinylidene difluoride membrane, and blocked in 5% milk for 1 hour at room temperature. We incubated blots with primary antibodies [Dectin-1 (E1X3Z) Cell Signaling#60128] overnight at 4°C, GAPDH [Abcam# ab181602] 1 hour at room temperature and with appropriate secondary antibody (Anti-rabbit IgG, HRP-linked Antibody Cell Signaling#7074) for 1 hour at room temperature. We developed blots using HRP-substrate (Millipore; Billerica, MA) and imaged using Amersham Imager 680 (GE Health). We performed densitometry with the ImageJ software (National Institutes of Health). We arranged samples from left to right by ascending values of plasma BDG levels.

Genotyping of CLEC7A rs16910526 polymorphism: We isolated genomic DNA from whole blood using the Puregene blood DNA isolation kit (QIAgen). We genotyped the single nucleotide polymorphism rs16910526 of the CLEC7A gene using Taqman genotyping method (assay C__33748481_10) and QuantStudio^{TM}5 DNA thermal cycler (ThermoFisher). We perfomed genotyping calls using the QuantStudio^{TM}5 analysis software.

Dectin-1 reporter cells: We used human embryonic kidney (HEK) blue dectin-1 reporter cells (Invivogen) to measure the stimulation of the dectin-1 receptor by BDG (of known concentrations) in 36 plasma samples from patients in the acute respiratory failure cohort. Dectin-1 is the primary receptor that senses BDG with downstream initiation of inflammatory signaling pathways. Dectin-1 reporter cells stably over-express the dectin-1 receptor and a nuclear factor kappa B (NF-κB) reporter linked to secreted alkaline phosphatase (SEAP). Thus, dectin-1 activation by BDG can be quantified via SEAP activity. We plated cells in 96 well plates at a density of ~30,000 cells per well and incubated overnight. We treated cells with treated with either vehicle control, BDG (0.1 mg/mL), or 20 μL of patient plasma (in duplicate). We measured SEAP levels with colorimetry at 650 nm at 6, 10 and 24hr after treatment.

Fungal DNA sequencing: We extracted microbial DNA directly from endotracheal aspirate (ETA) and stool samples using the Powersoil (MoBio) extraction kit following the manufacturer's instructions, as previously described (1–3). Due to the high viscosity of ETA samples, we pretreated them with Dithiothreitol (0.1% DTT in phosphate-buffered saline) in 1:1 dilution to dissolve the mucus and allow usability in DNA extraction columns. We amplified extracted DNA by PCR using the Q5 HS High-Fidelity polymerase (NEB) targeting the internal transcribed regions 1 and 2 of the ITS rRNA gene (4, 5). We utilized reagent controls for each step of the process (DNA extraction and PCR amplification). We amplified four microliters per reaction of each sample with a single barcode in triplicate 25 microliter reactions. We utilized a 2-step nested PCR protocol. Initial cycle conditions were 98°C for 30 seconds, 15 cycles of 98°C for 10 seconds, 55°C for 30 seconds, and 72°C for 90 seconds followed by 72°C for 2 minutes. 5 microliters of the initial PCR reaction were added to the nested cycle and were processed using the following conditions: 98°C for 30 seconds, 15 cycles of 98°C for 10 seconds, 55°C for 30 seconds, and 72°C for 1 minute followed by 72°C for 2 minutes. We combined triplicates and purified with the

AMPure XP beads (Beckman) at a 0.7:1 ratio (beads:DNA) to remove primer-dimers. We performed sample pooling on ice by combining 30 microliters of each sample. We purified the sample pool with the MinElute PCR purification kit. The final sample pool underwent two more purifications – AMPure XP beads to 0.7:1 to remove all traces of primer dimers and a final cleanup using the Purelink PCR Purification Kit (Life Technologies). We quantitated the purified pool in triplicate on the Qubit fluorimeter prior to preparing for sequencing. The sequencing pool was prepared according to instructions by Illumina (San Diego, CA), with an added incubation at 95˚C for 2 minutes immediately following the initial dilution to 20 picomolar. We then diluted the sequencing pool to a final concentration of 7 pM + 15% PhiX control. Amplicons were sequenced on the Illumina Miseq platform. We considered three types of experimental negative control samples used to assess for possible contamination events from sample collection to DNA extraction and PCR amplification: endotracheal aspirate collection controls, DNA extraction, and PCR amplification negative controls. ETA controls consisted of left-over sterile saline not used for sample collection as a negative control to identify potential contamination of the ETA sample from microbial populations present in clinically sterile saline syringes. We included these ETA controls in DNA extraction, PCR amplification, and sequencing alongside clinical samples. DNA extraction negative controls were comprised of sterile water in one DNA extraction column per batch of clinical samples undergoing DNA extraction. We added sterile water in the place of template DNA in our PCR reaction mix as negative PCR amplification controls. We also included PCR amplification positive controls to confirm effective amplification. We used the ZymoBIOMICS Microbial Community DNA Standard (Zymo Research, Irvine, CA), a mock microbial community consisting of genomic DNA of eight bacterial strains and 2 fungal strains as our PCR amplification positive controls. ITS rRNA sequences from the pooled sequencing run were demultiplexed into individual sample/replicate fastq files. The variable-length reads were processed, trimmed and quality filtered with a quality control pipeline utilizing the DADA2 package in R (6). Paired sequences with forward and reverse reads passing the quality filtering and trimming steps were

merged. Chimeras were removed and the Unite database will be utilized to classify reads into amplicon sequence variants (ASVs) using the naïve Bayesian classifier method (7). ASVs are inferred based on sequence data and can be defined at different levels of resolution (phylum, class, order, family, genus, and species). Results of ASV annotation were converted into per-sample taxonomic profiles represented as categorical counts in matrices of dimension (number of samples) x number of categories). The taxa table was filtered for low abundance taxa (relative abundance, <0.005%) and singletons, and also for samples that generated fewer than 10 reads in the range of negative control samples.

BDG measurement in ETA samples: From thawed aliquots of raw frozen ETA samples, we performed heating to 80°C for 15 minutes and then centrifuged at 15,000xg for 10 min. We then performed BDG measurement in supernatants with the Fungitell test (8).

Figure S1: Density plots of BDG concentrations in the discovery cohort of patients with acute respiratory failure and the validation cohorts of patients with COVID-19 and traumatic brain injury (TBI). The conventional cut-off of 60pg/ml of BDG (threshold used to define an indeterminate or positive test in the work up of invasive fungal infections) is highlighted with a vertical dashed red line. The pattern of these density plots reveals that the TBI cohort population had a markedly different distribution of BDG levels compared to the acute respiratory failure and COVID-19 in cohorts.

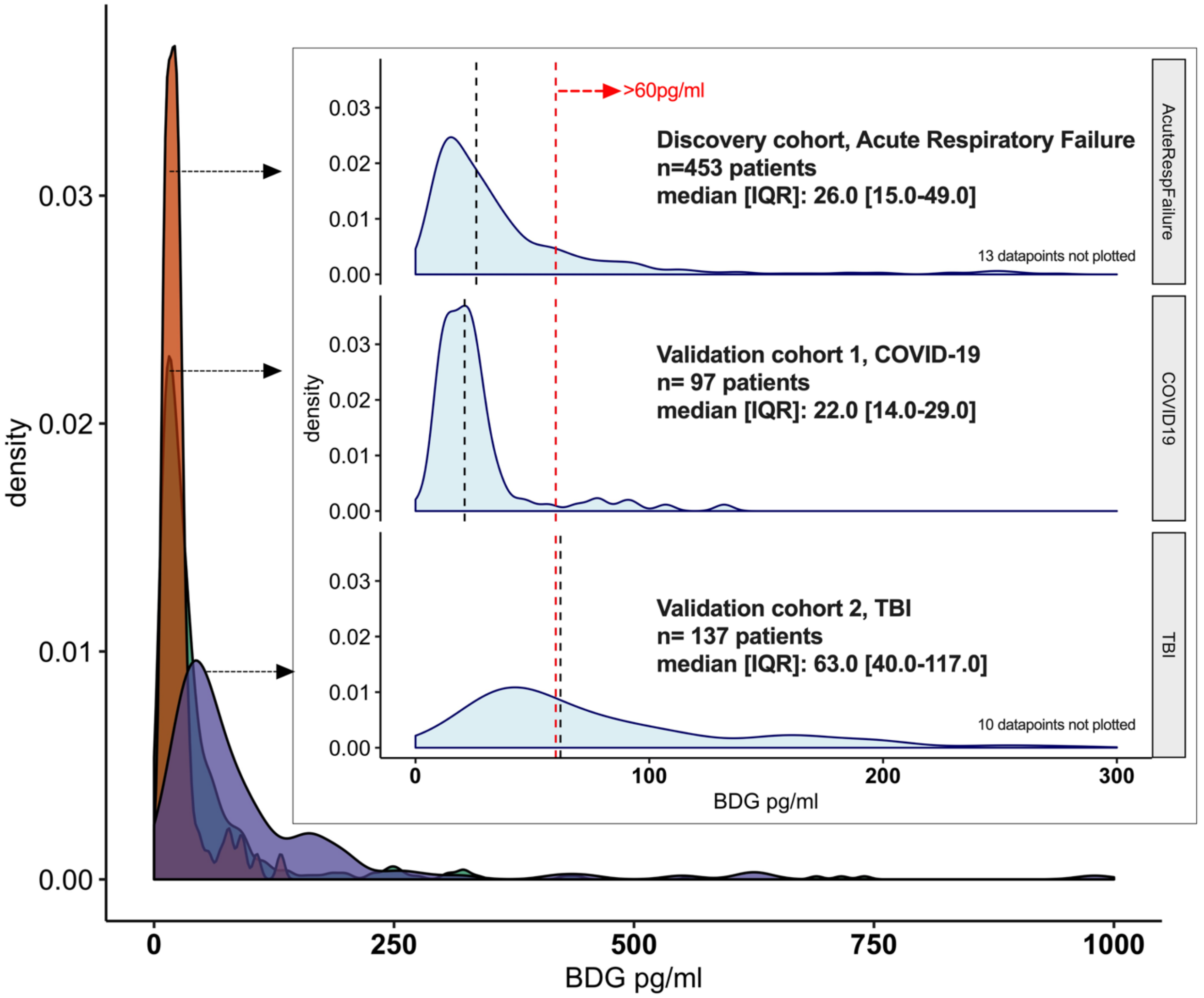

Figure S2: No significant effect of year of enrollment or experimental batch in the levels of measured biomarkers and BDG in the acute respiratory failure cohort. Log-transformed concentrations of plasma TNFR1, ST-2 and BDG are shown on the Y axis, year of enrollment is shown on the X axis, and different experimental batches are depicted by different colors.

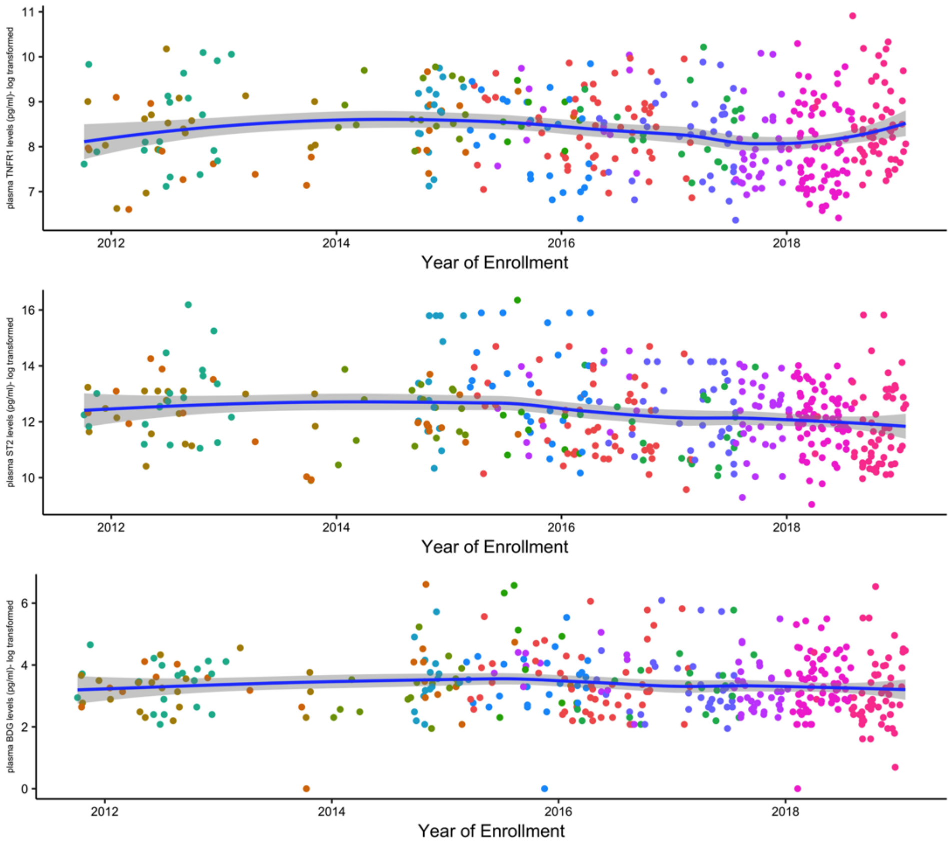

A. TNFR1 levels by year and experimental batch

B. ST2 levels by year and experimental batch

C. BDG levels by year and experimental batch

Figure S3: No significant effect of time from hospital admission (A), or time from intensive care unit admission (B) to sample collection on measured BDG levels of baseline samples (obtained 0-2 days post-intubation).

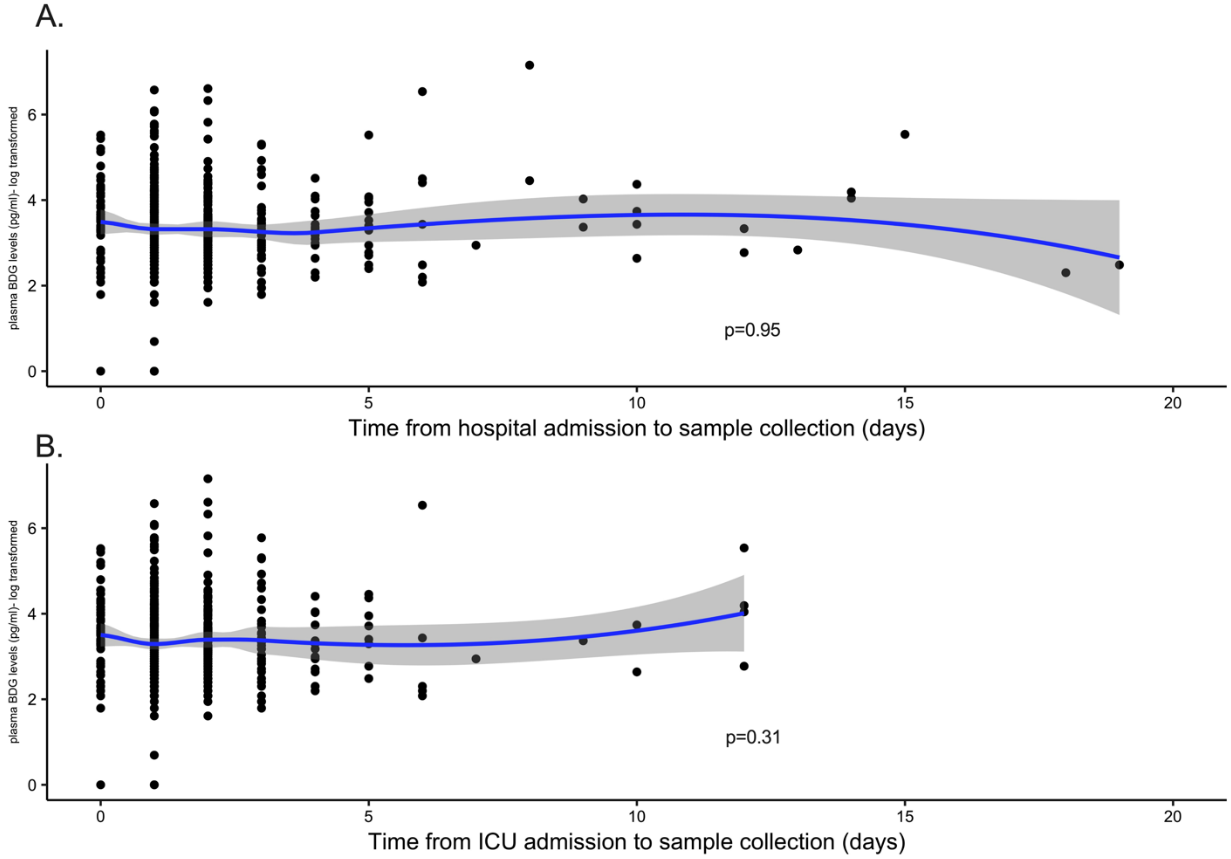

Figure S4: Correlograms depicting direction and statistical significance of correlations between BDG and plasma biomarkers of inflammation and permeability, following adjustment for multiple comparisons with the Benjamini-Hochberg method. Positive correlations are shown in red and negative correlations in blue. Nonsignificant results are marked with an X. A. In 453 patients with acute respiratory failure, BDG was significantly correlated with 7/10 biomarkers. The marker of lung epithelial injury Receptor of Advanced Glycation End-products was significantly correlated with all other inflammatory biomarkers. B. In a random subset of 220 patient samples with available measurements of fatty acid binding protein 2 (FABP-2), a validated biomarker of intestinal epithelial permeability, weak negative correlations were seen for Angiopoietin-2 and Fractalkine.

A. Acute Respiratory Failure - n=453 patient samples

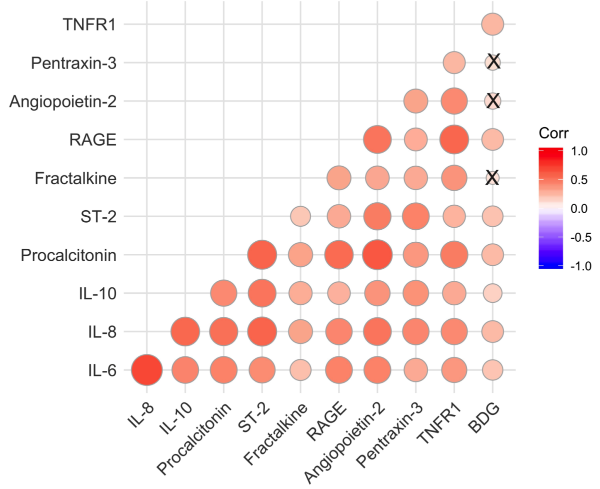

B. Acute Respiratory Failure - n=220 patient samples with available FABP2 measurements

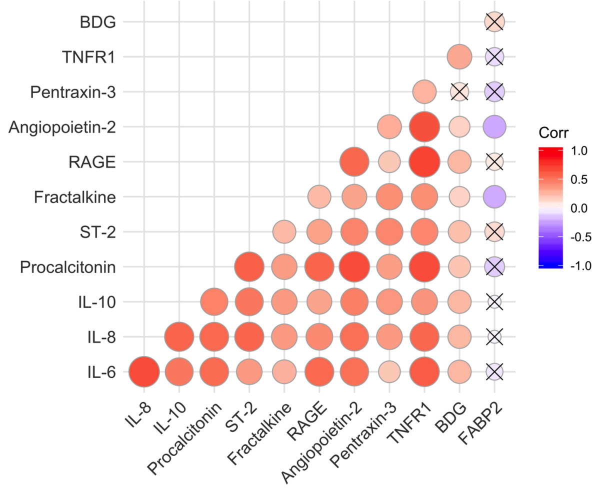

Figure S5: With a receiver operating characteristic (ROC) curve analysis, BDG levels offered modest discrimination for the prediction of a hyperinflammatory subphenotype assignment (area under the curve [AUC] of 0.658). We considered specificity (i.e. avoidance of false positives in the prediction of subphenotypes) as clinically more relevant than sensitivity in the context of phenotype-specific therapeutics. By setting a minimum accepted value of specificity of 0.75, we then derived an optimal cut-off of BDG levels at 40 pg/ml.

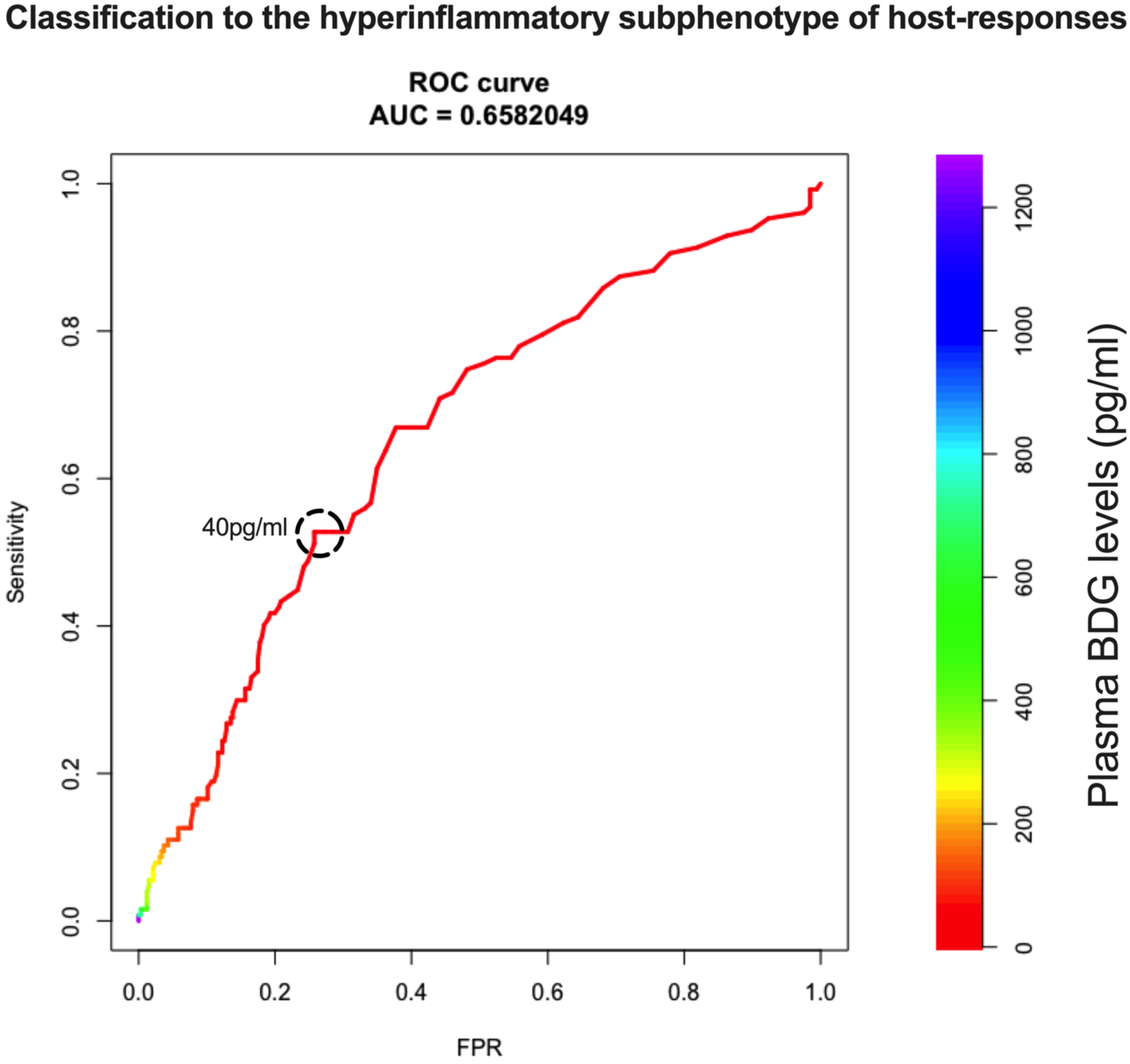

Figure S6: Western Blot images of dectin-1 receptor protein levels in white blood cell lysates from 20 patients' samples in the acute respiratory failure cohort. Dectin-1 receptor levels were normalized against the housekeeping protein GAPDH.

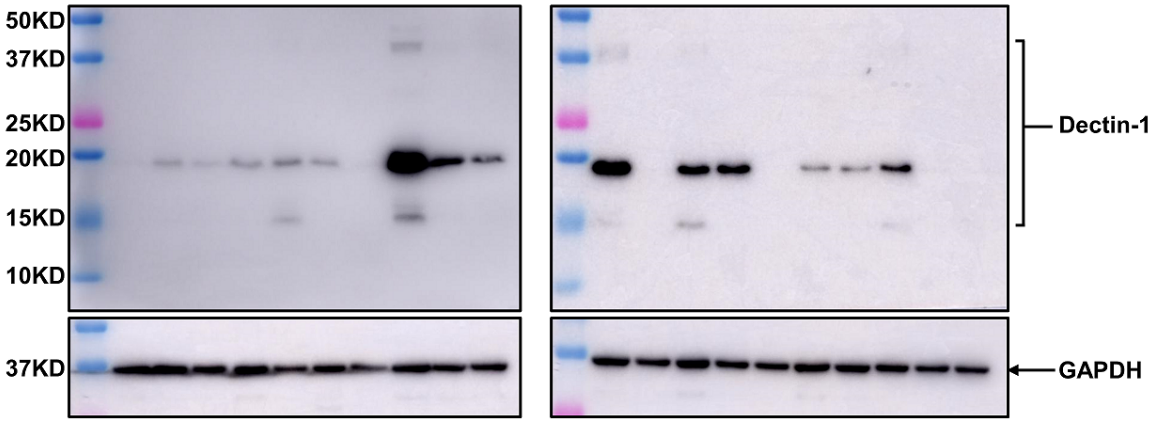

Figure S7. Fungal DNA sequencing in endotracheal aspirates from patients with acute respiratory failure. We performed fungal DNA sequencing by PCR amplification and Illumina sequencing of the internal transcribed spacer (ITS) region of the fungal genome. A. Bar graph of the summary relative abundance for the top 10 most abundant fungal species in 189 samples. Derived sequences were predominantly assigned to *Candida* species (>98%). B. Alpha diversity by Shannon index of fungal communities in ETA samples. We noted a pattern of communities with either Shannon index of zero, effectively consisting of a single *Candida* species, versus communities with non-zero Shannon index (termed as high alpha diversity) that consisted of more than one fungal species.

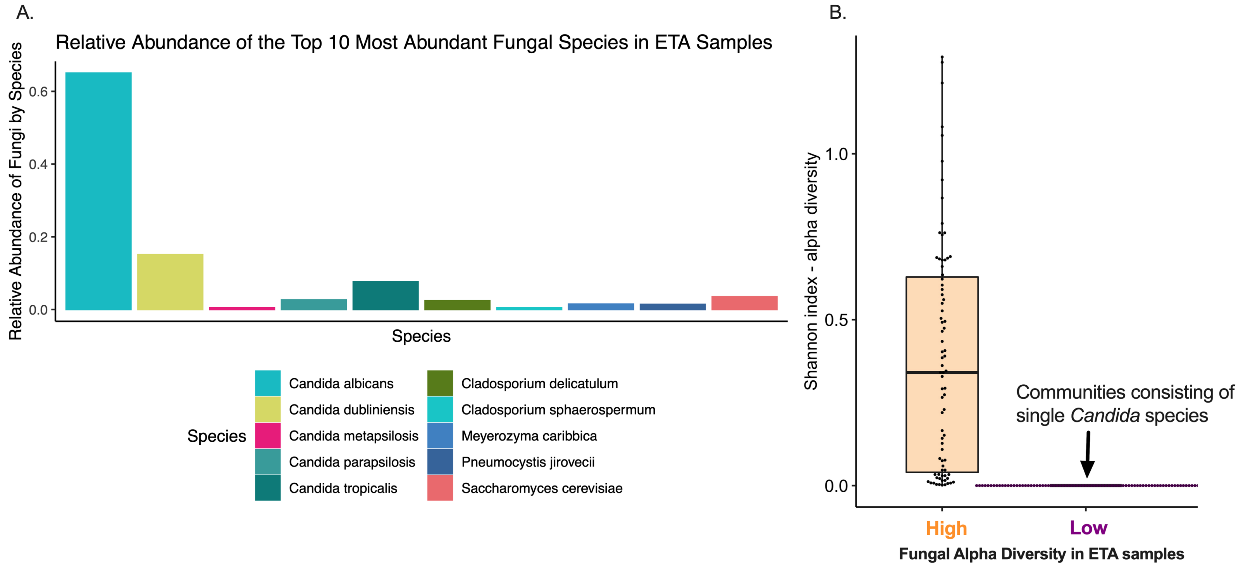

Figure S8. Fungal DNA sequencing results in stool samples from patients with acute respiratory failure. We performed fungal DNA sequencing by PCR amplification and Illumina sequencing of the internal transcribed spacer (ITS) region of the fungal genome. A. Bar graph of the summary relative abundance for the top 10 most abundant fungal species in 13 samples. Derived sequences were predominantly assigned to *Candida* species (>95%). B. Number of high-quality fungal reads by ITS sequencing. Six samples had reliably detectable fungal sequences (termed as those with high number of fungal reads above the median of 5,918 reads) vs. seven samples that had very low or effectively undetectable fungal sequences.

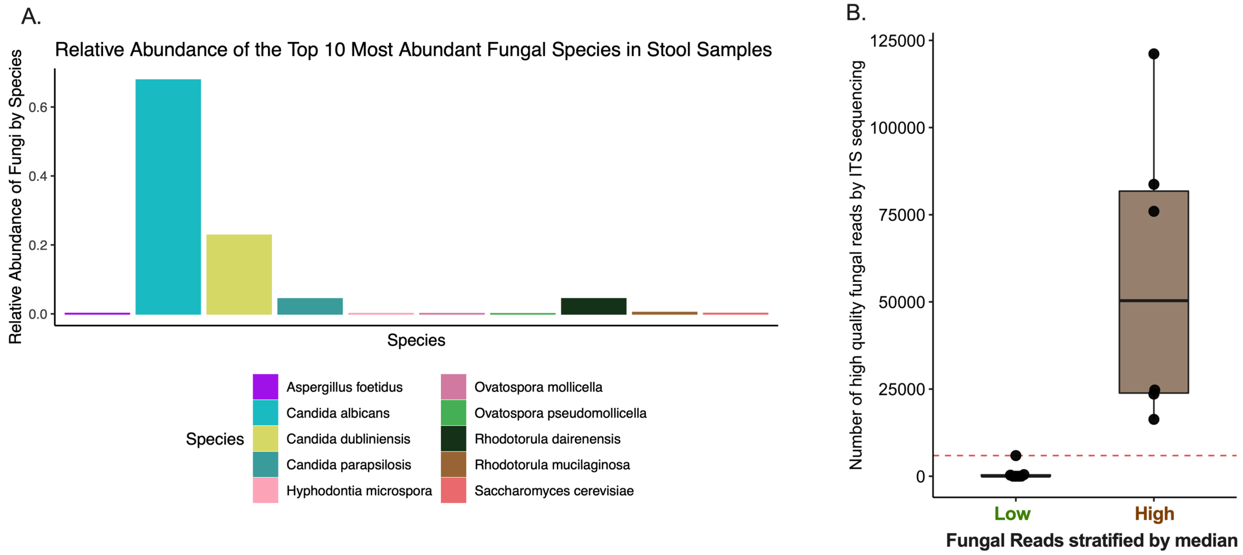

Figure S9: Longitudinal follow-up BDG levels for patients with acute respiratory failure and prolonged ICU stay (>14 days), stratified by host-response subphenotypes. We measured BDG levels in research samples obtained in the following four time intervals post-intubation: baseline (0-2 days, n=113), middle (3-6 days, n=68), late (7-10 days, n=63), very late (11-14 days, n=58). We observed no significant change of median BDG levels over time, but hyperinflammatory patients had overall higher BDG levels compared to hypoinflammatory at the follow-up time intervals (p-values from Wilcoxon tests).

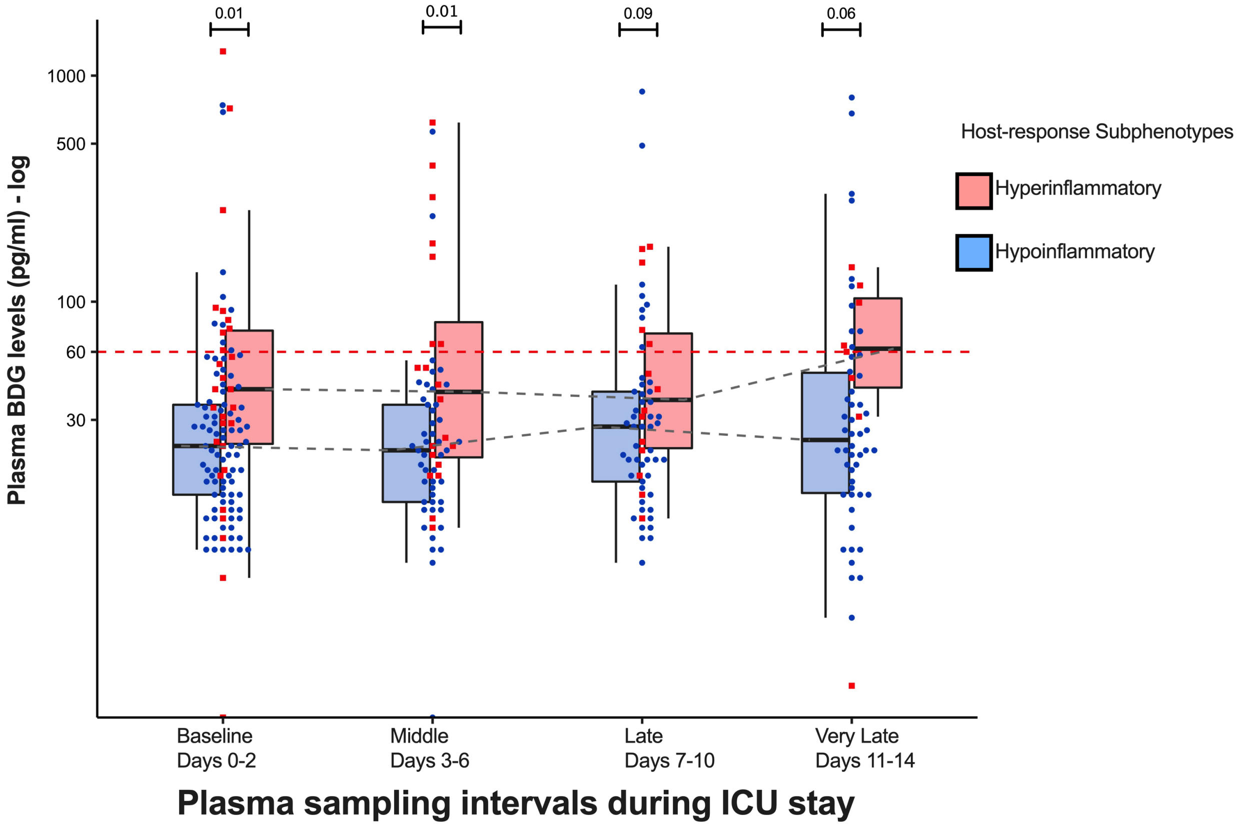

Figure S10: Four patients who developed incident invasive fungal infection (IFI) during the follow-up period in the ICU (up to 14 days) demonstrated higher BDG levels prior to the clinical diagnosis of IFI compared to patients without IFI diagnosis. Each of the four patients with IFI is depicted with a different symbol (whereas the patients without IFI are shown with open gray circles), and timing of clinical sample acquisition for microbiologic cultures that eventually established the IFI diagnosis is shown at the top of the Figure for each patient (samples obtained between days 7-13). Two patients were diagnosed with Pulmonary Aspergillosis, one with Candidemia, and one patient with disseminated Candidiasis (*C. albicans* growth from multiple abscesses). Comparisons of BDG levels at each time interval were performed with Wilcoxon tests. *, p<0.05.

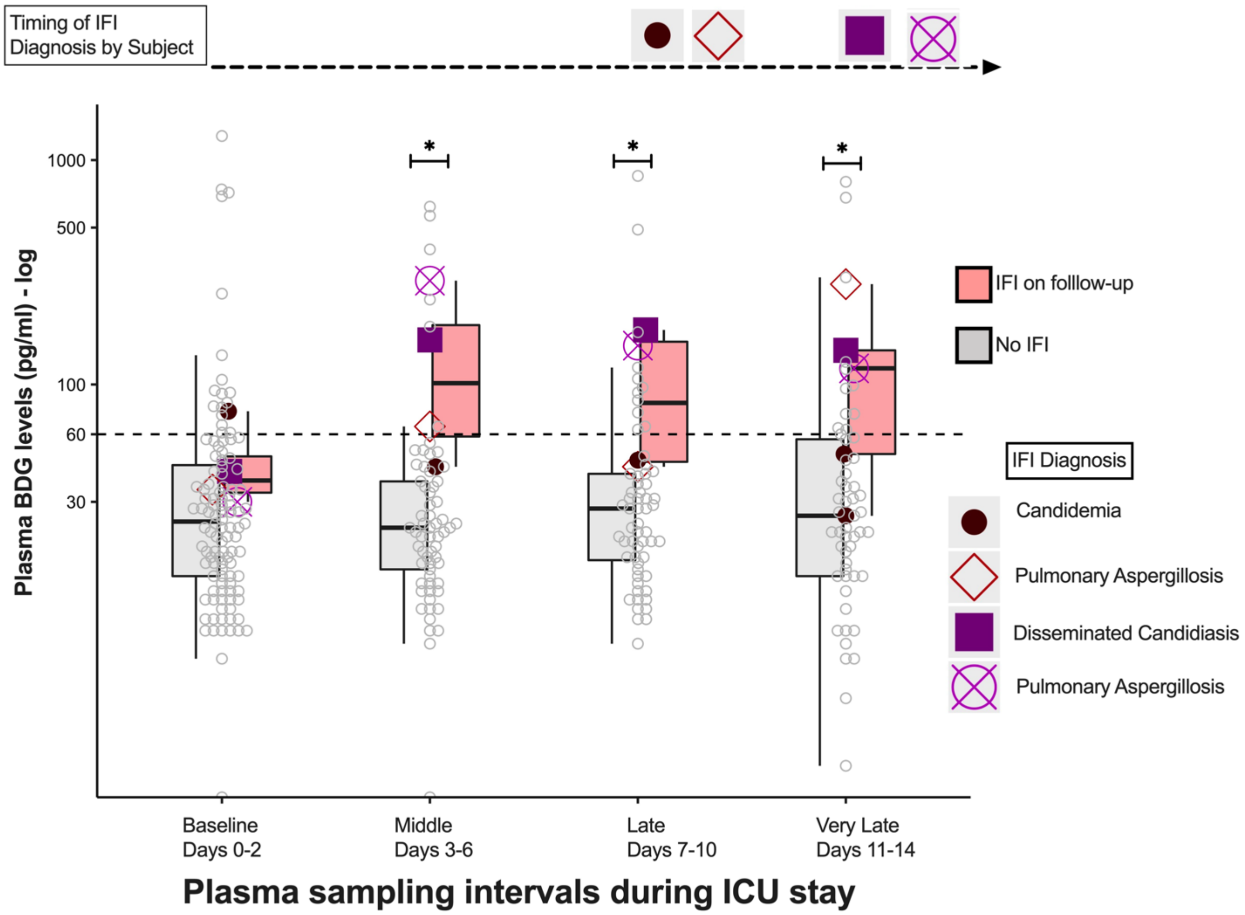

Figure S11: Plasma BDG levels are associated with host inflammation in hospitalized patients with COVID-19. A. Correlogram depicting direction and statistical significance of correlations between BDG and plasma biomarkers of inflammation, following adjustment for multiple comparisons with the Benjamini-Hochberg method. Positive correlations are shown in red and negative correlations in blue. Nonsignificant results are marked with an X. B. Hyperinflammatory patients had higher BDG levels (median [Interquartile range] = 29.5 [24.8-38.0]) than hypoinflammatory patients (20.0 [13.0-25.0], p=0.004). Probabilistic Graphical Model analysis demonstrating first and second neighbors of the BDG variable, when considered in conjunction with 28 clinical and biomarker variables. Four first neighbors were identified: two clinical variables (Diabetes and Platelets) and the same two biomarkers (TNFR1 and ST-2) as in the discovery cohort of Acute Respiratory Failure. This finding offered further validation for the involvement of BDG in host inflammation in the pathways involving TNFR1 and ST-2. Abbreviations: IL-6: Interleukin-6; IL-10: Interleukin-10; TNFR-1: Tumor necrosis factor receptor-1; Ang-2: Angiopoietin-2; ST-2: Suppression of tumorigenicity-2; RAGE: Receptor for advanced glycation end products.

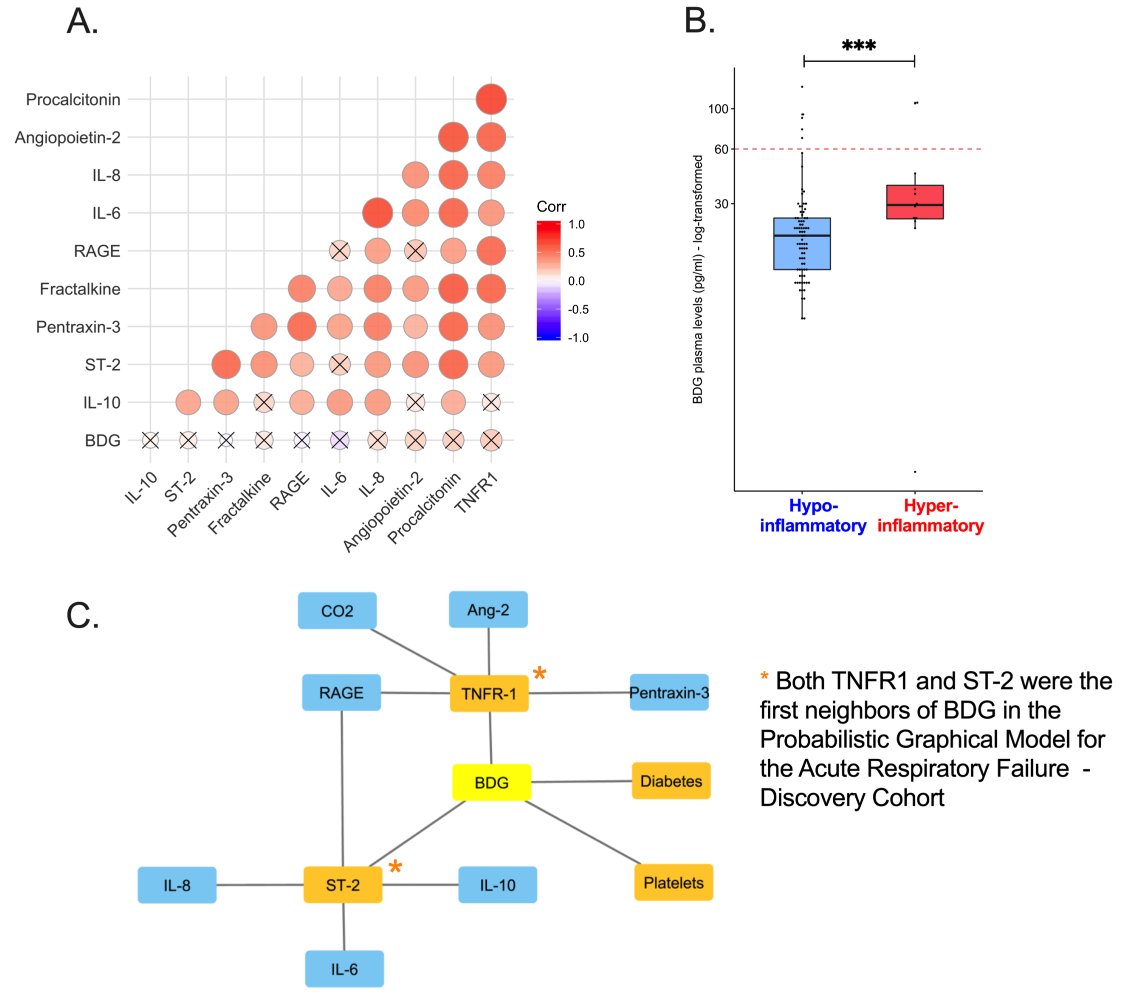

Figure 12. Correlations of BDG with host inflammation in patients with Traumatic Brain Injury (TBI) and integrative analysis for BDG associations with Probabilistic Graphical Models (PGM). A. Correlogram depicting direction and statistical significance of correlations between BDG and serum biomarkers of inflammation, following adjustment for multiple comparisons with the Benjamini-Hochberg method. Positive correlations are shown in red and negative correlations in blue. Nonsignificant results are marked with an X. BDG had weak statistically significant correlations with IL-8 and Neutrophil/Leukocyte Ratio (NLR). Probabilistic Graphical Model analysis demonstrating first and second neighbors of the BDG variable, when considered in conjunction with 21 clinical and biomarker variables. BDG was first neighbor with a single clinical variable, Injury Severity Score (ISS), a validated clinical predictor of outcome in TBI.

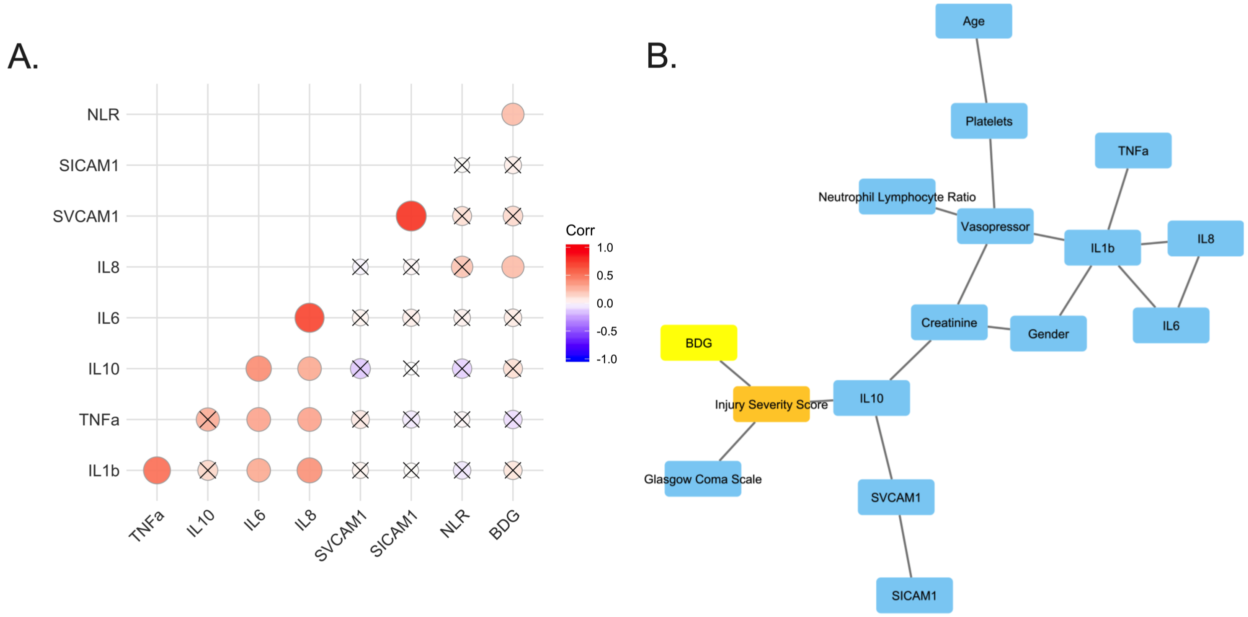

Abbreviations: IL: interleukin, SICAM1: Soluble intercellular adhesion molecule 1, SVCAM1 - soluble vascular cell adhesion molecule 1, TNFa: tumor necrosis factor alpha

Figure S13: BDG levels and correlations with inflammatory biomarkers in the TBI cohort, with patient stratification into those with TBI only (n=19, 18%) vs. TBI with polytrauma (n=88, 82%). A. Patients with TBI with polytrauma (non- head and neck Injury Severity Score of >3) had a trend towards higher levels of BDG compared to patients with TBI only. B. Serum IL-8 had non-significant correlations with BDG in opposite directions in patients with TBI only vs. patients with TBI with polytrauma. C. B. Neutrophil/Lymphocyte ratio was significantly correlated with BDG in patients with TBI with polytrauma.

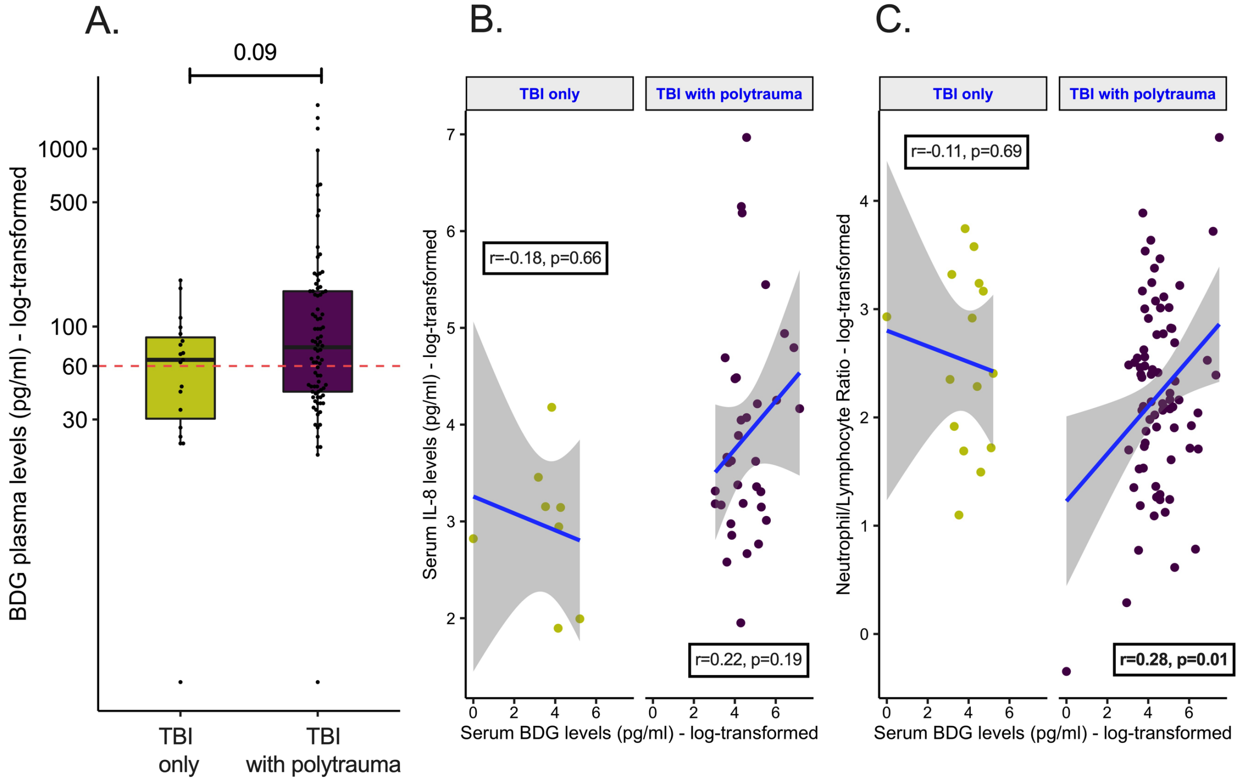

Table S1. Baseline characteristics of enrolled subjects in the Acute Respiratory Failure cohort, stratified in two different ways: i) by applying standard cut-offs of BDG test positivity for the diagnosis of invasive fungal infection (i.e. <60 pg/ml negative, 61-79 pg/ml indeterminate, ≥80 pg/ml positive), and ii) by the newly derived BDG cut-off of 40pg/ml for the prediction of the hyperinflammatory subphenotype. We performed non-parametric tests (Kruskal-Wallis and Wilcoxon tests for continuous variables between the three and two groups, respectively; Fisher's exact tests for categorical variables) and highlight statistically significant results (p<0.05) in bold.

	Negative (<60)	Indeterminate (61-80)	Positive (≥80)	p-value	Low (< 40)	High (≥ 40)	p-value
N	368	26	59	-	313	140	-
Age (median [IQR])	57.4 [46.5, 67.5]	59.5 [37.8, 66.2]	60.3 [46.3, 65.9]	0.8	57.0 [46.3, 66.9]	60.2 [45.0, 67.4]	0.46
Male (%)	196 (53.3)	13 (50.0)	35 (59.3)	0.63	168 (53.7)	76 (54.3)	0.99
BMI (median [IQR])	29.1 [24.8, 36.5]	28.1 [25.8, 32.4]	27.2 [24.7, 31.4]	0.11	29.1 [25.2, 36.4]	27.8 [24.7, 32.7]	**0.04**
History of COPD (%)	90 (24.5)	8 (30.8)	10 (16.9)	0.32	75 (24.0)	33 (23.6)	1
History of diabetes mellitus (%)	121 (32.9)	10 (38.5)	19 (32.2)	0.83	99 (31.6)	51 (36.4)	0.37
History of immunosuppression (%)	66 (17.9)	6 (23.1)	16 (27.1)	0.23	50 (16.0)	38 (27.1)	**0.01**
Clinical Group classification (%)				0.99			0.86
Acute on chronic respiratory failure	19 (5.2)	2 (7.7)	3 (5.1)		18 (5.8)	6 (4.3)	
ARDS	95 (25.8)	7 (26.9)	18 (30.5)		79 (25.2)	41 (29.3)	
At Risk for ARDS	142 (38.6)	10 (38.5)	20 (33.9)		117 (37.4)	55 (39.3)	
Control - Airway Protection	54 (14.7)	4 (15.4)	9 (15.3)		49 (15.7)	18 (12.9)	
Control - CHF	29 (7.9)	2 (7.7)	3 (5.1)		25 (8.0)	9 (6.4)	
Other	29 (7.9)	1 (3.8)	6 (10.2)		25 (8.0)	11 (7.9)	
SOFA Score (median [IQR])	6.0 [4.0, 9.0]	9.0 [5.2, 11.0]	7.0 [5.0, 10.0]	**0.01**	6.0 [4.0, 9.0]	7.0 [5.0, 10.0]	**<0.01**
P:F Ratio (median [IQR])	165 [116, 206]	175 [106, 204]	163 [121, 205]	0.97	164 [113, 205]	164.0 [120, 222]	0.58
Beta-lactam on study day 1 (%)	270 (74.2)	21 (80.8)	49 (83.1)	0.28	232 (75.1)	108 (77.1)	0.72
Topical antifungal on study day 1 (%)	9 (2.5)	2 (7.7)	2 (3.4)	0.3	8 (2.6)	5 (3.6)	0.79
Systemic antifungal on study day 1 (%)	2 (0.5)	0 (0.0)	2 (3.4)	0.09	2 (0.6)	2 (1.4)	0.78
Positive respiratory culture for bacterial pathogens (%)	84 (22.8)	7 (26.9)	14 (23.7)	0.89	73 (23.3)	32 (22.9)	1
Incident shock (%)	178 (48.4)	17 (65.4)	31 (52.5)	0.22	151 (48.2)	75 (53.6)	0.34
Bacteremia (%)	37 (10.1)	3 (11.5)	4 (6.8)	0.69	27 (8.6)	17 (12.1)	0.32
Hyperinflammatory subphenotype (%)	89 (24.2)	14 (53.8)	24 (40.7)	**<0.001**	66 (21.1)	61 (43.6)	**<0.001**

BMI: Body mass index, SOFA Score: Sequential organ failure assessment score, P:F Ratio: Ratio of partial pressure of arterial oxygen and fraction of inspired oxygen, COPD: Chronic obstructive pulmonary, ARDS: Acute Respiratory Distress Syndrome, CHF: Congestive heart failure

Table S2: Baseline characteristics for patients with COVID-19.

n	97
Age (median [IQR])	63.0 [56.0, 73.0]
Male (%)	47 (48.5)
Whites (%)	23 (23.7)
Blacks (%)	73 (73.2)
Unknown race (%)	3 (3.1)
BMI (median [IQR])	31.2 [26.8, 37.9]
SOFA Score (median [IQR])	6.0 [4.0, 8.0]
Lowest PaO2 (median [IQR])	70.5 [58.2, 86.5]
History of COPD (%)	16 (16.5)
History of diabetes mellitus (%)	37 (38.1)
History of immunosuppression (%)	12 (12.4)
Mechanically-ventilated (%)	41 (42.2)
WBC (median [IQR])	6.4 [4.8, 9.7]
WHO ordinal scale of severity on admission (median [IQR])	5.0 [5.0, 7.0]
Hyperinflammatory subphenotype (%)	12 (13.2)

BMI: Body mass index, SOFA Score: Sequential organ failure assessment score; PaO2: partial pressure of arterial oxygen, COPD: Chronic obstructive pulmonary disease; NLR: Neutrophil to lymphocyte ratio; WBC: White cell count; WHO: World Health Organization

Table S3: Baseline characteristics for patients with traumatic brain injury (TBI)

n	137
Age (median [IQR])	38.0 [24.0, 52.0]
Whites (%)	124 (90.5)
Blacks (%)	10 (7.3)
Unknown race (%)	1 (0.7)
Male (%)	114 (83.2)
BMI (median [IQR])	25.9 [22.9, 29.5]
P:F Ratio (median [IQR]) (median [IQR])	240.0 [156.7, 372.0]
Best GCS in first 24 hours (median [IQR])	7.0 [5.0, 8.0]
ISS (median [IQR])	33.0 [26.0, 38.0]
ISS, non head-trauma (median [IQR])	10.0 [4.0, 19.5]
Platelets (median [IQR])	204.0 [143.0, 247.0]
Absolute neutrophil count (median [IQR])	12.7 [9.5, 16.2]
Absolute lymphocyte count (median [IQR])	1.2 [0.8, 2.1]
NLR (median [IQR])	9.7 [5.5, 18.0]
WBC (median [IQR])	16.5 [12.6, 20.1]
Creatinine (median [IQR])	1.0 [0.8, 1.2]
Incident shock (%)	54 (39.4)

BMI: Body mass index, SOFA Score: Sequential organ failure assessment score, P:F Ratio: Ratio of partial pressure of arterial oxygen and fraction of inspired oxygen, GCS: Glasgow coma scale; ISS: injury severity score, NLR: Neutrophil to lymphocyte ratio, WBC: White cell count.

Bibliography

1. Kitsios GD et al. Respiratory Tract Dysbiosis Is Associated with Worse Outcomes in Mechanically Ventilated Patients.. *Am. J. Respir. Crit. Care Med.* 2020;202(12):1666–1677.

2. Fair K et al. Rectal Swabs from Critically Ill Patients Provide Discordant Representations of the Gut Microbiome Compared to Stool Samples.. *mSphere* 2019;4(4). doi:10.1128/mSphere.00358-19

3. Kitsios GD et al. Respiratory microbiome profiling for etiologic diagnosis of pneumonia in mechanically ventilated patients.. *Front. Microbiol.* 2018;9:1413.

4. Schoch CL et al. Nuclear ribosomal internal transcribed spacer (ITS) region as a universal DNA barcode marker for Fungi.. *Proc Natl Acad Sci USA* 2012;109(16):6241–6246.

5. Caporaso JG et al. Ultra-high-throughput microbial community analysis on the Illumina HiSeq and MiSeq platforms.. *ISME J.* 2012;6(8):1621–1624.

6. Callahan BJ et al. DADA2: High-resolution sample inference from Illumina amplicon data.. *Nat. Methods* 2016;13(7):581–583.

7. Wang Q, Garrity GM, Tiedje JM, Cole JR. Naive Bayesian classifier for rapid assignment of rRNA sequences into the new bacterial taxonomy.. *Appl. Environ. Microbiol.* 2007;73(16):5261–5267.

8. Finkelman MA. Specificity Influences in (1→3)-β-d-Glucan-Supported Diagnosis of Invasive Fungal Disease.. *J. Fungi (Basel)* 2020;7(1). doi:10.3390/jof7010014
