# Supplementary material for: Plasma 1,3-β-d-glucan levels predict adverse clinical outcomes in critical illness
Source: JCI Insight. 2021 Jul 22;6(14):e141277. doi: 10.1172/jci.insight.141277 (PMC8410081; doi:10.1172/jci.insight.141277)
Supplement: Trial reporting checklists [file jciinsight-6-141277-s083.pdf]

STROBE Statement—checklist of items that should be included in reports of observational studies

	Item No.	Recommendation	Page No.	Relevant text from manuscript
Title and abstract	1	(*a*) Indicate the study's design with a commonly used term in the title or the abstract	1	"associated", "predict"
		(*b*) Provide in the abstract an informative and balanced summary of what was done and what was found	2	
Introduction				
Background/rationale	2	Explain the scientific background and rationale for the investigation being reported	4, 5	
Objectives	3	State specific objectives, including any prespecified hypotheses	5	"whether BDG could be used as a clinical biomarker of fungal PAMP translocation and its contribution to the development and perpetuation of hyperinflammatory host-responses in critical illness is unknown. "
Methods				
Study design	4	Present key elements of study design early in the paper	5	We analyzed data from 453 mechanically-ventilated patients with acute respiratory failure (ARF, *discovery cohort*), who were consecutively enrolled from ICUs at the University of Pittsburgh Medical Center (UPMC)
Setting	5	Describe the setting, locations, and relevant dates, including periods of recruitment, exposure, follow-up, and data collection	15	From October 2011 – June 2019, we prospectively enrolled a convenience sample of consecutive, adult patients with ARF, who were intubated and mechanically-ventilated in the Medical or Cardiac Intensive Care Units (ICU) at the University of Pittsburgh Medical Center (UPMC). Exclusion criteria included inability to obtain informed consent,

				presence of tracheostomy, or mechanical ventilation for more than 72 hours prior to enrollment. From 453 enrolled subjects, we collected blood samples for centrifugation and separation of plasma and WBC pellets. We also collected noninvasive biospecimens for study of lower respiratory tract microbiota with ETA and we analyzed stool samples for study of the intestinal microbiota, as previously described (10, 55, 56). From all subjects we collected biospecimens at a baseline interval (0-2 days post-intubation), and when possible, we collected follow-up blood samples at a middle interval (3-6 days post-intubation, n=156). For the subset of patients with prolonged ICU stay (>14 days, n=113), we also analyzed available blood biospecimens at four consecutive time intervals post-intubation: baseline (0-2 days, n=113), middle (3-6 days, n=68), late (7-10 days, n=63), and very late (11-14 days, n=58).
Participants	6	(*a*) *Cohort study*—Give the eligibility criteria, and the sources and methods of selection of participants. Describe methods of follow-up	15, 17	we prospectively enrolled a convenience sample of consecutive, adult patients with ARF, who were intubated and mechanically-ventilated in the Medical or Cardiac Intensive Care Units (ICU) at the University of Pittsburgh Medical Center (UPMC). Exclusion criteria included

				inability to obtain informed consent, presence of tracheostomy, or mechanical ventilation for more than 72 hours prior to enrollment.<br><br>We followed patients prospectively for incidence of IFI, AKI, VFD at 30 days, and 30-day mortality.
		(*b*) *Cohort study*—For matched studies, give matching criteria and number of exposed and unexposed	NA	
Variables	7	Clearly define all outcomes, exposures, predictors, potential confounders, and effect modifiers. Give diagnostic criteria, if applicable	17	A consensus committee reviewed clinical and radiographic data and performed retrospective classifications of the etiology and severity of ARF. We performed classifications without knowledge of biomarker data. We retrospectively classified ARF as *ARDS* per established criteria (58), *at risk for ARDS* due to the presence of direct (pneumonia or aspiration) or indirect (e.g. extra-pulmonary sepsis or acute pancreatitis) lung injury risk factors (59) but lacking ARDS diagnostic criteria, *ARF without risk factors for ARDS*, or *acute on chronic respiratory failure*. We recorded clinical microbiologic results of respiratory and blood culture specimens obtained within 48hrs of research sample acquisition. We excluded patients with diagnosis of IFI based on microbiologic

				evidence and consistent clinical picture (23). We recorded antibiotic prescriptions, including beta-lactams associated with false-positive BDG tests, topical antifungals for skin candidiasis or oral thrush, and systemic antifungals as part of empiric antimicrobial regimens. We followed patients prospectively for incidence of IFI, AKI, VFD at 30 days, and 30-day mortality
Data sources/ measurement	8*	For each variable of interest, give sources of data and details of methods of assessment (measurement). Describe comparability of assessment methods if there is more than one group	*16*	Detailed laboratory methods for the molecular assays used are provided in the Supplement. For all available biospecimens, we measured BDG levels using the commercially available Fungitell® Limulus Amebocyte Lysate (LAL) assay (Associates of Cape Cod, Inc, East Falmouth, MA, USA) at the manufacturer's facility. As a biomarker of intestinal permeability, we measured levels FABP2 with the Quantikine® Human FABP2/I-FABP Immunoassay (R&D Systems, Minneapolis, USA). For profiling host-responses in the ARF and COVID-19 cohorts, we constructed a custom Luminex multi-analyte panel (R&D Systems, Minneapolis) targeting biomarkers associated with ARDS outcomes (RAGE, IL-6, IL-8, IL-10, TNFR1, ST-2, fractalkine, angiopoietin-2,

				procalcitonin and pentraxin-3), as previously described
Bias	9	Describe any efforts to address potential sources of bias	17	. We used linear regression models to examine for associations between BDG levels and biomarkers of host responses. Multiple testing adjustments were implemented with the Benjamini-Hochberg method. We examined for differences in BDG levels between hyper-inflammatory and hypo-inflammatory subphenotypes using Wilcoxon tests. We examined for associations between BDG levels and clinical outcomes with logistic regression models for AKI and 30-day mortality, zero-inflated negative binomial models for VFD, and Cox-proportional hazard models for 30-day survival, adjusted for clinical predictors (e.g. age, sex and SOFA score or subphenotype classification) as well as potential confounders of BDG measurement (beta-lactam antibiotics and batch of BDG measurement). We examined for association between BDG levels and RAGE (marker of alveolar epithelial damage) using a linear regression adjusted for yeast growth in respiratory cultures and alpha diversity by sequencing. For the association between BDG and FABP2 (marker of intestinal permeability), we built a linear regression model adjusted for

				variables that may impact the integrity of the intestinal epithelium (receipt of vasopressors) and fungal overgrowth in the gut (systemic antibiotics).
Study size	10	Explain how the study size was arrived at	15	Discovery cohort: From October 2011 – June 2019, we prospectively enrolled a convenience sample of consecutive, adult patients with ARF, who were intubated and mechanically-ventilated in the Medical or Cardiac Intensive Care Units (ICU) at the University of Pittsburgh Medical Center (UPMC). Exclusion criteria included inability to obtain informed consent, presence of tracheostomy, or mechanical ventilation for more than 72 hours prior to enrollment. From 453 enrolled subjects, we collected blood samples for centrifugation and separation of plasma and WBC pellets

Continued on next page

Quantitative variables	11	Explain how quantitative variables were handled in the analyses. If applicable, describe which groupings were chosen and why	17	We classified baseline plasma samples based on the standard cut-offs of BDG test positivity for the diagnosis of IFI (i.e. <60 pg/ml negative, 60-79 pg/ml indeterminate, ≥80 pg/ml positive). We log-transformed BDG and biomarker values for use in regression models. From available biomarker and clinical variables, we classified patients into hyper-inflammatory vs. hypo-inflammatory subphenotype based on an internally derived and validated parsimonious logistic regression model, following application of latent class analyses
Statistical methods	12	(*a*) Describe all statistical methods, including those used to control for confounding	17	We examined for associations between BDG levels and clinical outcomes with logistic regression models for AKI and 30-day mortality, zero-inflated negative binomial models for VFD, and Cox-proportional hazard models for 30-day survival, adjusted for clinical predictors (e.g. age, sex and SOFA score or subphenotype classification) as well as potential confounders of BDG measurement (beta-lactam antibiotics and batch of BDG measurement). We examined for association between BDG levels and RAGE (marker of alveolar epithelial damage) using a linear regression adjusted for yeast growth in respiratory cultures and alpha diversity by sequencing. For the association between BDG and FABP2 (marker of intestinal permeability), we built a linear regression model adjusted for

				variables that may impact the integrity of the intestinal epithelium (receipt of vasopressors) and fungal overgrowth in the gut (systemic antibiotics).
		(*b*) Describe any methods used to examine subgroups and interactions	NA	
		(*c*) Explain how missing data were addressed	NA	
		(*d*) *Cohort study*—If applicable, explain how loss to follow-up was addressed strategy	15	From all subjects we collected biospecimens at a baseline interval (0-2 days post-intubation), and when possible, we collected follow-up blood samples at a middle interval (3-6 days post-intubation, n=156). For the subset of patients with prolonged ICU stay (>14 days, n=113), we also analyzed available blood biospecimens at four consecutive time intervals post-intubation: baseline (0-2 days, n=113), middle (3-6 days, n=68), late (7-10 days, n=63), and very late (11-14 days, n=58).
		(*e*) Describe any sensitivity analyses	9	When patients with BDG≥40pg/ml were further stratified by standard cut-off points for clinical diagnosis of IFI, we found that BDG≥80pg/ml (positive test) had significantly worse survival than patients with BDG<40pg/ml (adjusted HR 2.43 [1.54-3.85, p<0.0001, Figure 5B), whereas patients with intermediate range BDG levels (40-59 or 60-79 pg/ml) had similar survival between the other two groups.
Results				
Participants	13*	(a) Report numbers of individuals at each stage of study—eg numbers potentially eligible, examined for eligibility, confirmed eligible, included in the study, completing follow-up, and analysed	5	*Study population*: We analyzed data from 453 mechanically-ventilated patients with acute respiratory failure (ARF, *discovery cohort*),

				who were consecutively enrolled from ICUs at the University of Pittsburgh Medical Center (UPMC) (3, 10, 24). We excluded 11 patients with a clinical diagnosis of IFI *a priori* because we wanted to examine BDG as a fungal PAMP in a broad ICU population and not in the context of IFI as a diagnostic test. Characteristics of ARF patients in the discovery cohort are shown in Table 1. We also included data from two independent validation cohorts: i) 97 patients with acute respiratory failure from COVID-19 (25), and ii) 137 mechanically ventilated patients following severe traumatic brain injury (TBI) (26). Characteristics of patients in the validation cohorts are shown in Tables S2 and S3
		(b) Give reasons for non-participation at each stage	NA	
		(c) Consider use of a flow diagram	NA	
Descriptive data	14*	(a) Give characteristics of study participants (eg demographic, clinical, social) and information on exposures and potential confounders	5	Characteristics of ARF patients in the discovery cohort are shown in Table 1.
		(b) Indicate number of participants with missing data for each variable of interest	NA	
		(c) *Cohort study*—Summarise follow-up time (eg, average and total amount)	9	For a smaller subset of ARF patients who had prolonged ICU stay (>14 days), we measured BDG levels in research samples obtained from four time intervals post-intubation: baseline (0-2 days, n=113), middle (3-6 days, n=68), late (7-10 days, n=63), very late (11-14 days, n=58).
Outcome data	15*	*Cohort study*—Report numbers of outcome events or summary measures over time	*23*	Figure 5: Patients with a high plasma BDG levels (≥40 pg/ml) at baseline had worse 30-day survival post-intubation compared to

				patients with low (<40 pg/ml) BDG levels.
Main results	16	(*a*) Give unadjusted estimates and, if applicable, confounder-adjusted estimates and their precision (eg, 95% confidence interval). Make clear which confounders were adjusted for and why they were included	21	Table 2
		(*b*) Report category boundaries when continuous variables were categorized	5	By applying standard cut-offs of BDG test positivity for the diagnosis of IFI in the ARF cohort (i.e. <60 pg/ml negative, 61-79 pg/ml indeterminate, ≥80 pg/ml positive), 81.2% of baseline samples were negative, 5.7% indeterminate and 13.0% positive, with no significant differences in clinical characteristics between groups apart from higher sequential organ failure assessment (SOFA) scores in the indeterminate and positive group (p=0.02, Table S3).
		(*c*) If relevant, consider translating estimates of relative risk into absolute risk for a meaningful time period	NA	

Continued on next page

Other analyses	17	Report other analyses done—eg analyses of subgroups and interactions, and sensitivity analyses	6	Although beta-lactam antibiotics have been previously implicated in false-positive BDG detection (32), we did not find any differences in BDG levels between patients receiving beta-lactam antibiotics, or not, at the time of sampling. A small proportion of patients (2.9%) receiving topical antifungals for either oral thrush or skin candidiasis (clotrimazole troche or nystatin) had significantly higher BDG levels compared to those not receiving these medications (p=0.03). Only four patients were on systemic antifungals at the time of sampling (Table 1). We did not identify any significant effect of year of sample acquisition, experimental batch or time of hospital or ICU admission to sample acquisition on measured BDG levels (Figures S2-3).
Discussion				
Key results	18	Summarise key results with reference to study objectives	10	We demonstrated that mechanically ventilated, medical ICU patients with ARF have higher circulating BDG compared to healthy controls, in the absence of IFI at the time of sampling. BDG levels were significantly associated with the adverse hyperinflammatory subphenotype, and this novel association was validated in an independent cohort of patients with COVID-19. By integrating analyses of culture-dependent and independent methods of fungal colonization as well as biomarkers of epithelial permeability, we did not identify a single primary putative

				source of translocating BDG but detected significant associations for both the respiratory and intestinal compartments. Baseline BDG levels independently predicted adverse clinical outcomes, whereas serial BDG levels revealed that patients with incident BDG elevation had worse survival than patients with persistently negative BDG testing.
Limitations	19	Discuss limitations of the study, taking into account sources of potential bias or imprecision. Discuss both direction and magnitude of any potential bias	14	Our study has several limitations. As a single-center study, generalizability of our findings in critically ill populations beyond our tertiary care institution requires external validation in other centers. Our study is also limited by the available sample size. Despite being the largest study of BDG measurement in ARF, results from analyses for subgroups (e.g. longitudinal sample analyses) and specific biomarkers (e.g. FABP-2) require cautious interpretation, as the effective sample size for certain analyses was smaller. Although we aimed to control for potential confounders of BDG measurement from available variables (batch of measurement, beta-lactam antibiotics or bacteremia) (32), there may be other sources of BDG false positivity, such as hemodialysis or blood product preparation filter processing that were not possible to account for in our analyses (53, 54). Finally, the extent of microbiologic workup for diagnosis of bacterial infection or IFI was directed by the treating physicians and, as such,

				microbiologic testing was not uniform. Thus, it is possible that some of the cases with high BDG levels could represent clinically undiagnosed or preclinical IFI, as revealed in a small number of subjects with incident IFI diagnosis in our longitudinal analyses.
Interpretation	20	Give a cautious overall interpretation of results considering objectives, limitations, multiplicity of analyses, results from similar studies, and other relevant evidence	14	In summary, we demonstrate that circulating BDG is an independent predictor of a hyperinflammatory host-response profile and adverse clinical outcomes in mechanically ventilated medical patients with acute respiratory failure. We validated that BDG is associated with host inflammation in patients with COVID-19. These findings highlight a potentially underappreciated mechanism of biological heterogeneity in critical illness that involves interactions between translocating fungal PAMPs and innate immunity, and point towards complex interactions between the host, fungi and other microbes at different mucosal interfaces. Our findings call for further mechanistic interrogation in animal studies and independent prospective clinical study to validate the biologic relevance and prognostic information of circulating BDG.
Generalisability	21	Discuss the generalisability (external validity) of the study results	14	We validated that BDG is associated with host inflammation in patients with COVID-19.
Other information				

Funding	22	Give the source of funding and the role of the funders for the present study and, if applicable, for the original study on which the present article is based	2	Funding support: University of Pittsburgh Clinical and Translational Science Institute, COVID-19 Pilot Award (GDK); National Institutes of Health [K23 HL139987 (GDK); U01 HL098962 (AM); P01 HL114453 (BJM); R01 HL097376 (BJM); K24 HL123342 (AM); U01 HL137159 (PVB); R01 LM012087 (PVB); K08HK144820 (JWE); F32 HL142172 (WB); K23 GM122069 (FS)].

*Give information separately for cases and controls in case-control studies and, if applicable, for exposed and unexposed groups in cohort and cross-sectional studies.

Note: An Explanation and Elaboration article discusses each checklist item and gives methodological background and published examples of transparent reporting. The STROBE checklist is best used in conjunction with this article (freely available on the Web sites of PLoS Medicine at http://www.plosmedicine.org/, Annals of Internal Medicine at http://www.annals.org/, and Epidemiology at http://www.epidem.com/). Information on the STROBE Initiative is available at www.strobe-statement.org.
