# Supplementary material for: Plasma 1,3-β-d-glucan levels predict adverse clinical outcomes in critical illness
Source: JCI Insight. 2021 Jul 22;6(14):e141277. doi: 10.1172/jci.insight.141277 (PMC8410081; doi:10.1172/jci.insight.141277)
Supplement: ICMJE disclosure forms [file jciinsight-6-141277-s084.pdf]

ICMJE DISCLOSURE FORM

Date:_______________4/29/2021____
Your Name:____________GEORGIOS KITSIOS ____
Manuscript Title:_______ Plasma 1,3-beta-D-glucan levels are associated with host inflammatory responses and predict adverse clinical outcomes in critical illness _______________________________________
Manuscript number (if known):_______ 141277-INS-RG-1____

In the interest of transparency, we ask you to disclose all relationships/activities/interests listed below that are related to the content of your manuscript. "Related" means any relation with for-profit or not-for-profit third parties whose interests may be affected by the content of the manuscript. Disclosure represents a commitment to transparency and does not necessarily indicate a bias. If you are in doubt about whether to list a relationship/activity/interest, it is preferable that you do so.

The following questions apply to the author's relationships/activities/interests as they relate to the current manuscript only.

The author's relationships/activities/interests should be defined broadly. For example, if your manuscript pertains to the epidemiology of hypertension, you should declare all relationships with manufacturers of antihypertensive medication, even if that medication is not mentioned in the manuscript.

In item #1 below, report all support for the work reported in this manuscript without time limit. For all other items, the time frame for disclosure is the past 36 months.

		**Name all entities with whom you have this relationship or indicate none (add rows as needed)**	**Specifications/Comments (e.g., if payments were made to you or to your institution)**
		**Time frame: Since the initial planning of the work**	
1	All support for the present manuscript (e.g., funding, provision of study materials, medical writing, article processing charges, etc.) **No time limit for this item.**	__X_None	
		**Time frame: past 36 months**	
2	Grants or contracts from any entity (if not indicated in item #1 above).	NIH	
		Karius, Inc	
3	Royalties or licenses	__X__None	

4	Consulting fees	__X__None	
5	Payment or honoraria for lectures, presentations, speakers bureaus, manuscript writing or educational events	_X___None	
6	Payment for expert testimony	_X___None	
7	Support for attending meetings and/or travel	__X__None	
8	Patents planned, issued or pending	_X___None	
9	Participation on a Data Safety Monitoring Board or Advisory Board	_X___None	
10	Leadership or fiduciary role in other board, society, committee or advocacy group, paid or unpaid	_X___None	
11	Stock or stock options	X____None	
12	Receipt of equipment, materials, drugs, medical writing, gifts or other services	_X___None	
13	Other financial or non-financial interests	__X__None	

Please place an "X" next to the following statement to indicate your agreement:

X___ I certify that I have answered every question and have not altered the wording of any of the questions on this form.

ICMJE DISCLOSURE FORM

Date:_______________4/29/2021____
Your Name:____________DANIEL KOTOK____
Manuscript Title:_______ Plasma 1,3-beta-D-glucan levels are associated with host inflammatory responses and predict adverse clinical outcomes in critical illness _______________________________________
Manuscript number (if known):_______ 141277-INS-RG-1____

In the interest of transparency, we ask you to disclose all relationships/activities/interests listed below that are related to the content of your manuscript. "Related" means any relation with for-profit or not-for-profit third parties whose interests may be affected by the content of the manuscript. Disclosure represents a commitment to transparency and does not necessarily indicate a bias. If you are in doubt about whether to list a relationship/activity/interest, it is preferable that you do so.

The following questions apply to the author's relationships/activities/interests as they relate to the current manuscript only.

The author's relationships/activities/interests should be defined broadly. For example, if your manuscript pertains to the epidemiology of hypertension, you should declare all relationships with manufacturers of antihypertensive medication, even if that medication is not mentioned in the manuscript.

In item #1 below, report all support for the work reported in this manuscript without time limit. For all other items, the time frame for disclosure is the past 36 months.

		Name all entities with whom you have this relationship or indicate none (add rows as needed)	Specifications/Comments (e.g., if payments were made to you or to your institution)
**Time frame: Since the initial planning of the work**			
1	All support for the present manuscript (e.g., funding, provision of study materials, medical writing, article processing charges, etc.) **No time limit for this item.**	__X_None	
**Time frame: past 36 months**			
2	Grants or contracts from any entity (if not indicated in item #1 above).	__X__None	
3	Royalties or licenses	__X__None	
4	Consulting fees	__X__None	

5	Payment or honoraria for lectures, presentations, speakers bureaus, manuscript writing or educational events	_X___None	
6	Payment for expert testimony	_X___None	
7	Support for attending meetings and/or travel	__X__None	
8	Patents planned, issued or pending	_X___None	
9	Participation on a Data Safety Monitoring Board or Advisory Board	_X___None	
10	Leadership or fiduciary role in other board, society, committee or advocacy group, paid or unpaid	_X___None	
11	Stock or stock options	X____None	
12	Receipt of equipment, materials, drugs, medical writing, gifts or other services	_X___None	
13	Other financial or non-financial interests	__X__None	

Please place an "X" next to the following statement to indicate your agreement:

X___ I certify that I have answered every question and have not altered the wording of any of the questions on this form.

ICMJE DISCLOSURE FORM

Date:_______________4/29/2021____
Your Name:____________DANIEL KOTOK____
Manuscript Title:_______ Plasma 1,3-beta-D-glucan levels are associated with host inflammatory responses and predict adverse clinical outcomes in critical illness _______________________________________
Manuscript number (if known):_______ 141277-INS-RG-1____

In the interest of transparency, we ask you to disclose all relationships/activities/interests listed below that are related to the content of your manuscript. "Related" means any relation with for-profit or not-for-profit third parties whose interests may be affected by the content of the manuscript. Disclosure represents a commitment to transparency and does not necessarily indicate a bias. If you are in doubt about whether to list a relationship/activity/interest, it is preferable that you do so.

The following questions apply to the author's relationships/activities/interests as they relate to the <u>current manuscript only</u>.

The author's relationships/activities/interests should be <u>defined broadly</u>. For example, if your manuscript pertains to the epidemiology of hypertension, you should declare all relationships with manufacturers of antihypertensive medication, even if that medication is not mentioned in the manuscript.

In item #1 below, report all support for the work reported in this manuscript without time limit. For all other items, the time frame for disclosure is the past 36 months.

		**Name all entities with whom you have this relationship or indicate none (add rows as needed)**	**Specifications/Comments (e.g., if payments were made to you or to your institution)**
**Time frame: Since the initial planning of the work**			
1	All support for the present manuscript (e.g., funding, provision of study materials, medical writing, article processing charges, etc.) **No time limit for this item.**	__X_None	
**Time frame: past 36 months**			
2	Grants or contracts from any entity (if not indicated in item #1 above).	__X__None	
3	Royalties or licenses	__X__None	
4	Consulting fees	__X__None	

5	Payment or honoraria for lectures, presentations, speakers bureaus, manuscript writing or educational events	_X___None	
6	Payment for expert testimony	_X___None	
7	Support for attending meetings and/or travel	__X__None	
8	Patents planned, issued or pending	_X___None	
9	Participation on a Data Safety Monitoring Board or Advisory Board	_X___None	
10	Leadership or fiduciary role in other board, society, committee or advocacy group, paid or unpaid	_X___None	
11	Stock or stock options	X____None	
12	Receipt of equipment, materials, drugs, medical writing, gifts or other services	_X___None	
13	Other financial or non-financial interests	__X__None	

Please place an "X" next to the following statement to indicate your agreement:

X___ I certify that I have answered every question and have not altered the wording of any of the questions on this form.

ICMJE DISCLOSURE FORM

Date:_______________4/29/2021____
Your Name:____________HAOPU YANG____
Manuscript Title:_______ Plasma 1,3-beta-D-glucan levels are associated with host inflammatory responses and predict adverse clinical outcomes in critical illness ____
Manuscript number (if known):_______ 141277-INS-RG-1____

In the interest of transparency, we ask you to disclose all relationships/activities/interests listed below that are related to the content of your manuscript. "Related" means any relation with for-profit or not-for-profit third parties whose interests may be affected by the content of the manuscript. Disclosure represents a commitment to transparency and does not necessarily indicate a bias. If you are in doubt about whether to list a relationship/activity/interest, it is preferable that you do so.

The following questions apply to the author's relationships/activities/interests as they relate to the current manuscript only.

The author's relationships/activities/interests should be defined broadly. For example, if your manuscript pertains to the epidemiology of hypertension, you should declare all relationships with manufacturers of antihypertensive medication, even if that medication is not mentioned in the manuscript.

In item #1 below, report all support for the work reported in this manuscript without time limit. For all other items, the time frame for disclosure is the past 36 months.

		**Name all entities with whom you have this relationship or indicate none (add rows as needed)**	**Specifications/Comments (e.g., if payments were made to you or to your institution)**
**Time frame: Since the initial planning of the work**			
1	All support for the present manuscript (e.g., funding, provision of study materials, medical writing, article processing charges, etc.) **No time limit for this item.**	__X_None	
**Time frame: past 36 months**			
2	Grants or contracts from any entity (if not indicated in item #1 above).	__X__None	
3	Royalties or licenses	__X__None	
4	Consulting fees	__X__None	

5	Payment or honoraria for lectures, presentations, speakers bureaus, manuscript writing or educational events	_X___None	
6	Payment for expert testimony	_X___None	
7	Support for attending meetings and/or travel	__X__None	
8	Patents planned, issued or pending	_X___None	
9	Participation on a Data Safety Monitoring Board or Advisory Board	_X___None	
10	Leadership or fiduciary role in other board, society, committee or advocacy group, paid or unpaid	_X___None	
11	Stock or stock options	X____None	
12	Receipt of equipment, materials, drugs, medical writing, gifts or other services	_X___None	
13	Other financial or non-financial interests	China Scholarship Council & Ministry of Education	Scholarship for study in the US

Please place an "X" next to the following statement to indicate your agreement:

X___ I certify that I have answered every question and have not altered the wording of any of the questions on this form.

ICMJE DISCLOSURE FORM

Date:_______________4/29/2021____
Your Name:____________MALCOLM FINKELMAN____
Manuscript Title:_______ Plasma 1,3-beta-D-glucan levels are associated with host inflammatory responses and predict adverse clinical outcomes in critical illness _______________________________________
Manuscript number (if known):_______ 141277-INS-RG-1____

In the interest of transparency, we ask you to disclose all relationships/activities/interests listed below that are related to the content of your manuscript. "Related" means any relation with for-profit or not-for-profit third parties whose interests may be affected by the content of the manuscript. Disclosure represents a commitment to transparency and does not necessarily indicate a bias. If you are in doubt about whether to list a relationship/activity/interest, it is preferable that you do so.

The following questions apply to the author's relationships/activities/interests as they relate to the current manuscript only.

The author's relationships/activities/interests should be defined broadly. For example, if your manuscript pertains to the epidemiology of hypertension, you should declare all relationships with manufacturers of antihypertensive medication, even if that medication is not mentioned in the manuscript.

In item #1 below, report all support for the work reported in this manuscript without time limit. For all other items, the time frame for disclosure is the past 36 months.

		Name all entities with whom you have this relationship or indicate none (add rows as needed)	Specifications/Comments (e.g., if payments were made to you or to your institution)
		**Time frame: Since the initial planning of the work**	
1	All support for the present manuscript (e.g., funding, provision of study materials, medical writing, article processing charges, etc.) **No time limit for this item.**	__X_None	
		**Time frame: past 36 months**	
2	Grants or contracts from any entity (if not indicated in item #1 above).	__X__None	
3	Royalties or licenses	__X__None	
4	Consulting fees	__X__None	

5	Payment or honoraria for lectures, presentations, speakers bureaus, manuscript writing or educational events	_X___None	
6	Payment for expert testimony	_X___None	
7	Support for attending meetings and/or travel	__X__None	
8	Patents planned, issued or pending	_X___None	
9	Participation on a Data Safety Monitoring Board or Advisory Board	_X___None	
10	Leadership or fiduciary role in other board, society, committee or advocacy group, paid or unpaid	_X___None	
11	Stock or stock options	X____None	
12	Receipt of equipment, materials, drugs, medical writing, gifts or other services	_X___None	
13	Other financial or non-financial interests	_Employment	Employement by the manufacturer of the BDG assay (Associates of Cape Cod, Inc, East Falmouth, MA, USA) used in this study ("Fungitell®")

Please place an "X" next to the following statement to indicate your agreement:

X___ I certify that I have answered every question and have not altered the wording of any of the questions on this form.

ICMJE DISCLOSURE FORM

Date:_______________4/29/2021____
Your Name:____________YONGLONG ZHANG____
Manuscript Title:_______ Plasma 1,3-beta-D-glucan levels are associated with host inflammatory responses and predict adverse clinical outcomes in critical illness ______________________________________
Manuscript number (if known):_______ 141277-INS-RG-1____

In the interest of transparency, we ask you to disclose all relationships/activities/interests listed below that are related to the content of your manuscript. "Related" means any relation with for-profit or not-for-profit third parties whose interests may be affected by the content of the manuscript. Disclosure represents a commitment to transparency and does not necessarily indicate a bias. If you are in doubt about whether to list a relationship/activity/interest, it is preferable that you do so.

The following questions apply to the author's relationships/activities/interests as they relate to the current manuscript only.

The author's relationships/activities/interests should be defined broadly. For example, if your manuscript pertains to the epidemiology of hypertension, you should declare all relationships with manufacturers of antihypertensive medication, even if that medication is not mentioned in the manuscript.

In item #1 below, report all support for the work reported in this manuscript without time limit. For all other items, the time frame for disclosure is the past 36 months.

		**Name all entities with whom you have this relationship or indicate none (add rows as needed)**	**Specifications/Comments (e.g., if payments were made to you or to your institution)**
		**Time frame: Since the initial planning of the work**	
1	All support for the present manuscript (e.g., funding, provision of study materials, medical writing, article processing charges, etc.) **No time limit for this item.**	__X_None	
		**Time frame: past 36 months**	
2	Grants or contracts from any entity (if not indicated in item #1 above).	__X__None	
3	Royalties or licenses	__X__None	
4	Consulting fees	__X__None	

5	Payment or honoraria for lectures, presentations, speakers bureaus, manuscript writing or educational events	_X___None	
6	Payment for expert testimony	_X___None	
7	Support for attending meetings and/or travel	__X__None	
8	Patents planned, issued or pending	_X___None	
9	Participation on a Data Safety Monitoring Board or Advisory Board	_X___None	
10	Leadership or fiduciary role in other board, society, committee or advocacy group, paid or unpaid	_X___None	
11	Stock or stock options	X____None	
12	Receipt of equipment, materials, drugs, medical writing, gifts or other services	_X___None	
13	Other financial or non-financial interests	_Employment	Employement by the manufacturer of the BDG assay (Associates of Cape Cod, Inc, East Falmouth, MA, USA) used in this study ("Fungitell®")

Please place an "X" next to the following statement to indicate your agreement:

X___ I certify that I have answered every question and have not altered the wording of any of the questions on this form.

ICMJE DISCLOSURE FORM

Date:_______________4/29/2021____
Your Name:____________Noel Britton____
Manuscript Title:_______ Plasma 1,3-beta-D-glucan levels are associated with host inflammatory responses and predict adverse clinical outcomes in critical illness ______________________________________
Manuscript number (if known):_______ 141277-INS-RG-1____

In the interest of transparency, we ask you to disclose all relationships/activities/interests listed below that are related to the content of your manuscript. "Related" means any relation with for-profit or not-for-profit third parties whose interests may be affected by the content of the manuscript. Disclosure represents a commitment to transparency and does not necessarily indicate a bias. If you are in doubt about whether to list a relationship/activity/interest, it is preferable that you do so.

The following questions apply to the author's relationships/activities/interests as they relate to the current manuscript only.

The author's relationships/activities/interests should be defined broadly. For example, if your manuscript pertains to the epidemiology of hypertension, you should declare all relationships with manufacturers of antihypertensive medication, even if that medication is not mentioned in the manuscript.

In item #1 below, report all support for the work reported in this manuscript without time limit. For all other items, the time frame for disclosure is the past 36 months.

		**Name all entities with whom you have this relationship or indicate none (add rows as needed)**	**Specifications/Comments (e.g., if payments were made to you or to your institution)**
		**Time frame: Since the initial planning of the work**	
1	All support for the present manuscript (e.g., funding, provision of study materials, medical writing, article processing charges, etc.) **No time limit for this item.**	__X_None	
		**Time frame: past 36 months**	
2	Grants or contracts from any entity (if not indicated in item #1 above).	__X__None	
3	Royalties or licenses	__X__None	
4	Consulting fees	__X__None	

5	Payment or honoraria for lectures, presentations, speakers bureaus, manuscript writing or educational events	_X___None	
6	Payment for expert testimony	_X___None	
7	Support for attending meetings and/or travel	__X__None	
8	Patents planned, issued or pending	_X___None	
9	Participation on a Data Safety Monitoring Board or Advisory Board	_X___None	
10	Leadership or fiduciary role in other board, society, committee or advocacy group, paid or unpaid	_X___None	
11	Stock or stock options	X____None	
12	Receipt of equipment, materials, drugs, medical writing, gifts or other services	_X___None	
13	Other financial or non-financial interests	__X__None	

Please place an "X" next to the following statement to indicate your agreement:

X___ I certify that I have answered every question and have not altered the wording of any of the questions on this form.

ICMJE DISCLOSURE FORM

Date:_______________4/29/2021____
Your Name:____________Xiaoyun Li____
Manuscript Title:_______ Plasma 1,3-beta-D-glucan levels are associated with host inflammatory responses and predict adverse clinical outcomes in critical illness _______________________________________
Manuscript number (if known):_______ 141277-INS-RG-1_______________________________________

In the interest of transparency, we ask you to disclose all relationships/activities/interests listed below that are related to the content of your manuscript. "Related" means any relation with for-profit or not-for-profit third parties whose interests may be affected by the content of the manuscript. Disclosure represents a commitment to transparency and does not necessarily indicate a bias. If you are in doubt about whether to list a relationship/activity/interest, it is preferable that you do so.

The following questions apply to the author's relationships/activities/interests as they relate to the current manuscript only.

The author's relationships/activities/interests should be defined broadly. For example, if your manuscript pertains to the epidemiology of hypertension, you should declare all relationships with manufacturers of antihypertensive medication, even if that medication is not mentioned in the manuscript.

In item #1 below, report all support for the work reported in this manuscript without time limit. For all other items, the time frame for disclosure is the past 36 months.

		**Name all entities with whom you have this relationship or indicate none (add rows as needed)**	**Specifications/Comments (e.g., if payments were made to you or to your institution)**
		**Time frame: Since the initial planning of the work**	
1	All support for the present manuscript (e.g., funding, provision of study materials, medical writing, article processing charges, etc.) **No time limit for this item.**	__X_None	
		**Time frame: past 36 months**	
2	Grants or contracts from any entity (if not indicated in item #1 above).	__X__None	
3	Royalties or licenses	__X__None	
4	Consulting fees	__X__None	

5	Payment or honoraria for lectures, presentations, speakers bureaus, manuscript writing or educational events	_X___None	
6	Payment for expert testimony	_X___None	
7	Support for attending meetings and/or travel	__X__None	
8	Patents planned, issued or pending	_X___None	
9	Participation on a Data Safety Monitoring Board or Advisory Board	_X___None	
10	Leadership or fiduciary role in other board, society, committee or advocacy group, paid or unpaid	_X___None	
11	Stock or stock options	X____None	
12	Receipt of equipment, materials, drugs, medical writing, gifts or other services	_X___None	
13	Other financial or non-financial interests	__X__None	

Please place an "X" next to the following statement to indicate your agreement:

X___ I certify that I have answered every question and have not altered the wording of any of the questions on this form.

ICMJE DISCLOSURE FORM

Date:_______________4/29/2021____
Your Name:____________MARINA LEVOCHKINA____
Manuscript Title:_______ Plasma 1,3-beta-D-glucan levels are associated with host inflammatory responses and predict adverse clinical outcomes in critical illness ____
Manuscript number (if known):_______ 141277-INS-RG-1____

In the interest of transparency, we ask you to disclose all relationships/activities/interests listed below that are related to the content of your manuscript. "Related" means any relation with for-profit or not-for-profit third parties whose interests may be affected by the content of the manuscript. Disclosure represents a commitment to transparency and does not necessarily indicate a bias. If you are in doubt about whether to list a relationship/activity/interest, it is preferable that you do so.

The following questions apply to the author's relationships/activities/interests as they relate to the current manuscript only.

The author's relationships/activities/interests should be defined broadly. For example, if your manuscript pertains to the epidemiology of hypertension, you should declare all relationships with manufacturers of antihypertensive medication, even if that medication is not mentioned in the manuscript.

In item #1 below, report all support for the work reported in this manuscript without time limit. For all other items, the time frame for disclosure is the past 36 months.

		Name all entities with whom you have this relationship or indicate none (add rows as needed)	Specifications/Comments (e.g., if payments were made to you or to your institution)
**Time frame: Since the initial planning of the work**			
1	All support for the present manuscript (e.g., funding, provision of study materials, medical writing, article processing charges, etc.) **No time limit for this item.**	__X_None	
**Time frame: past 36 months**			
2	Grants or contracts from any entity (if not indicated in item #1 above).	__X__None	
3	Royalties or licenses	__X__None	
4	Consulting fees	__X__None	

5	Payment or honoraria for lectures, presentations, speakers bureaus, manuscript writing or educational events	_X___None	
6	Payment for expert testimony	_X___None	
7	Support for attending meetings and/or travel	__X__None	
8	Patents planned, issued or pending	_X___None	
9	Participation on a Data Safety Monitoring Board or Advisory Board	_X___None	
10	Leadership or fiduciary role in other board, society, committee or advocacy group, paid or unpaid	_X___None	
11	Stock or stock options	X____None	
12	Receipt of equipment, materials, drugs, medical writing, gifts or other services	_X___None	
13	Other financial or non-financial interests	__X__None	

Please place an "X" next to the following statement to indicate your agreement:

X___ I certify that I have answered every question and have not altered the wording of any of the questions on this form.

ICMJE DISCLOSURE FORM

Date:_______________4/29/2021____
Your Name:____________AMY WAGNER____
Manuscript Title:_______ Plasma 1,3-beta-D-glucan levels are associated with host inflammatory responses and predict adverse clinical outcomes in critical illness ____
Manuscript number (if known):_______ 141277-INS-RG-1____

In the interest of transparency, we ask you to disclose all relationships/activities/interests listed below that are related to the content of your manuscript. "Related" means any relation with for-profit or not-for-profit third parties whose interests may be affected by the content of the manuscript. Disclosure represents a commitment to transparency and does not necessarily indicate a bias. If you are in doubt about whether to list a relationship/activity/interest, it is preferable that you do so.

The following questions apply to the author's relationships/activities/interests as they relate to the current manuscript only.

The author's relationships/activities/interests should be defined broadly. For example, if your manuscript pertains to the epidemiology of hypertension, you should declare all relationships with manufacturers of antihypertensive medication, even if that medication is not mentioned in the manuscript.

In item #1 below, report all support for the work reported in this manuscript without time limit. For all other items, the time frame for disclosure is the past 36 months.

		Name all entities with whom you have this relationship or indicate none (add rows as needed)	Specifications/Comments (e.g., if payments were made to you or to your institution)
		**Time frame: Since the initial planning of the work**	
1	All support for the present manuscript (e.g., funding, provision of study materials, medical writing, article processing charges, etc.) **No time limit for this item.**	__X_None	
		**Time frame: past 36 months**	
2	Grants or contracts from any entity (if not indicated in item #1 above).	__X__None	
3	Royalties or licenses	__X__None	
4	Consulting fees	__X__None	

5	Payment or honoraria for lectures, presentations, speakers bureaus, manuscript writing or educational events	_X___None	
6	Payment for expert testimony	_X___None	
7	Support for attending meetings and/or travel	__X__None	
8	Patents planned, issued or pending	_X___None	
9	Participation on a Data Safety Monitoring Board or Advisory Board	_X___None	
10	Leadership or fiduciary role in other board, society, committee or advocacy group, paid or unpaid	_X___None	
11	Stock or stock options	X____None	
12	Receipt of equipment, materials, drugs, medical writing, gifts or other services	_X___None	
13	Other financial or non-financial interests	__X__None	

Please place an "X" next to the following statement to indicate your agreement:

X___ I certify that I have answered every question and have not altered the wording of any of the questions on this form.

ICMJE DISCLOSURE FORM

Date:_______________4/29/2021____
Your Name:____________CAITLIN SCHAEFFER____
Manuscript Title:_______ Plasma 1,3-beta-D-glucan levels are associated with host inflammatory responses and predict adverse clinical outcomes in critical illness ____
Manuscript number (if known):_______ 141277-INS-RG-1____

In the interest of transparency, we ask you to disclose all relationships/activities/interests listed below that are related to the content of your manuscript. "Related" means any relation with for-profit or not-for-profit third parties whose interests may be affected by the content of the manuscript. Disclosure represents a commitment to transparency and does not necessarily indicate a bias. If you are in doubt about whether to list a relationship/activity/interest, it is preferable that you do so.

The following questions apply to the author's relationships/activities/interests as they relate to the current manuscript only.

The author's relationships/activities/interests should be defined broadly. For example, if your manuscript pertains to the epidemiology of hypertension, you should declare all relationships with manufacturers of antihypertensive medication, even if that medication is not mentioned in the manuscript.

In item #1 below, report all support for the work reported in this manuscript without time limit. For all other items, the time frame for disclosure is the past 36 months.

		**Name all entities with whom you have this relationship or indicate none (add rows as needed)**	**Specifications/Comments (e.g., if payments were made to you or to your institution)**
		**Time frame: Since the initial planning of the work**	
1	All support for the present manuscript (e.g., funding, provision of study materials, medical writing, article processing charges, etc.) **No time limit for this item.**	__X_None	
		**Time frame: past 36 months**	
2	Grants or contracts from any entity (if not indicated in item #1 above).	__X__None	
3	Royalties or licenses	__X__None	
4	Consulting fees	__X__None	

5	Payment or honoraria for lectures, presentations, speakers bureaus, manuscript writing or educational events	_X___None	
6	Payment for expert testimony	_X___None	
7	Support for attending meetings and/or travel	__X__None	
8	Patents planned, issued or pending	_X___None	
9	Participation on a Data Safety Monitoring Board or Advisory Board	_X___None	
10	Leadership or fiduciary role in other board, society, committee or advocacy group, paid or unpaid	_X___None	
11	Stock or stock options	X____None	
12	Receipt of equipment, materials, drugs, medical writing, gifts or other services	_X___None	
13	Other financial or non-financial interests	__X__None	

Please place an "X" next to the following statement to indicate your agreement:

X___ I certify that I have answered every question and have not altered the wording of any of the questions on this form.

ICMJE DISCLOSURE FORM

Date:_______________4/29/2021____
Your Name:____________JOHN VILLANDRE____
Manuscript Title:_______ Plasma 1,3-beta-D-glucan levels are associated with host inflammatory responses and predict adverse clinical outcomes in critical illness ______________________________________
Manuscript number (if known):_______ 141277-INS-RG-1____

In the interest of transparency, we ask you to disclose all relationships/activities/interests listed below that are related to the content of your manuscript. "Related" means any relation with for-profit or not-for-profit third parties whose interests may be affected by the content of the manuscript. Disclosure represents a commitment to transparency and does not necessarily indicate a bias. If you are in doubt about whether to list a relationship/activity/interest, it is preferable that you do so.

The following questions apply to the author's relationships/activities/interests as they relate to the current manuscript only.

The author's relationships/activities/interests should be defined broadly. For example, if your manuscript pertains to the epidemiology of hypertension, you should declare all relationships with manufacturers of antihypertensive medication, even if that medication is not mentioned in the manuscript.

In item #1 below, report all support for the work reported in this manuscript without time limit. For all other items, the time frame for disclosure is the past 36 months.

		**Name all entities with whom you have this relationship or indicate none (add rows as needed)**	**Specifications/Comments (e.g., if payments were made to you or to your institution)**
		**Time frame: Since the initial planning of the work**	
1	All support for the present manuscript (e.g., funding, provision of study materials, medical writing, article processing charges, etc.) **No time limit for this item.**	__X_None	
		**Time frame: past 36 months**	
2	Grants or contracts from any entity (if not indicated in item #1 above).	__X__None	
3	Royalties or licenses	__X__None	
4	Consulting fees	__X__None	

5	Payment or honoraria for lectures, presentations, speakers bureaus, manuscript writing or educational events	_X___None	
6	Payment for expert testimony	_X___None	
7	Support for attending meetings and/or travel	__X__None	
8	Patents planned, issued or pending	_X___None	
9	Participation on a Data Safety Monitoring Board or Advisory Board	_X___None	
10	Leadership or fiduciary role in other board, society, committee or advocacy group, paid or unpaid	_X___None	
11	Stock or stock options	X____None	
12	Receipt of equipment, materials, drugs, medical writing, gifts or other services	_X___None	
13	Other financial or non-financial interests	__X__None	

Please place an "X" next to the following statement to indicate your agreement:

X___ I certify that I have answered every question and have not altered the wording of any of the questions on this form.

ICMJE DISCLOSURE FORM

Date:_______________4/29/2021____
Your Name:____________RUI GUO____
Manuscript Title:_______ Plasma 1,3-beta-D-glucan levels are associated with host inflammatory responses and predict adverse clinical outcomes in critical illness _______________________________________
Manuscript number (if known):_______ 141277-INS-RG-1____

In the interest of transparency, we ask you to disclose all relationships/activities/interests listed below that are related to the content of your manuscript. "Related" means any relation with for-profit or not-for-profit third parties whose interests may be affected by the content of the manuscript. Disclosure represents a commitment to transparency and does not necessarily indicate a bias. If you are in doubt about whether to list a relationship/activity/interest, it is preferable that you do so.

The following questions apply to the author's relationships/activities/interests as they relate to the current manuscript only.

The author's relationships/activities/interests should be defined broadly. For example, if your manuscript pertains to the epidemiology of hypertension, you should declare all relationships with manufacturers of antihypertensive medication, even if that medication is not mentioned in the manuscript.

In item #1 below, report all support for the work reported in this manuscript without time limit. For all other items, the time frame for disclosure is the past 36 months.

		Name all entities with whom you have this relationship or indicate none (add rows as needed)	Specifications/Comments (e.g., if payments were made to you or to your institution)
**Time frame: Since the initial planning of the work**			
1	All support for the present manuscript (e.g., funding, provision of study materials, medical writing, article processing charges, etc.) **No time limit for this item.**	__X_None	
**Time frame: past 36 months**			
2	Grants or contracts from any entity (if not indicated in item #1 above).	__X__None	
3	Royalties or licenses	__X__None	
4	Consulting fees	__X__None	

5	Payment or honoraria for lectures, presentations, speakers bureaus, manuscript writing or educational events	_X___None	
6	Payment for expert testimony	_X___None	
7	Support for attending meetings and/or travel	__X__None	
8	Patents planned, issued or pending	_X___None	
9	Participation on a Data Safety Monitoring Board or Advisory Board	_X___None	
10	Leadership or fiduciary role in other board, society, committee or advocacy group, paid or unpaid	_X___None	
11	Stock or stock options	X____None	
12	Receipt of equipment, materials, drugs, medical writing, gifts or other services	_X___None	
13	Other financial or non-financial interests	__X__None	

Please place an "X" next to the following statement to indicate your agreement:

X___ I certify that I have answered every question and have not altered the wording of any of the questions on this form.

ICMJE DISCLOSURE FORM

Date:_______________4/29/2021____
Your Name:____________JOHN EVANKOVICH____
Manuscript Title:_______ Plasma 1,3-beta-D-glucan levels are associated with host inflammatory responses and predict adverse clinical outcomes in critical illness _______________________________________
Manuscript number (if known):_______ 141277-INS-RG-1____

In the interest of transparency, we ask you to disclose all relationships/activities/interests listed below that are related to the content of your manuscript. "Related" means any relation with for-profit or not-for-profit third parties whose interests may be affected by the content of the manuscript. Disclosure represents a commitment to transparency and does not necessarily indicate a bias. If you are in doubt about whether to list a relationship/activity/interest, it is preferable that you do so.

The following questions apply to the author's relationships/activities/interests as they relate to the current manuscript only.

The author's relationships/activities/interests should be defined broadly. For example, if your manuscript pertains to the epidemiology of hypertension, you should declare all relationships with manufacturers of antihypertensive medication, even if that medication is not mentioned in the manuscript.

In item #1 below, report all support for the work reported in this manuscript without time limit. For all other items, the time frame for disclosure is the past 36 months.

		**Name all entities with whom you have this relationship or indicate none (add rows as needed)**	**Specifications/Comments (e.g., if payments were made to you or to your institution)**
		**Time frame: Since the initial planning of the work**	
1	All support for the present manuscript (e.g., funding, provision of study materials, medical writing, article processing charges, etc.) **No time limit for this item.**	__X_None	
		**Time frame: past 36 months**	
2	Grants or contracts from any entity (if not indicated in item #1 above).	NIH	
3	Royalties or licenses	__X__None	
4	Consulting fees	__X__None	

5	Payment or honoraria for lectures, presentations, speakers bureaus, manuscript writing or educational events	_X___None	
6	Payment for expert testimony	_X___None	
7	Support for attending meetings and/or travel	__X__None	
8	Patents planned, issued or pending	_X___None	
9	Participation on a Data Safety Monitoring Board or Advisory Board	_X___None	
10	Leadership or fiduciary role in other board, society, committee or advocacy group, paid or unpaid	_X___None	
11	Stock or stock options	X____None	
12	Receipt of equipment, materials, drugs, medical writing, gifts or other services	_X___None	
13	Other financial or non-financial interests	__X__None	

Please place an "X" next to the following statement to indicate your agreement:

X___ I certify that I have answered every question and have not altered the wording of any of the questions on this form.

ICMJE DISCLOSURE FORM

Date:_______________4/29/2021____
Your Name:____________WILLIAM BAIN____
Manuscript Title:_______ Plasma 1,3-beta-D-glucan levels are associated with host inflammatory responses and predict adverse clinical outcomes in critical illness _______________________________________
Manuscript number (if known):_______ 141277-INS-RG-1_______________________________________

In the interest of transparency, we ask you to disclose all relationships/activities/interests listed below that are related to the content of your manuscript. "Related" means any relation with for-profit or not-for-profit third parties whose interests may be affected by the content of the manuscript. Disclosure represents a commitment to transparency and does not necessarily indicate a bias. If you are in doubt about whether to list a relationship/activity/interest, it is preferable that you do so.

The following questions apply to the author's relationships/activities/interests as they relate to the current manuscript only.

The author's relationships/activities/interests should be defined broadly. For example, if your manuscript pertains to the epidemiology of hypertension, you should declare all relationships with manufacturers of antihypertensive medication, even if that medication is not mentioned in the manuscript.

In item #1 below, report all support for the work reported in this manuscript without time limit. For all other items, the time frame for disclosure is the past 36 months.

		**Name all entities with whom you have this relationship or indicate none (add rows as needed)**	**Specifications/Comments (e.g., if payments were made to you or to your institution)**
		**Time frame: Since the initial planning of the work**	
1	All support for the present manuscript (e.g., funding, provision of study materials, medical writing, article processing charges, etc.) **No time limit for this item.**	__X_None	
		**Time frame: past 36 months**	
2	Grants or contracts from any entity (if not indicated in item #1 above).	NIH	
		VETERANS AFFAIRS	
3	Royalties or licenses	__X__None	
4	Consulting fees	__X__None	

5	Payment or honoraria for lectures, presentations, speakers bureaus, manuscript writing or educational events	_X___None	
6	Payment for expert testimony	_X___None	
7	Support for attending meetings and/or travel	__X__None	
8	Patents planned, issued or pending	_X___None	
9	Participation on a Data Safety Monitoring Board or Advisory Board	_X___None	
10	Leadership or fiduciary role in other board, society, committee or advocacy group, paid or unpaid	_X___None	
11	Stock or stock options	X____None	
12	Receipt of equipment, materials, drugs, medical writing, gifts or other services	_X___None	
13	Other financial or non-financial interests	__X__None	

Please place an "X" next to the following statement to indicate your agreement:

X___ I certify that I have answered every question and have not altered the wording of any of the questions on this form.

ICMJE DISCLOSURE FORM

Date:_______________4/29/2021____
Your Name:____________FARAAZ SHAH____
Manuscript Title:_______ Plasma 1,3-beta-D-glucan levels are associated with host inflammatory responses and predict adverse clinical outcomes in critical illness _______________________________________
Manuscript number (if known):_______ 141277-INS-RG-1_______________________________________

In the interest of transparency, we ask you to disclose all relationships/activities/interests listed below that are related to the content of your manuscript. "Related" means any relation with for-profit or not-for-profit third parties whose interests may be affected by the content of the manuscript. Disclosure represents a commitment to transparency and does not necessarily indicate a bias. If you are in doubt about whether to list a relationship/activity/interest, it is preferable that you do so.

The following questions apply to the author's relationships/activities/interests as they relate to the current manuscript only.

The author's relationships/activities/interests should be defined broadly. For example, if your manuscript pertains to the epidemiology of hypertension, you should declare all relationships with manufacturers of antihypertensive medication, even if that medication is not mentioned in the manuscript.

In item #1 below, report all support for the work reported in this manuscript without time limit. For all other items, the time frame for disclosure is the past 36 months.

		Name all entities with whom you have this relationship or indicate none (add rows as needed)	Specifications/Comments (e.g., if payments were made to you or to your institution)
		**Time frame: Since the initial planning of the work**	
1	All support for the present manuscript (e.g., funding, provision of study materials, medical writing, article processing charges, etc.) **No time limit for this item.**	__X_None	
		**Time frame: past 36 months**	
2	Grants or contracts from any entity (if not indicated in item #1 above).	NIH	
		VETERANS AFFAIRS	
3	Royalties or licenses	__X__None	
4	Consulting fees	__X__None	

5	Payment or honoraria for lectures, presentations, speakers bureaus, manuscript writing or educational events	_X___None	
6	Payment for expert testimony	_X___None	
7	Support for attending meetings and/or travel	__X__None	
8	Patents planned, issued or pending	_X___None	
9	Participation on a Data Safety Monitoring Board or Advisory Board	_X___None	
10	Leadership or fiduciary role in other board, society, committee or advocacy group, paid or unpaid	_X___None	
11	Stock or stock options	X____None	
12	Receipt of equipment, materials, drugs, medical writing, gifts or other services	_X___None	
13	Other financial or non-financial interests	__X__None	

Please place an "X" next to the following statement to indicate your agreement:

X___ I certify that I have answered every question and have not altered the wording of any of the questions on this form.

ICMJE DISCLOSURE FORM

Date:_______________4/29/2021____
Your Name:____________YINGZE ZHANG____
Manuscript Title:_______ Plasma 1,3-beta-D-glucan levels are associated with host inflammatory responses and predict adverse clinical outcomes in critical illness ____
Manuscript number (if known):_______ 141277-INS-RG-1____

In the interest of transparency, we ask you to disclose all relationships/activities/interests listed below that are related to the content of your manuscript. "Related" means any relation with for-profit or not-for-profit third parties whose interests may be affected by the content of the manuscript. Disclosure represents a commitment to transparency and does not necessarily indicate a bias. If you are in doubt about whether to list a relationship/activity/interest, it is preferable that you do so.

The following questions apply to the author's relationships/activities/interests as they relate to the current manuscript only.

The author's relationships/activities/interests should be defined broadly. For example, if your manuscript pertains to the epidemiology of hypertension, you should declare all relationships with manufacturers of antihypertensive medication, even if that medication is not mentioned in the manuscript.

In item #1 below, report all support for the work reported in this manuscript without time limit. For all other items, the time frame for disclosure is the past 36 months.

		**Name all entities with whom you have this relationship or indicate none (add rows as needed)**	**Specifications/Comments (e.g., if payments were made to you or to your institution)**
		**Time frame: Since the initial planning of the work**	
1	All support for the present manuscript (e.g., funding, provision of study materials, medical writing, article processing charges, etc.) **No time limit for this item.**	__X_None	
		**Time frame: past 36 months**	
2	Grants or contracts from any entity (if not indicated in item #1 above).	NIH	
3	Royalties or licenses	__X__None	
4	Consulting fees	__X__None	

5	Payment or honoraria for lectures, presentations, speakers bureaus, manuscript writing or educational events	_X___None	
6	Payment for expert testimony	_X___None	
7	Support for attending meetings and/or travel	__X__None	
8	Patents planned, issued or pending	_X___None	
9	Participation on a Data Safety Monitoring Board or Advisory Board	_X___None	
10	Leadership or fiduciary role in other board, society, committee or advocacy group, paid or unpaid	_X___None	
11	Stock or stock options	X____None	
12	Receipt of equipment, materials, drugs, medical writing, gifts or other services	_X___None	
13	Other financial or non-financial interests	__X__None	

Please place an "X" next to the following statement to indicate your agreement:

X___ I certify that I have answered every question and have not altered the wording of any of the questions on this form.

ICMJE DISCLOSURE FORM

Date:_______________4/29/2021____
Your Name:____________BARBARA METHE____
Manuscript Title:_______ Plasma 1,3-beta-D-glucan levels are associated with host inflammatory responses and predict adverse clinical outcomes in critical illness _______________________________________
Manuscript number (if known):_______ 141277-INS-RG-1____

In the interest of transparency, we ask you to disclose all relationships/activities/interests listed below that are related to the content of your manuscript. "Related" means any relation with for-profit or not-for-profit third parties whose interests may be affected by the content of the manuscript. Disclosure represents a commitment to transparency and does not necessarily indicate a bias. If you are in doubt about whether to list a relationship/activity/interest, it is preferable that you do so.

The following questions apply to the author's relationships/activities/interests as they relate to the current manuscript only.

The author's relationships/activities/interests should be defined broadly. For example, if your manuscript pertains to the epidemiology of hypertension, you should declare all relationships with manufacturers of antihypertensive medication, even if that medication is not mentioned in the manuscript.

In item #1 below, report all support for the work reported in this manuscript without time limit. For all other items, the time frame for disclosure is the past 36 months.

		**Name all entities with whom you have this relationship or indicate none (add rows as needed)**	**Specifications/Comments (e.g., if payments were made to you or to your institution)**
		**Time frame: Since the initial planning of the work**	
1	All support for the present manuscript (e.g., funding, provision of study materials, medical writing, article processing charges, etc.) **No time limit for this item.**	__X_None	
		**Time frame: past 36 months**	
2	Grants or contracts from any entity (if not indicated in item #1 above).	NIH	
3	Royalties or licenses	__X__None	
4	Consulting fees	__X__None	

5	Payment or honoraria for lectures, presentations, speakers bureaus, manuscript writing or educational events	_X___None	
6	Payment for expert testimony	_X___None	
7	Support for attending meetings and/or travel	__X__None	
8	Patents planned, issued or pending	_X___None	
9	Participation on a Data Safety Monitoring Board or Advisory Board	_X___None	
10	Leadership or fiduciary role in other board, society, committee or advocacy group, paid or unpaid	_X___None	
11	Stock or stock options	X____None	
12	Receipt of equipment, materials, drugs, medical writing, gifts or other services	_X___None	
13	Other financial or non-financial interests	__X__None	

Please place an "X" next to the following statement to indicate your agreement:

X___ I certify that I have answered every question and have not altered the wording of any of the questions on this form.

ICMJE DISCLOSURE FORM

Date:_______________4/29/2021____
Your Name:____________PANAYIOTIS BENOS____
Manuscript Title:_______ Plasma 1,3-beta-D-glucan levels are associated with host inflammatory responses and predict adverse clinical outcomes in critical illness _______________________________________
Manuscript number (if known):_______ 141277-INS-RG-1____

In the interest of transparency, we ask you to disclose all relationships/activities/interests listed below that are related to the content of your manuscript. "Related" means any relation with for-profit or not-for-profit third parties whose interests may be affected by the content of the manuscript. Disclosure represents a commitment to transparency and does not necessarily indicate a bias. If you are in doubt about whether to list a relationship/activity/interest, it is preferable that you do so.

The following questions apply to the author's relationships/activities/interests as they relate to the current manuscript only.

The author's relationships/activities/interests should be defined broadly. For example, if your manuscript pertains to the epidemiology of hypertension, you should declare all relationships with manufacturers of antihypertensive medication, even if that medication is not mentioned in the manuscript.

In item #1 below, report all support for the work reported in this manuscript without time limit. For all other items, the time frame for disclosure is the past 36 months.

		**Name all entities with whom you have this relationship or indicate none (add rows as needed)**	**Specifications/Comments (e.g., if payments were made to you or to your institution)**
**Time frame: Since the initial planning of the work**			
1	All support for the present manuscript (e.g., funding, provision of study materials, medical writing, article processing charges, etc.) **No time limit for this item.**	__X_None	
**Time frame: past 36 months**			
2	Grants or contracts from any entity (if not indicated in item #1 above).	NIH	
3	Royalties or licenses	__X__None	
4	Consulting fees	__X__None	

5	Payment or honoraria for lectures, presentations, speakers bureaus, manuscript writing or educational events	_X___None	
6	Payment for expert testimony	_X___None	
7	Support for attending meetings and/or travel	__X__None	
8	Patents planned, issued or pending	_X___None	
9	Participation on a Data Safety Monitoring Board or Advisory Board	_X___None	
10	Leadership or fiduciary role in other board, society, committee or advocacy group, paid or unpaid	_X___None	
11	Stock or stock options	X____None	
12	Receipt of equipment, materials, drugs, medical writing, gifts or other services	_X___None	
13	Other financial or non-financial interests	__X__None	

Please place an "X" next to the following statement to indicate your agreement:

X___ I certify that I have answered every question and have not altered the wording of any of the questions on this form.

ICMJE DISCLOSURE FORM

Date:_______________4/29/2021____
Your Name:____________BRYAN MCVERRY____
Manuscript Title:_______ Plasma 1,3-beta-D-glucan levels are associated with host inflammatory responses and predict adverse clinical outcomes in critical illness _______________________________________
Manuscript number (if known):_______ 141277-INS-RG-1____

In the interest of transparency, we ask you to disclose all relationships/activities/interests listed below that are related to the content of your manuscript. "Related" means any relation with for-profit or not-for-profit third parties whose interests may be affected by the content of the manuscript. Disclosure represents a commitment to transparency and does not necessarily indicate a bias. If you are in doubt about whether to list a relationship/activity/interest, it is preferable that you do so.

The following questions apply to the author's relationships/activities/interests as they relate to the current manuscript only.

The author's relationships/activities/interests should be defined broadly. For example, if your manuscript pertains to the epidemiology of hypertension, you should declare all relationships with manufacturers of antihypertensive medication, even if that medication is not mentioned in the manuscript.

In item #1 below, report all support for the work reported in this manuscript without time limit. For all other items, the time frame for disclosure is the past 36 months.

		Name all entities with whom you have this relationship or indicate none (add rows as needed)	Specifications/Comments (e.g., if payments were made to you or to your institution)
		**Time frame: Since the initial planning of the work**	
1	All support for the present manuscript (e.g., funding, provision of study materials, medical writing, article processing charges, etc.) **No time limit for this item.**	__X_None	
		**Time frame: past 36 months**	
2	Grants or contracts from any entity (if not indicated in item #1 above).	NIH	
		Bayer Pharmaceuticals, Inc	
3	Royalties or licenses	__X__None	
4	Consulting fees	__X__None	

5	Payment or honoraria for lectures, presentations, speakers bureaus, manuscript writing or educational events	_X___None	
6	Payment for expert testimony	_X___None	
7	Support for attending meetings and/or travel	__X__None	
8	Patents planned, issued or pending	_X___None	
9	Participation on a Data Safety Monitoring Board or Advisory Board	_X___None	
10	Leadership or fiduciary role in other board, society, committee or advocacy group, paid or unpaid	_X___None	
11	Stock or stock options	X____None	
12	Receipt of equipment, materials, drugs, medical writing, gifts or other services	_X___None	
13	Other financial or non-financial interests	__X__None	

Please place an "X" next to the following statement to indicate your agreement:

X___ I certify that I have answered every question and have not altered the wording of any of the questions on this form.

ICMJE DISCLOSURE FORM

Date:_______________4/29/2021____
Your Name:____________Alison Morris____
Manuscript Title:_______ Plasma 1,3-beta-D-glucan levels are associated with host inflammatory responses and predict adverse clinical outcomes in critical illness ____
Manuscript number (if known):_______ 141277-INS-RG-1____

In the interest of transparency, we ask you to disclose all relationships/activities/interests listed below that are related to the content of your manuscript. "Related" means any relation with for-profit or not-for-profit third parties whose interests may be affected by the content of the manuscript. Disclosure represents a commitment to transparency and does not necessarily indicate a bias. If you are in doubt about whether to list a relationship/activity/interest, it is preferable that you do so.

The following questions apply to the author's relationships/activities/interests as they relate to the current manuscript only.

The author's relationships/activities/interests should be defined broadly. For example, if your manuscript pertains to the epidemiology of hypertension, you should declare all relationships with manufacturers of antihypertensive medication, even if that medication is not mentioned in the manuscript.

In item #1 below, report all support for the work reported in this manuscript without time limit. For all other items, the time frame for disclosure is the past 36 months.

		**Name all entities with whom you have this relationship or indicate none (add rows as needed)**	**Specifications/Comments (e.g., if payments were made to you or to your institution)**
		**Time frame: Since the initial planning of the work**	
1	All support for the present manuscript (e.g., funding, provision of study materials, medical writing, article processing charges, etc.) **No time limit for this item.**	__X_None	
		**Time frame: past 36 months**	
2	Grants or contracts from any entity (if not indicated in item #1 above).	NIH	
3	Royalties or licenses	__X__None	
4	Consulting fees	__X__None	

5	Payment or honoraria for lectures, presentations, speakers bureaus, manuscript writing or educational events	_X___None	
6	Payment for expert testimony	_X___None	
7	Support for attending meetings and/or travel	__X__None	
8	Patents planned, issued or pending	_X___None	
9	Participation on a Data Safety Monitoring Board or Advisory Board	_X___None	
10	Leadership or fiduciary role in other board, society, committee or advocacy group, paid or unpaid	_X___None	
11	Stock or stock options	X____None	
12	Receipt of equipment, materials, drugs, medical writing, gifts or other services	_X___None	
13	Other financial or non-financial interests	__X__None	

Please place an "X" next to the following statement to indicate your agreement:

X___ I certify that I have answered every question and have not altered the wording of any of the questions on this form.
